# Genetic polymorphisms and platinum-induced hematological toxicity: a systematic review

**DOI:** 10.3389/fphar.2024.1445328

**Published:** 2024-08-21

**Authors:** Yi Zheng, Mimi Tang, Zheng Deng, Pei Cai

**Affiliations:** ^1^ Hunan Provincial Maternal and Child Health Care Hospital, Changsha, China; ^2^ Department of Pharmacy, Xiangya Hospital, Central South University, Changsha, China; ^3^ Hunan Institute for Tuberculosis Control and Hunan Chest Hospital, Changsha, China; ^4^ Hunan Chest Hospital, Changsha, China

**Keywords:** platinum, chemotherapy, hematological toxicity, polymorphisms, pharmacogenomics

## Abstract

**Background:**

Platinum-based chemotherapy bring severe hematological toxicity that can lead to dose reduction or discontinuation of therapy. Genetic variations have been reported to influence the risk and extent of hematological toxicity; however, the results are controversial and a comprehensive overview is lacking. This systematic review aimed to identify genetic biomarkers of platinum-induced hematological toxicity.

**Method:**

Pubmed, Embase and Web of science database were systematically reviewed for studies that evaluated the association of genetic variants and platinum-related hematological toxicity in tumor patients with no prior history of chemotherapy or radiation, published from inception to the 28th of January 2022. The studies should have specific toxicity scoring system as well as defined toxicity end-point. The quality of reporting was assessed using the Strengthening the Reporting of Genetic Association Studies (STREGA) checklist. Results were summarized using narrative synthesis.

**Results:**

83 studies were eligible with over 682 single-nucleotide polymorphisms across 110 genes. The results are inconsistent and diverse with methodological issues including insufficient sample size, population stratification, various treatment schedule and toxicity end-point, and inappropriate statistics. 11 SNPs from 10 genes (*ABCB1 rs1128503, GSTP1 rs1695, GSTM1 gene deletion, ERCC1 rs11615, ERCC1 rs3212986, ERCC2 rs238406, XPC rs2228001, XPCC1 rs25487, MTHFR rs1801133, MDM2 rs2279744, TP53 rs1042522*) had consistent results in more than two independent populations. Among them, *GSTP1* rs1695, *ERCC1* rs11615, *ERCC1* rs3212986, and *XRCC1* rs25487 present the most promising results.

**Conclusion:**

Even though the results are inconsistent and several methodological concerns exist, this systematic review identified several genetic variations that deserve validation in well-defined studies with larger sample size and robust methodology.

**Systematic Review Registration:**

https://www.crd.york.ac.uk/, identifier CRD42021234164.

## 1 Introduction

Platinum agents, including cisplatin, carboplatin and oxaliplatin, are used effectively against various tumor diseases either as monotherapy or in combination with other chemotherapeutics, radiation therapy and/or surgery. However, they display a range of severe side effects due to their poor selectivity for cancerous tissue over normal tissue. Hematological toxicity caused by platinum drugs are those that affect bone marrow function and blood cell production, characterized by leukopenia, neutropenia, thrombocytopenia, and anemia (Oun et al., 2018). Leukopenia or neutropenia, can leave patients susceptible to infections. Platinum-induced anemia is persisting erythropoietin deficiency state correlated with renal tubular dysfunction (Wood and Hrushesky, 1995). Acute myelosuppression occurs shortly after chemotherapy, while residual bone marrow injury manifested by a decrease in hematopoietic stem cell reserves or a myelodysplastic syndrome (Wang et al., 2006).

All three platinum drugs can cause some form of hematological toxicity, and myelosuppression is the dose-limiting toxicity of carboplatin. In the majority of cases, neither cisplatin nor oxaliplatin is associated with severe myelosuppression (Rabik and Dolan, 2007). Carboplatin induced myelosuppression resulted in neutropenia and thrombocytopenia. Severe (grade 3 or 4) neutropenia occurs in approximately 18% of carboplatin-treated patients, whereas severe thrombocytopenia occurs in approximately 25% of cases (Go and Adjei, 1999). Cisplatin-induced hematological toxicity is usually mild at intermittent doses of 50–60 mg/m^2^ and myelosuppression presents in 25%–30% of patients (Prestayko et al., 1979). Myelosuppression caused by oxaliplatin is generally mild. Grade 3/4 anemia, neutropenia and thrombocytopenia are observed in only 2%–3% of patients (Hartmann and Lipp, 2003). Hematological toxicity aggravates when platinum agents were combined with other cytotoxic drugs. The degree of hematological toxicity varies upon different chemotherapy regimen. For example, hematological toxicity was more profound in lung cancer patients treated with platinum agents plus gemcitabine (Fisher and D’Orazio, 2000; Schiller et al., 2002).

**FIGURE 1:**
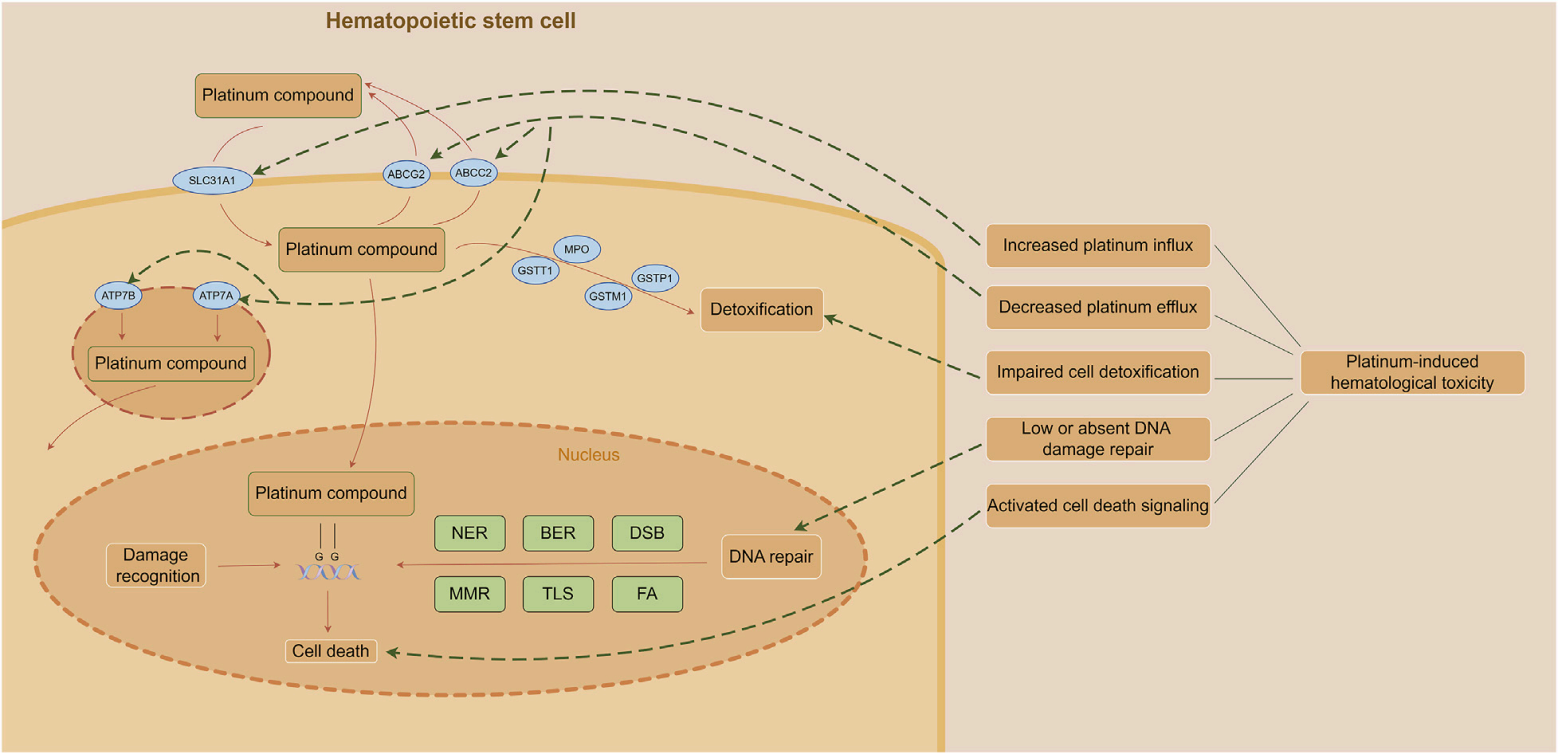
Mechanism of platinum-induced hematological toxicity. (The figure was made by Figdraw) Abbreviations: BER, base excision repair; DSB, double strand break repair; FA, fanconi anemia pathway; MMR, mismatch repair; NER, nucleotide excision repair; TLS, translesion DNA synthesis.

Inhibition of cell proliferation is one of the major causes of platinum-induced myelosuppression and related complications. The cytotoxicity of platinum on hematopoietic stem cells is attributed to its highly reactive hydrated platinum complex that binds to DNA and form intra- and inter-strand crosslinks; thereby produce subsequent interference with DNA transcription and/or DNA replication (Das et al., 2008). The generation in oxidative stress is also responsible for platinum-induced bone marrow toxicity (Basu et al., 2015). Increased platinum influx, decreased platinum efflux, impaired cell detoxification, low or absent DNA damage repair and activated cell death signaling may be the reasons of platinum-induced hematological toxicity (Shaloam and Paul, 2014) (Figure 1).

Identifying patients at greatest risk for these complications would be clinically useful for selecting patients for chemotherapy and planning the frequency of monitor and clinical treatment with colony-stimulating factor. Risk factors for hematological toxicities include kidney function, age, drug doses, combination chemotherapy, a poor performance status and prior chemotherapy exposure (Hartmann and Lipp, 2003; Ouyang et al., 2013). Furthermore, genetic variations in genes encoding proteins involved in pharmacokinetic and pharmacodynamic processes influence the occurrence and extent of adverse reactions (Zheng et al., 2020). Although several genetic polymorphisms have been identified to influence platinum-induced hematological toxicity, a comprehensive overview is lacking. We here provide an overview to identify which genetic variants consistently associated with hematological toxicity and discuss limitations of current pharmacogenetic analyses and formulate directions for further research.

## 2 Methods

### 2.1 Study eligibility

The systematic review was reported according to the Preferred Reporting Items for Systematic reviews and Meta-Analyses (PRISMA) checklist (Page et al., 2021) (Supplementary Table S1). The protocol was registered in the international prospective register of systematic reviews (PROSPERO; Registered number: CRD42021234164). The inclusion criteria were (Oun et al., 2018): studies that focus on the association between hematological toxicity and genetic polymorphisms (Wood and Hrushesky, 1995); studies including cancer patients using platinum-containing chemotherapy (Wang et al., 2006); studies that have specific toxicity scoring system and defined toxicity end-point. The exclusion criteria include the followings (Oun et al., 2018): preclinical studies (animal experiment or *in vitro* studies) (Wood and Hrushesky, 1995); studies in which patients were treated with concurrent radiotherapy (Wang et al., 2006); studies that were non-English, case report, review or meta-analysis (Rabik and Dolan, 2007); studies in which patients have prior history of chemotherapy and/or radiation.

### 2.2 Search strategy

PubMed/MEDLINE, EMBASE and Web of Science were searched for publications from inception to the 28th of January 2022. The literature search was conducted using Medical Subject Headings and combinations of relevant keywords. The detailed search strategy can be found in Supplementary Table S2. Additional research papers were identified by screening the reference sections of included articles. Two authors (YZ and MT) independently performed the data screening. Disagreements were consulted with a third arbiter (ZD).

### 2.3 Quality assessment

The quality of the studies will be assessed using a scoring system modified from a previously published study (Leusink et al., 2016) based on STREGA recommendations (Little et al., 2009) Supplementary Table S3. The scoring system contains ten items on five domains: clinical information, genotyping, study population origin, sample size and statistical correction for multiple testing, and study analysis. Each study included in this review was assessed for quality as good (overall quality score:7-10), moderate (overall quality score:4-6), or poor (overall quality score≤3) based on scores. Two reviewers (YZ and MT) will assess the quality independently and a third reviewer (PC) will be consulted in case of disagreement.

### 2.4 Data collection and analysis

The following data were extracted from each publication by two authors (YZ and MT): author, year, source of study (reference), sample size, ethnicity, type of cancer, number of treatment cycles, treatment schedule, toxicity scoring system, defined toxicity end-point, genetic polymorphisms involved and main study results.

Due to the heterogeneity in the patient population, treatment schedule, outcome definitions, meta-analysis was not appropriate. Studies were analyzed using a narrative synthesis approach.

## 3 Results

### 3.1 Study selection

**FIGURE 2:**
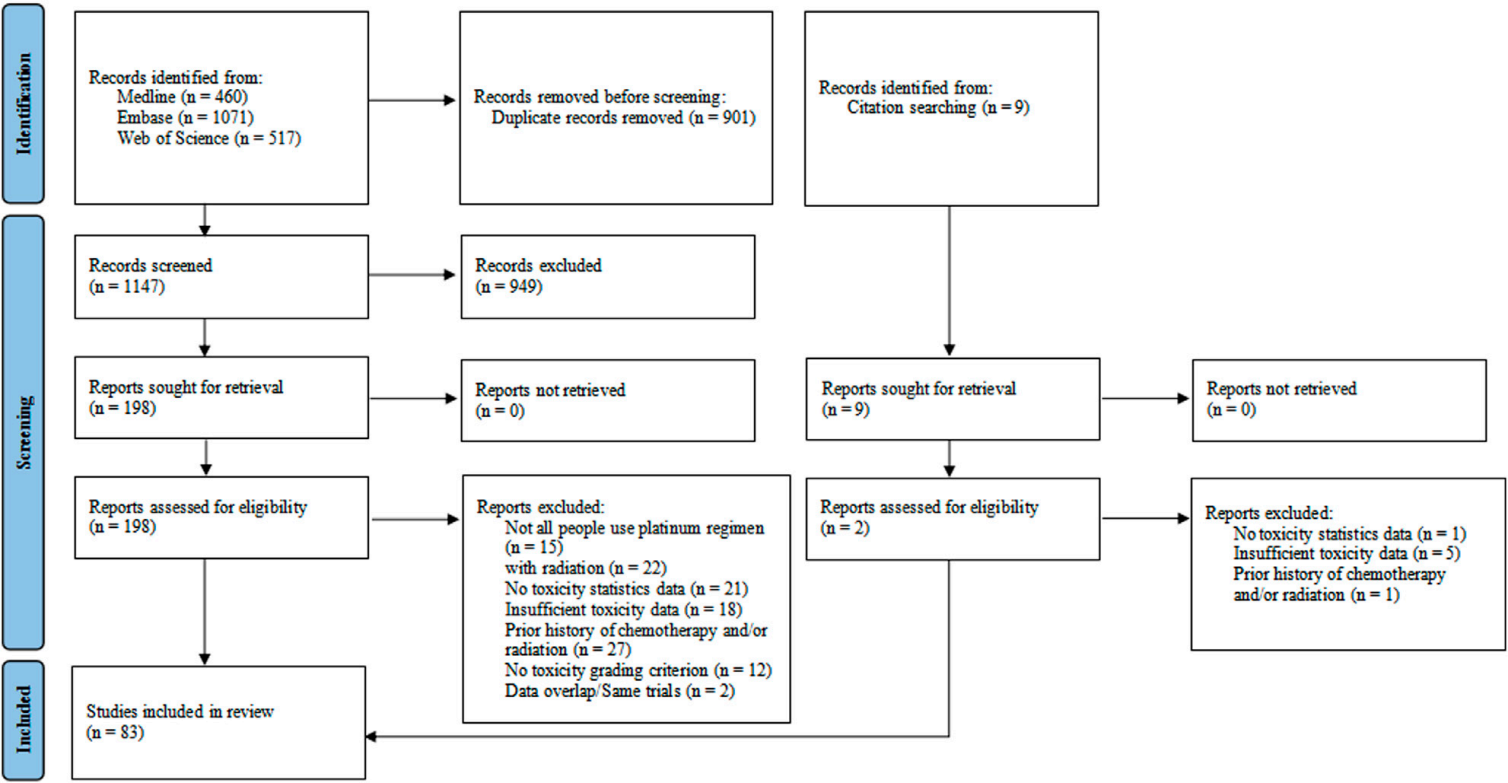
Flow diagram for study selection.

The initial search delivered 2057 articles; after removal of duplicates, 1156 abstracts were primarily screened of which 207 full-text articles remained. After reading the full-text, 83 studies were eventually included in the present systematic review. The article selection process is shown in Figure 2.

### 3.2 Study characteristics and quality assessment

The study characteristics of the 83 included articles are shown in Table 1, Supplementary Tables S4, S5. 75 studies were candidate gene studies. Three studies were genome-wide association studies (GWAS) (Low et al., 2013; Huang et al., 2015; Cao et al., 2016), three studies were whole-exome sequencing (Gréen et al., 2016; Svedberg et al., 2020; Björn et al., 2020a). 1 study was whole-genome sequencing (WGS) (Björn et al., 2020b). 1 study used targeted resequencing of 100 pharmacokinetics-related genes (Yoshihama et al., 2018).

**TABLE 1:** Overview of pharmacogenetic studies on platinum-induced hematological toxicity.

Authors, Year	Sample size cancer	Treatment	*Gene*	Toxicity endpoint	SNPs with significant association)	Total score
Isla et al. (2004)	62 NSCLC	DDP + TXT	*ERCC1, XPD, RRM1, MDR1*	Grade 2-4 anemia, leukopenia, neutropenia and thrombocytopenia	*ERCC2/XPD* rs13181 (Lys751Gln), *RRM1* rs12806698(-37C/A)	2
Han et al. (2007)	107 NSCLC	DDP + CPT-11	*ABCB1, ABCC2, ABCG2*	Grade 4 neutropenia	*ABCB1/MDR1* rs2032582 (G2677TA)	2
Kimcurran et al. (2011)	300 NSCLC	DDP/CBP + GEM/NVB/PTX	*ERCC1*	Grade 1-3 hematologic toxicity	No significant association	4
Marsh et al. (2007)	914 (Discovery cohort and validation cohort in ratio 2:1) Ovarian cancer	CBP + PTX/TXT	*ERCC1, XPD, XRCC1, ABCB1(MDR1), ABCC1, ABCC2, ABCG2, GSTP1, MAPT, MPO, TP53*	Grade 4 neutropenic toxicity	No significant association	4
Tibaldi et al. (2008)	65 NSCLC	DDP + GEM	*ERCC1, XPD*	Grade 3-4 neutropenia, thrombocytopenia and anemia	No significant association	3
Wang et al. (2008)	139 NSCLC + SCLC	DDP + NVB/PTX/TXT/GEM/VP-16	*XPCC1*	Grade 3-4 hematologic toxicity	No significant association	4
Kim et al. (2009)	118 Epithelial ovarian cancer	DDP/CBP + PTX CBP + TXT	*ERCC1, ERCC2, XRCC1, ABCB1, GSTP1, GSTM1, GSTT1*	Grade 3-4 hematological toxicity	*GSTP1* rs1695 (A313G, Ile105Val)	5
Seo et al. (2009)	75 Gastric cancer	L-OHP + 5-FU + LV	*ERCC1, GSTT1, GSTM1, GSTP1*	Grade 3-4 neutropenia	No significant association	3
(Wu et al. 2009)	209 NSCLC	DDP/CBP + NVB/GEM/PTX/TXTOthers DDP/CBP combinations	*XPD*	Grade 3-4 hematologic toxicity, leukocytopenia, anemia and thrombocytopenia	*XPD* rs238406 (C22541A, Arg156Arg)	7
Chen et al. (2010)	95 NSCLC	DDP + GEM/NVB/TXT	*ERCC1, ABCB1(MDR1)*	Grade≥1 hematologic toxicity	No significant association	3
Giovannetti et al. (2011)	122 Pancreatic cancer	PEXG, PDXG, EC-GemCap	*ERCC1, XPD, XRCC1*	Grade 1-4 hematological toxicity, Grade 3-4 hematological toxicity	No significant association	5
Han et al. (2011)	445 NSCLC	DDP/CBP + NVB/GEM/PTX/TXTOther DDP/CBP combinations	*ABCC2(MRP2)*	Grade 3-4 hematologic toxicity, anemia, agranulocytosis, leukocytopenia and thrombocytopenia	*ABCC2/MRP2* rs3740066 (C3972T)	7
Ludovini et al. (2011)	189 NSCLC	DDP + GEM/PTX/NVB	*ERCC1, XRCC3, XPD, P53*	Grade 3-4 hematologic toxicity	No significant association	6
Zhao et al. (2012)	663 NSCLC	DDP/CBP + NVB/GEM/PTX/TXTOthers DDP/CBP combinations	*MMP-2*	Grade 3-4 hematologic toxicity, neutropenia, anemia and thrombocytopenia	*MMP-2* rs1477017, *MMP-2* rs17301608, *MMP-2* rs12934241, *MMP-2* rs243847, *MMP-2* rs243844, *MMP-2* rs11639960, *MMP-2* rs199211	8
Erčulj et al. (2012)	94 Malignant mesothelioma	DDP/CBP + GEM/PEM DDP + MMC + VCR	*XPD, ERCC1, GSTP1, GSTM1, GSTT1*	Grade 1-4 thrombocytopenia, Grade 2-4 leukopenia, anemia and neutropenia	*ERCC2/XPD* rs1799793 (Asp312Asn), *ERCC1* rs3212986 (C8092A), *GSTM1* gene deletion	6
Gu et al. (2012)	445 NSCLC	DDP/CBP + NVB/GEM/PTX/TXTOther DDP/CBP combinations	*BCL2, BAX, CASP3, CASP8, CASP10, TNFa, MIF*	Grade 3-4 hematologic toxicity	*CASP3* rs6948 (1296A>C, 3′-UTR)	8
Iwata et al. (2012)	53 Advanced carcinomas	DDP + 5-FU/GEM/TXT/VP-16/PEM/CPT-11	*OCT2, MATE1*	Grade 2-4 leukopenia and thrombocytopenia	No significant association	2
Khrunin et al. (2010)	104 Ovarian cancer	DDP + CTX	*GSTA1, GSTM1, GSTM3, GSTP1, GSTT1, ERCC1, XPD, XPCC1, TP53, CYP2E1*	Grade 3-4 neutropenia, Grade 2-4 anemia, Grade 1-4 thrombocitopenia	*XPCC1* rs25487 (Arg399Gln), *TP53* rs1042522 (C>G, Pro72Arg), *ERCC2/XPD* rs1799793 (Asp312Asn), *GSTM1* gene deletion, *GSTM3* AGG deletion	4
Qian et al. (2012)	279 (in a discovery set) and 384 (in a validation set) NSCLC	DDP/CBP + NVB/GEM/PTX/TXTOther DDP/CBP combination	*CASP8, CASP10*	Grade 3-4 hematologic toxicity, leukocytopenia, agranulocytosis, anemia, thrombocytopenia	*CASP8* rs12990906(A>G)	8
Xu et al. (2012)	204 NSCLC	DDP + GEM/VP-16/TXT/VDS	*CTR1*	Grade 3-4 neutropenia, anemia and thrombocytopenia	No significant association	5
Zhan et al. (2012)	445 NSCLC	DDP/CBP + NVB/GEM/PTX/TXTOthers DDP/CBP combinations	*Hsa-miR-196a2*	Grade 3-4 hematologic toxicity, leukocytopenia, neutropenia, thrombocytopenia and anemia	No significant association	6
Cortejoso et al. (2013)	106 Colorectal cancer	L-OHP + 5-FU + LV L-OHP + CAP	*ABCB1, XRCC1, ERCC1, ERCC2, GSTP1, GSTT1*	Grade 3-4 hematological toxicity, anemia, neutropenia, neutropenia febrile, leucopenia and thrombocytopenia	*ERCC1* rs11615 (C118T, Asn118Asn)	6
Goričar et al. (2014)	139 Malignant mesothelioma	DDP + GEM/PEM Other DDP doublets	*REV1, REV3L*	Grade 2-4 neutropenia, leukopenia, anemia and thrombocytopenia	*REV1* rs3087403(C>T), *REV1* rs3087386(A>G)	5
Lee et al. (2013)	292 Colon cancer	L-OHP + LV + 5-FU	*MTHFR, ERCC1, XPD, XRCC1, ABCC2, AGXT, GSTP1, GSTT1, GSTM1*	Grade 3-4 neutropenia, anemia, thrombocytopenia and febrile neutropenia	*MTHFR* rs1801133(C677T), *ERCC1* rs11615 (C118T, Asn118Asn), *ABCC2/MRP2* rs717620 (C-24T)	6
Li et al. (2014)	1004 NSCLC	DDP/CBP + NVB/GEM/PTX/TXTOther DDP/CBP combinations	*MTHFR*	Grade 3-4 hematologic toxicity, neutropenia, leucopenia, anemia and thrombocytopenia	*MTHFR* rs1537514(G>C), *MTHFR* rs1801133(C677T)	8
Low et al. (2013)	1171 Carcinomas	DDP/CBP-based chemotherapy	GWAS	Grade 3-4 neutropenia/leucopenia	rs4886670 near *RPL36AP45*; rs10253216 near AGR2, rs11071200 on *PRTG*	5
Peng et al. (2013)	663 NSCLC	DDP/CBP + NVB/GEM/PTX/TXTOther DDP/CBP combinations	*VCP*	Grade 3-4 hematologic toxicity, neutropenia, anemia and thrombocytopenia	*VCP* rs2074549	7
Corrigan et al. (2014)	136 NSCLC Malignant mesothelioma	DDP/CBP + PEM	*MTHFR, ERCC2*	Grade 3-4 hematologic toxicity and neutropaenia	No significant association	8
Cai et al. (2014)	663 NSCLC	DDP/CBP + NVB/GEM/PTX/VP-16/BEV	*CDC25A, CDC25B, CDC25C*	Grade 3-4 hematology toxicity	*CDC25B* rs3761218	6
Chen et al. (2014)	412 NSCLC + SCLC	DDP/CBP + GEM/PTX/NVB/VP-16/CPT-11	*WISP1*	Grade 3-4 hematologic toxicity	*WISP1* rs16904853, *WISP1* rs2929970, *WISP1* rs2977549, *WISP1* rs2977551	6
Kanazawa et al. (2014)	41 Non-squamous non-small cell lung cancer	CBP + PEM	*MTHFR*	Grade 3-4 leukopenia, neutropenia, anemia and thrombocytopenia	No significant association	4
Peng et al. (2014)	235 NSCLC	DDP + PTX/GEM/NVB/PEM	*OGG1, APE1, XPCC1*	Grade 3-4 hematologic toxicity	*XPCC1* rs25487	7
Ruzzo et al. (2014)	517 Colorectal cancer	L-OHP + 5-FU + LV L-OHP + CAP	*MTHFR, ERCC1, XRCC1, XPD, XRCC3, GST-PI, GST-T1, GST-M1, ABCC1, ABCC2*	Grade 3-4 neutropenia	No significant association	7
Shao et al. (2014)	663 NSCLC	DDP/CBP + NVB/GEM/PTX/TXTOther DDP/CBP combinations	*POLK*	Grade 3-4 hematological toxicity, anemia, agranulocytosis, leukocytopenia and thrombocytopenia	*POLK* rs3756558	6
Tan et al. (2014)	1004 NSCLC	DDP/CBP + NVB/GEM/PTX/TXTOthers DDP/CBP combinations	*MIF, JAB1, SKP1, CUL1, RBX1, NEDD8, CAND1, CD74*	Grade 3-4 hematologic toxicity, neutropenia, leucopenia, anemia and thrombocytopenia	*MIF* rs482244 (G>A), *MIF* rs4822446(A>G), *MIF* rs12485068(A>G), *CD74* rs2748249(C>A), *CD74* rs1560661(G>A)	7
Wang et al. (2014)	119 SCLC	DDP + VP-16	*MDM2, TP53*	Grade 3-4 neutropenia	*MDM2* rs2279744, *TP53* rs1042522	6
Zhao et al. (2015)	1004 NSCLC	DDP/CBP + NVB/GEM/PTX/TXTOthers DDP/CBP combinations	*TERT*	Grade 3-4 hematologic toxicity, neutropenia, anemia and thrombocytopenia	No significant association	8
Zheng et al. (2014)	444 NSCLC	DDP/CBP + GEM/VP-16/PTX/PEM Other	*TP53, MDM2*	Grade 3-4 hematologic toxicity	*MDM2* rs937282	7
Cao et al. (2016)	333 (in discovery cohort) and 876 (in validation cohort) NSCLC	DDP/CBP + GEM/PTX/TXT	GWAS	Grade 3-4 myelosuppression	rs13014982 at 2q24.3, rs9909179 at 17p12	7
Chen et al. (2016)	317 NSCLC + SCLC	DDP/CBP + GEM/PTX/NVB/VP-16/CPT-11	*ABCB1, ABCG2, AQP2, AQP9, MVP, OCT2, SIRT1, SLC2A1, TMEM205, HMGB2, RPA1, SSRP1, XPA, XRCC5*	Grade 3-4 hematologic toxicity	*XRCC5* rs1051685, *XRCC5* rs6941, *AQP2* rs10875989	6
Deng et al. (2015)	97 NSCLC	DDP + GEM/NVB/PTX/TXT	*XPCC1, GSTP1, ATP7A*	Grade 1-4 lymphopenia, leukopenia, neutropenia, thrombocytopenia and anemia	*XRCC1* rs25487(G23885A, Arg399Gln)	5
Gréen et al. (2016)	32(in discovery cohort) and 291(in validation cohort) NSCLC	CBP + GEM	Whole-Exome Sequencing	Grade 3-4 neutropenia, thrombocytopenia	rs1453542 in *OR4D6*, rs5925720 in *DDX53*	7
Huang et al. (2015)	286 Cervical cancer	DDP/CBP + taxanes/CPT-11	*GWAS*	Grade 2-4 neutropenia	32 variants	6
Kalikaki et al. (2015)	107 NSCLC	DDP/CBP + PTX/GEMDDP + TXT/NVB	*ERCC1, XPD, XRCC1*	Grade 3-4 hematologic toxicity	No significant association	4
Lambrechts et al. (2015)	290 Ovarian cancer	CBP + PTX CBP mono-therapy	*ABCB1, ABCC1, ABCC2, ABCG2, TP53, GSTP1, ERCC1, ERCC2*	Grade 3-4 anemia, thrombocytopenia and febrile neutropenia, Grade 4 neutropenia	*ABCB1/MDR1* rs1128503 (C1236T), *ABCC2/MRP2* rs12762549 (*+9383C>G), *ERCC1* rs11615 (C118T, Asn118Asn)	8
Qian et al. (2015)	663 NSCLC	DDP/CBP + NVB/GEM/PTX/TXT Other combinations	*MDM2*	Grade 3-4 hematologic toxicity	*MDM2* rs1470383(G>A)	7
Ye et al. (2015)	663 NSCLC	DDP/CBP + NVB/GEM/PTX/TXTOthers DDP/CBP combinations	*REV3, REV7*	Grade 3-4 hematological toxicity, anemia, agranulocytosis, leukocytopenia and thrombocytopenia	*REV3* rs240966, *REV3* rs4945880(G>A), *REV3* rs465646(G>A), *REV7* rs2233025(G>A)	7
Yin et al. (2015)	325 NSCLC	DDP/CBP + GEM/VP-16/PTX/PEM Other	*eIF3a*	Grade 1-4 neutropenia, anemia and thrombocytopenia	*eIF3a* rs1409314, *eIF3a* rs4752219, *eIF3a* rs4752220, *eIF3a* rs7091672	5
Chu et al. (2016)	1021 NSCLC	DDP/CBP + NVB/GEM/PTX/TXTOther DDP/CBP combinations	*Rad18*	Grade 3-4 hematological toxicity, anemia, agranulocytosis, leukocytopenia and thrombocytopenia	No association in the whole population. Significant association in subgroup: *RAD18* rs586014(A>G), *RAD18* rs654448(G>A), *RAD18* rs9880051(G>A), *RAD18* rs6763823(G>A)	6
Fang et al. (2017)	408 NSCLC + SCLC	DDP/CBP + NVB/GEM/TXT/VP-16	*miR-605, miR-146a, miR-149, miR-196a-2, miR-27a, miR-499, miR-30c-1, miR-5197*	Grade 3-4 haematologic toxicity	No association in the whole population. Significant association in subgroup: *miR-5197* rs2042253	7
Guo et al. (2016)	292 Lung adenocarcinoma	DDP/CBP combinations	*p53, MDM2*	Grade 3-4 hematologic toxicity	*MDM2* rs2279744 (309T>G)	5
Hu et al. (2016)	467 NSCLC + SCLC	DDP/CBP + GEM/VP-16/PEM/TXT/PTX/CPT-11/NVB	*CASC8*	Grade 3-4 hematologic toxicity	No significant association in overall subjects. Significant association in subgroup: *CASC8* rs10505477	6
Jia et al. (2016)	345 (in discovery group) and 344 (in replication group) NSCLC	DDP/CBP + PEM/TXT/PTX/GEM	*GADD45A, GADD45B, GADD45G, MAP2K7, MAP2K4, MAP3K4, MAPK8, MAPK9, MAPK14*	Grade 2-4 leukopenia, neutropenia, thrombocytopenia and anemia	*GADD45B* rs2024144(C>T), *GADD45B* rs2024144(C>T), *GADD45B* rs2024144(C>T)	6
Kumpiro et al. (2016)	32 NSCLC	CBP + GEM	*CTR1*	Grade 1-4 anemia, thrombocytopenia and neutropenia	No significant association	3
Qian et al. (2016)	403 NSCLC	DDP/CBP + GEM/PEM/PTX/TXT/NVB	*OCT2, ABCB1, ABCC2(MRP2), MATE1*	Grade 3-4 hematologic toxicity	*OCT2* rs316019 (808G/T, p.270Ala > Ser), *MATE1* rs2289669	6
Song et al. (2017)	1004 NSCLC	DDP/CBP + NVB/GEM/PTX/TXTOthers DDP/CBP combinations	*XPC, RAD23B, ERCC2, GTF2H1, XPA, ERCC5, ERCC1, ERCC4, ERCC8, ERCC, DDB2, LIG1, CDK7, CCNH, MNAT1, RPA1, RPA2, RFC1, RFC2, POLD1, POLD2, POLD3, POLD4, POLE, POLE2, GTF2H3, GTF2H4*	Grade 3-4 anemia, neutropenia and trombocytopenia	No SNPs satisfied the significant level of bonferroni correction *GTF2H1* rs4150558, *POLD3* rs10857, *POLD3* rs6592576, *RPA1* rs12727, *POLD1* rs3219281, *POLD1* rs3219341, *POLD1* rs1726801	5
Wang et al. (2016)	1004 NSCLC	DDP/CBP + NVB/GEM/PTX/TXTOthers DDP/CBP combinations	*RICTOR*	Grade 3-4 hematologic toxicity, anemia, neutropenia and thrombocytopenia	*RICTOR* rs7703002, *RICTOR* rs4321771	8
Xu et al. (2016)	272 female patients NSCLC	DDP/CBP + NVB/GEM/PTX/TXTOthers DDP/CBP combinations	*CHEK2*	Grade 3-4 hematological toxicity and leukotoxicity	No significant association	6
Yin et al. (2016)	190 (in Derivation cohort) and 200 (in Derivation cohort) NSCLC	DDP/CBP + GEM/PEM/PTX/TXT/NVB/VP-16	416 SNPs in 185 genes	Grade 3-4 hematological toxicity	The hematological toxicity prediction model achieved a sensitivity of 0.89 and a specificity of 0.39 with the ROC AUC of 0.76	5
Zou et al. (2016)	317 NSCLC + SCLC	DDP/CBP + GEM/PTX/NVB/VP-16/CPT-11	*HSPA4, HSPB1, HSPE1, RAC1, RhoA*	Grade 3-4 hematologic toxicity	*RAC1* rs836554, *RAC1* rs4720672	6
Gong et al. (2017)	467 NSCLC + SCLC	DDP/CBP + PEM/GEM/PTX/TXT/VP-16Other DDP/CBP-based chemotherapy (CPT-11 + DDP/CBP, NVB + DDP/CBP)	*HOTTIP, HOTAIR, H19, ANRIL, CCAT2, MALAT1, MEG3, POLR2E*	Grade 3-4 hematological toxicity	No association in the whole population significant association in subgroup	6
Liu et al. (2017a)	555 Lung adenocarcinoma	DDP/CBP + PTX/TXT/NVB/VP-16/BEV	*CASP8*	Grade 3-4 neutropenia, thrombocytopenia and anemia	*CASP8* rs7608692(G>A)	6
Liu et al. (2017b)	220 NSCLC + SCLC	DDP/CBP + GEM/PEM/PTX/TXT/NVB	*MLH1, MSH2, MSH3, MSH4, MSH5, MSH6*	Grade 3-4 hematologic toxicity	*MSH3* rs6151627, MSH3 rs6151670, MSH3 rs7709909, MSH5 rs805304	6
Zheng et al. (2017)	437 (in the discovery cohort) and 781 (in the validation cohort) NSCLC	DDP/CBP + NVB/GEM/PTX/TXT/PEM	54 gene	Grade 3-4 hematologic toxicity, leukocytopenia, neutropenia, thrombocytopenia and anemia	*ERCC1* rs3212986, *ERCC1* rs11615, *RRM1* rs12806698, *XPC* rs2228001, *XPC* rs2228000, *XPF* rs1799801, *XPG* rs1047768, *XPG* rs17655, *APE1* rs1130409, *XRCC1* rs25487, *MDM2* rs2279744, *RAD51* rs1801320, *RAD51* rs12593359	8
Björn et al. (2020a)	215 in the discovery cohort) and validated in an independent genome-wide association study NSCLC	CBP + GEM	whole-exome sequence	Grade 3-4 thrombocytopenia	These analyses identified 130 SNVs/INDELs and 25 genes associated with thrombocytopenia (*p*-value <0.002). Twenty-three SNVs were validated in an independent genome-wide association study (GWAS)	7
De Troia et al. (2019)	82 NSCLC + SCLC	DDP/CBP + VP-16/NVBDDP + GEM/PEM/TXT DDP monotherapy	*ABCB1, ABCC2, GSTP1*	Grade 3-4 hematological toxicity	*ABCB1/MDR1* rs1045642 (C3435T): decreased risk of grade 3-4 hematological toxicity	5
Li et al. (2018)	427 NSCLC	DDP/CBP + GEM/PEM/TXT/NVB/PTX	*ATP7A, ATP7B*	Grade 3-4 hematological toxicity	No significant association	6
Sun et al. (2018)	1004 NSCLC	DDP/CBP + NVB/GEM/PTX/TXTOthers DDP/CBP combinations	*SCL31A1*	Grade 3-4 hematological toxicity, anemia, neutropenia, leukocytopenia and thrombocytopenia	*SLC31A1* rs4979223, *SLC31A1* rs4978536, *SLC31A1* rs10817464, *SLC31A1* rs10759637	8
Yoshihama et al. (2018)	320 Ovarian fallopian tube, peritoneal, uterine, or cervical cancer	CBP + PTX	*37 transporters, 30 cytochrome P450 (CYP) enzymes, 10 uridine diphosphate UDPglucuronosyltransferases (UGT), five flavin-containing monooxygenases (FMO), four glutathione S-transferases (GST), four sulfotransferases (SULT), and 10 additional genes*	Severe hematotoxicity (including neutropenia G4, thrombocytopenia ≥ G3, and anemia ≥ G3)	*GSTP1* rs1695	5
Gong et al. (2019)	467 NSCLC + SCLC	DDP/CBP + PEM/GEM/PTX/TXT/VP-16Other DDP/CBP-based chemotherapy (CPT-11 + DDP/CBP, NVB + DDP/CBP)	*STAT3*	Grade 3-4 hematological toxicity	*STAT3* rs4796793	6
Lavanderos et al. (2019)	119 Testicular cancer	DDP + BLM + VP-16	*GSTM1, GSTP1, GSTT1, UGT1A1, BLMH, ERCC1, ERCC2, MDR1*	Grade 3-4 anemia, neutropenia, leukopenia, thrombocytopenia, lymphocytopenia and febrile neutropenia	*ERCC2/XPD* rs238406 (C22541A, Arg156Arg), *ERCC1* rs11615 (C118T, Asn118Asn)	5
Liblab et al. (2020)	52 Ovarian cancer (Epithelial ovarian cancer)	CBP + PTX CBP mono-therapy	*ERCC1, XRCC1, GSTP1*	Grade 2-4 anemia	*GSTP1* rs1695 (A313G, Ile105Val)	2
Senk et al. (2019)	194 Malignant mesothelioma	DDP + GEM/PEM	*AQP1*	Grade 2-4 anemia, leukopenia and neutropenia, Grade 1-4 thrombocytopenia	*AQP1* rs28362731(G>A), *AQP1* rs1049305(G>C)	5
Björn et al. (2020b)	96 (split up into 80% training and 20% validation) NSCLC	CBP + GEM	Whole-genome sequencing	Grade 3-4 neutropenia, leukopenia and thrombocytopenia	4594, 5019, and 5066 autosomal SNVs/INDELs	6
Bushra et al. (2020)	285 NSCLC	DDP/CBP + GEM/NVB/PTX/TXT	*GSTP1, XRCC1, XPC, ERCC1*	Grade 3-4 anemia, neutropenia, leukopenia and thrombocytopenia	*GSTP1* rs1695 (A313G, Ile105Val), *XRCC1* rs25487(G23885A, Arg399Gln), *XPC* rs2228001(A>C, Lys939Gln)	5
Ferracini et al. (2021)	112 Ovarian cancer (Epithelial ovarian cancer)	CBP + PTX CBP mono-therapy	*GSTP1, ABCB1*	Grade 3-4 anemia, neutropenia, Grade 1-4 thrombocytopenia	*GSTP1* rs1695 (A313G, Ile105Val), *ABCB1/MDR1* rs1128503 (C1236T)	7
Nomura et al. (2020)	158 Esophageal cancer	DDP + TXT + 5-FU	*ABCB1, ABCC2, ABCG2, GSTM1, GSTT1, GSTP1*	Grade 3-4 neutropenia	*ABCB1/MDR1* rs1045642 (C3435T), *ABCC2/MRP2* rs12762549 (*+9383C>G)	6
Svedberg et al. (2020)	215 (in discovery cohort) and 144 (in validation cohort) NSCLC	CBP + GEM	whole-exome sequencing	Grade 3-4 neutropenia and leucopenia	50 and 111 SNVs, and 12 and 20 genes This study created wGRS models for predicting the risk of chemotherapy-induced hematological toxicity	7
Nairuz et al. (2021)	180 Lung cancer	DDP/CBP + VP-16/PTX/TXTCBP + GEM/ADM	*XPD, TP53*	Grade 3-4 neutropenia, leucopenia, anemia and thrombocytopenia	No significant association	2
Walia et al. (2021)	317 NSCLC + SCLC	DDP/CBP + PEM/CPT-11/TXT/PTX/GEM	*GSTP1*	Grade 3-4 anemia, Grade 1-4 anemia, Grade 2-4 anemia, Grade 1-4 leukopenia, Grade 2-4 leukopenia	*GSTP1* rs1695	7
Walia et al. (2022)	123 Lung adenocarcinoma cancer	DDP/CBP + PEM	*MTHFR*	Grade 1-3 neutropenia, Grade 2-3 neutropenia	*MTHFR* rs1801133	7
Wang et al. (2021)	1004 NSCLC	DDP/CBP + NVB/GEM/PTX/TXTOthers DDP/CBP combinations	*ABCG2*	Grade 3-4 hematologic toxicity, anemia, neutropenia and thrombocytopenia	*ABCG2* rs12505410, *ABCG2* rs1871744, *ABCG2* rs2231138	7
Zheng et al. (2021)	437 NSCLC	DDP/CBP + NVB/GEM/PTX/TXT/PEM	*EPO*	Grade 3-4 hematologic toxicity, leukocytopenia, neutropenia, thrombocytopenia and anemia	*EPO* rs1617640	6

Abbreviations: ADM, doxorubicin; BEV, bevacizumab; BLM, bleomycin; CAP, capecitabine; CBP, carboplatin; CI, confidence interval; CPT-11, irinotecan; CTCAE, common terminology criteria for adverse events; CTX, cyclophosphamide; DDP, cisplatin; EC-GemCap, epirubicin cisplatin (intra-arterial infusion)-gemcitabine capecitabine; GEM, gemcitabine; L-OHP, oxaliplatin; LV, leucovorin; MMC, mitomycin C; NSCLC, non-small cell lung cancer; NVB, navelbine; OR, odds ratios; PDXG, cisplatin, docetaxel capecitabine, gemcitabine; PEM, pemetrexed; PEXG, cisplatin, epirubicin, capecitabine, gemcitabine; PTX, paclitaxel; SCLC, small cell lung cancer; TXT, docetaxel; VCR, vincristine; VDS, vindesine; VP-16, etoposide; 5-FU, fluorouracil.

59 studies involved a single ethnic group [42 Chinese or Han Chinese, 5 Caucasian (Marsh et al., 2007; Tibaldi et al., 2008; Giovannetti et al., 2011; Ludovini et al., 2011; Lambrechts et al., 2015), 4 Japanese (Iwata et al., 2012; Low et al., 2013; Yoshihama et al., 2018; Nomura et al., 2020), 3 Indian (Ruzzo et al., 2014; Walia et al., 2021; Walia et al., 2022), 2 Korean (Kim et al., 2009; Lee et al., 2013), 1 Thai (Kumpiro et al., 2016), 1 Bangladeshi (Nairuz et al., 2021) and 1 eastern Slavonic origin (Khrunin et al., 2010)]. Three studies are mixed ancestry (Corrigan et al., 2014; Lambrechts et al., 2015; Ferracini et al., 2021). 21 studies did not explain how ancestry was determined.

Individual study sizes ranged from 32 to 1171 patients. 12 studies had fewer than 100 subjects. 10 studies involved discovery and replication sets (Marsh et al., 2007; Qian et al., 2012; Cao et al., 2016; Gréen et al., 2016; Jia et al., 2016; Yin et al., 2016; Zheng et al., 2017).

The studied cancer types were: lung cancer (n = 63), ovarian cancer (n = 6) (Marsh et al., 2007; Kim et al., 2009; Khrunin et al., 2010; Lambrechts et al., 2015; Liblab et al., 2020; Ferracini et al., 2021), malignant mesothelioma (n = 3) (Erčulj et al., 2012; Goričar et al., 2014; Senk et al., 2019), colorectal cancer (n = 3) (Cortejoso et al., 2013; Ruzzo et al., 2014), one study each evaluated colon cancer (Lee et al., 2013), gastric cancer (Seo et al., 2009), testicular cancer (Lee et al., 2013), esophageal cancer (Nomura et al., 2020), cervical cancer (Huang et al., 2015), pancreatic cancer (Giovannetti et al., 2011), four studies included various cancer types (Corrigan et al., 2014; Yoshihama et al., 2018) or did not mention the cancer type (Low et al., 2013).

64 studies used mixed combination chemotherapy: cisplatin/carboplatin-based chemotherapy (n = 51), cisplatin-based chemotherapy (n = 10), carboplatin-based chemotherapy (n = 3), 19 study uses single platinum-based chemotherapy. Platinum dosage and cycles varied according the tumor type: mostly cisplatin 75 mg/m^2^ or carboplatin AUC 5, both administered on day 1 every 3 weeks. The dose of oxaliplatin was 85 mg/m^2^ or 130 mg/m^2^ 29 studies did not mention the dose of platinum agents.

47 studies analyzed the whole hematological toxicities. 56 studies analyzed detailed hematologic toxicity (leukopenia, neutropenia, thrombocytopenia or anemia). 20 studies analyzed both of them. Most studies (n = 80) use National Cancer Institute-Common Terminology Criteria for Adverse Events (NCI-CTCAE), three studies use WHO criteria (Isla et al., 2004; Chen et al., 2010; Huang et al., 2015).

The most used end-point of toxicity was the occurrence of grade ≥3 toxicity (n = 66). Other studies used a cut-off value of grade ≥1 (n = 12), grade ≥2 (n = 11), or grade 4 (n = 2) (Han et al., 2007; Marsh et al., 2007). One study described severe hematological toxicity including neutropenia G4, thrombocytopenia ≥ G3, and anemia ≥ G3 (Yoshihama et al., 2018). Eight studies analyzed more than one end-point of grading (Khrunin et al., 2010; Giovannetti et al., 2011; Erčulj et al., 2012; Lambrechts et al., 2015; Senk et al., 2019; Ferracini et al., 2021; Walia et al., 2021; Walia et al., 2022).

Among the 83 included studies, 28 (44.4%) were considered high quality, 46 (55.4%) moderate quality, and 9 (10.8%) were of low quality (Supplementary Table S6), the main reason for low study quality was the absence of calculation of sample size (n = 82), no consideration of population stratification (n = 80) and lack of statistical correction for multiple testing (n = 53).

### 3.3 Genetic associations investigated in platinum-induced hematological toxicity

51 variants were analyzed in more than one study (Tables 2, 3; Supplementary Table S7).

**TABLE 2:** Genetic polymorphisms investigated more than twice for association with platinum-induced hematological toxicity.

Pathway	Gene	SNP/deletion	Total (n)	With association		Without association
				Increased risk	Decreased risk	
Transporter	*SCL31A1 (CTR1)*	rs10981699 (C>T)	2	0	0	2 (Han et al., 2007; Giuliano, 2012)
Transporter	*SCL31A1 (CTR1)*	rs10817465 (C>G)	2	0	0	2 (Han et al., 2007; Giuliano, 2012)
Transporter	*SCL31A1 (CTR1)*	rs12686377 (C>A)	2	0	0	2 (Giuliano, 2012; Lee et al., 2013)
Transporter	*ABCC2(MRP2)*	rs717620 (C-24T)	6	0	1 (Kim et al., 2009)	5 (Isla et al., 2004; Cara and M Eileen, 2006; Ludovini et al., 2011; Xu et al., 2012; Sun et al., 2018)
Transporter	*ABCC2(MRP2)*	rs3740066 (C3972T, Ile1324Ile)	5	1 (Sun et al., 2018)	0	4 (Isla et al., 2004; Kim et al., 2009; Xu et al., 2012; Nomura et al., 2020)
Transporter	*ABCC2(MRP2)*	rs2273697 (G1249A, Val417Ile)	4	0	0	4 (Isla et al., 2004; Ludovini et al., 2011; Xu et al., 2012; Sun et al., 2018)
Transporter	*ABCC2(MRP2)*	rs12762549 (*+9383C>G)	2	1 (Iwata et al., 2012)	1 (Giovannetti et al., 2011)	0
Transporter	*ABCC2(MRP2)*	rs2073337 (c.1668 + 148A>G)	2	0	0	2 (Giovannetti et al., 2011; Ludovini et al., 2011)
Transporter	*ABCB1(MDR1)*	rs1045642(C3435T, Ile1145Ile)	12	1 (Iwata et al., 2012)	1 (Cara and M Eileen, 2006)	10 (Isla et al., 2004; Seo et al., 2009; Chen et al., 2010; Khrunin et al., 2010; Giovannetti et al., 2011; Han et al., 2011; Ludovini et al., 2011; Xu et al., 2012; Senk et al., 2019; Walia et al., 2022)
Transporter	*ABCB1(MDR1)*	rs2032582(G2677T/A, Ala893Ser)	7	1 (Isla et al., 2004)	0	6 (Seo et al., 2009; Khrunin et al., 2010; Ludovini et al., 2011; Iwata et al., 2012; Senk et al., 2019; Walia et al., 2022)
Transporter	*ABCB1(MDR1)*	rs1128503(C1236T, Gly412Gly)	6	2 (Khrunin et al., 2010; Giovannetti et al., 2011)	0	4 (Isla et al., 2004; Ludovini et al., 2011; Iwata et al., 2012; Senk et al., 2019)
Transporter	*ABCG2*	rs2231142 (421C>A, Gln141Lys)	5	0	0	5 (Isla et al., 2004; Meyer zu Schwabedissen et al., 2005; Giovannetti et al., 2011; Ludovini et al., 2011; Iwata et al., 2012)
Transporter	*ABCG2*	rs2231137 (34G>A, Val12Met)	3	0	0	3 (Isla et al., 2004; Meyer zu Schwabedissen et al., 2005; Iwata et al., 2012)
Transporter	*OCT2*	rs316019 (808G/T, p.270Ala > Ser)	2	0	1 (Xu et al., 2012)	1 (Tibaldi et al., 2008)
Transporter	*MATE1*	rs2289669 (G/A)	2	1 (Xu et al., 2012)	0	1 (Tibaldi et al., 2008)
Metabolism	*GSTP1*	rs1695 (A313G, Ile105Val)	18	2 (Ruzzo et al., 2014; Zheng et al., 2017)	4 (Moriya et al., 2002; Khrunin et al., 2010; Björn et al., 2020b; Walia et al., 2022)	12 (Kim et al., 2001; Cara and M Eileen, 2006; Kim et al., 2009; Giovannetti et al., 2011; Han et al., 2011; Ludovini et al., 2011; Iwata et al., 2012; Cortejoso et al., 2013; Senk et al., 2019; Liblab et al., 2020; Nomura et al., 2020; Nairuz et al., 2021)
Metabolism	*GSTP1*	rs1138272 (c.341 C>T, Ala114Val)	4	0	0	4 (Giovannetti et al., 2011; Ludovini et al., 2011; Liblab et al., 2020; Nairuz et al., 2021)
Metabolism	*GSTT1*	gene deletion	9	0	0	9 (Kim et al., 2009; Han et al., 2011; Iwata et al., 2012; Cortejoso et al., 2013; Senk et al., 2019; Liblab et al., 2020; Nomura et al., 2020; Nairuz et al., 2021; Walia et al., 2022)
Metabolism	*GSTM1*	gene deletion	8	0	2 (Liblab et al., 2020; Nairuz et al., 2021)	6 (Kim et al., 2009; Han et al., 2011; Iwata et al., 2012; Cortejoso et al., 2013; Nomura et al., 2020; Walia et al., 2022)
NER	*ERCC1*	rs11615 (C118T, Asn118Asn)	19	4 (Kim et al., 2009; Giovannetti et al., 2011; Han et al., 2011; Yin et al., 2016)	1 (Senk et al., 2019)	14 (Moriya et al., 2002; Marsh et al., 2007; Seo et al., 2009; Chen et al., 2010; Ludovini et al., 2011; Cortejoso et al., 2013; Zheng et al., 2014; Lambrechts et al., 2015; Qian et al., 2016; Yoshihama et al., 2018; Liblab et al., 2020; Nomura et al., 2020; Nairuz et al., 2021; Walia et al., 2022)
NER	*ERCC1*	rs3212986 (C8092A)	12	0	2 (Yin et al., 2016; Liblab et al., 2020)	10 (Kim et al., 2009; Han et al., 2011; Ludovini et al., 2011; Cortejoso et al., 2013; Zheng et al., 2014; Qian et al., 2015; Qian et al., 2016; Zheng et al., 2017; Nairuz et al., 2021; Walia et al., 2022)
NER	*ERCC1*	rs3212961(17677G>T)	3	0	0	3 (Giovannetti et al., 2011; Ludovini et al., 2011; Qian et al., 2016)
NER	*ERCC2/XPD*	rs13181(A>C, Lys751Gln)	17	0	1 (Chen et al., 2010)	16 (Marsh et al., 2007; Kim et al., 2009; Han et al., 2011; Ludovini et al., 2011; Zheng et al., 2014; Lambrechts et al., 2015; Kumpiro et al., 2016; Qian et al., 2016; Yin et al., 2016; Yoshihama et al., 2018; Senk et al., 2019; Liblab et al., 2020; Nomura et al., 2020; Ferracini et al., 2021; Nairuz et al., 2021; Walia et al., 2022)
NER	*ERCC2/XPD*	rs1799793 (G23591A, Asp312Asn)	13	1 (Nairuz et al., 2021)	1 (Liblab et al., 2020)	11 (Hoffmeyer et al., 2000; Marsh et al., 2007; Kim et al., 2009; Chen et al., 2010; Giovannetti et al., 2011; Han et al., 2011; Zheng et al., 2014; Qian et al., 2016; Yin et al., 2016; Yoshihama et al., 2018; Nomura et al., 2020)
NER	*ERCC2/XPD*	rs238406 (C22541A, Arg156Arg)	5	2 (Hoffmeyer et al., 2000; Han et al., 2011)	0	3 (Kim et al., 2009; Qian et al., 2016; Yin et al., 2016)
NER	*ERCC2/XPD*	rs1052555 (G>A, Asp711Asp)	3	0	0	3 (Hoffmeyer et al., 2000; Qian et al., 2016; Yin et al., 2016)
NER	*ERCC4 (XPF)*	rs1799801(T>C, Ser835Ser)	2	1 (Yin et al., 2016)	0	1 (Qian et al., 2016)
NER	*XPC*	rs2228001(A>C, Lys939Gln)	3	2 (Moriya et al., 2002; Yin et al., 2016)	0	1 (Qian et al., 2016)
NER	*XPG/ERCC5*	rs1047768 (T>C, His46His)	2	1 (Yin et al., 2016)	0	1 (Qian et al., 2016)
NER	*XPG/ERCC5*	rs17655 (G>C, His1104Asp)	2	0 (Yin et al., 2016)	0	2 (Qian et al., 2016)
NER	*CCNH*	rs2230641 (A>G, Val270Ala)	2	0	0	2 (Qian et al., 2016; Yin et al., 2016)
NER	*XPA*	rs1800975 (T>C)	2	0	0	2 (Qian et al., 2016; Yin et al., 2016)
NER	*RPA1*	rs12727 (G>C)	2	0	0	2 (Xu et al., 1998; Qian et al., 2016)
NER	*RPA1*	rs17734 (C>T)	2	0	0	2 (Xu et al., 1998; Qian et al., 2016)
BER	*XPCC1*	rs25487 (G23885A, Arg399Gln)	14	3 (Moriya et al., 2002; Yin et al., 2016; De Troia et al., 2019)	2 (Kim et al., 2001; Nairuz et al., 2021)	9 (Bosch et al., 2006; Kim et al., 2009; Ludovini et al., 2011; Zheng et al., 2014; Zheng et al., 2017; Yoshihama et al., 2018; Senk et al., 2019; Nomura et al., 2020; Walia et al., 2022)
BER	*XPCC1*	rs25489 (G23098A, Arg280His)	3	0	0	3 (Kim et al., 2009; Yin et al., 2016; Nairuz et al., 2021)
BER	*XPCC1*	rs1799782 (C21935T, Arg194Trp)	4	0	0	4 (Bosch et al., 2006; Kim et al., 2009; Nairuz et al., 2021; Walia et al., 2022)
BER	*APE1*	rs1130409 (T>G, Asp148Glu)	2	0	1 (Yin et al., 2016)	1 (De Troia et al., 2019)
BER	*OGG1*	rs1052133 (C>G, Ser326Cys)	2	0	0	2 (Yin et al., 2016; De Troia et al., 2019)
DSB	*XRCC3*	rs861539 (C>T, Thr241Met)	3	0	0	3 (Lambrechts et al., 2015; Yin et al., 2016; Nomura et al., 2020)
TLS	*REV3L*	rs462779 (G>A, Thr1224Ile)	2	0	0	2 (Erčulj et al., 2012; Yin et al., 2016)
TLS	*REV3L*	rs465646 (G>A)	3	1	0	2 (Erčulj et al., 2012; Yin et al., 2016)
TLS	*REV7*	rs746218 (G>A)	2	0	0	2 (Ding-Wu et al., 2012; Yin et al., 2016)
TLS	*REV7*	rs2233006 (T>A)	2	0	0	2 (Ding-Wu et al., 2012; Yin et al., 2016)
TLS	*REV1*	rs3087386 (A>G, Phe257Ser)	2	0	1 (Erčulj et al., 2012)	1 (Yin et al., 2016)
TLS	*Rad18*	rs373572 (C>T, Arg302Gln)	2	0	0	2 (Yin et al., 2016; Wang et al., 2021)
DNA synthesis	*MTHFR*	rs1801131 (A1298C, Glu429Ala)	6	0	0	6 (Kim et al., 2009; Zolk et al., 2009; Zolk, 2012; Nomura et al., 2020; Ferracini et al., 2021; Walia et al., 2021)
DNA synthesis	*MTHFR*	rs1801133 (C677T, Ala222Val)	6	2 (Kim et al., 2009; Walia et al., 2021)	1 (Zolk, 2012)	3 (Zolk et al., 2009; Nomura et al., 2020; Ferracini et al., 2021)
DNA synthesis	*RRM1*	rs12806698 (−37C/A)	2	1 (Yin et al., 2016)	1 (Chen et al., 2010)	0
Apoptosis	*MDM2*	rs2279744 (309T>G)	4	1 (Moyer et al., 2008)	2 (Yin et al., 2016; R et al., 2000)	1 (Clarissa Ribeiro Reily et al., 2018)
Apoptosis	*TP53*	rs1042522 (C>G, Pro72Arg)	9	2 (Nairuz et al., 2021; R et al., 2000)	0	7 (Moyer et al., 2008; Giovannetti et al., 2011; Ludovini et al., 2011; Lambrechts et al., 2015; Kumpiro et al., 2016; Yin et al., 2016; Clarissa Ribeiro Reily et al., 2018)

Abbreviations: BER, base excision repair; DSB, double-strand break repair; MMR, mismatch repair; NER, nucleotide excision repair; TLS, translesion DNA, synthesis.

**TABLE 3:** Summary of positive associations in genetic polymorphisms that investigated more than twice.

Study	Sample size, cancer	Treatment	Association	Trend
*ABCC2(MRP2)* rs717620 (C-24T)				
Lee et al. (2013)	292 Colon cancer	L-OHP + LV + 5-FU	Lower incident rate of grade 3-4 thrombocytopenia [5.6% (9 out of 160 patients with CC) vs. 0.8% (1 out of 124 with CT or TT), *p* = 0.047]	Decrease
*ABCC2(MRP2)* rs3740066 (C3972T, Ile1324Ile)				
Han et al. (2011)	445 NSCLC	DDP/CBP + NVB/GEM/PTX/TXTOther DDP/CBP combinations	Increased risk of grade 3-4 thrombocytopenia (CT + TT vs. CC OR = 2.43; 95% CI: 1.06–5.56; *p* = 0.034)	Increase
*ABCC2(MRP2)* rs12762549 (*+9383C>G)				
Nomura et al. (2020)	158 Esophageal cancer	DDP + TXT + 5-FU	Increased risk of grade 3-4 neutropenia (GG vs. GC + CC OR = 2.342; 95% CI: 1.108–4.948; *p* = 0.026)	Increase
Lambrechts et al. (2015)	290 Ovarian cancer	CBP + PTX CBP mono-therapy	Decreased risk of grade 3-4 anemia in additive model (OR = 0.51; 95% CI: 0.33–0.81; *p* = 0.004)	Decrease
*ABCB1(MDR1)* rs1045642(C3435T, Ile1145Ile)				
Nomura et al. (2020)	158 Esophageal cancer	DDP + TXT + 5-FU	Increased risk of grade 3-4 neutropenia (CT + TT vs. CC OR = 2.191; 95% CI: 1.087–4.417; *p* = 0.028)	Increase
De Troia et al. (2019)	82 Lung cancer	DDP/CBP + VP-16/NVBDDP + GEM/PEM/TXT DDP monotherapy	Decreased risk of grade 3-4 hematological toxicity (CT vs. CC OR = 0.18; 95% CI: 0.05–0.65; *p* = 0.01, CT + TT vs. CC OR = 0.24; 95% CI: 0.07–0.75; *p* = 0.01)	Decrease
*ABCB1(MDR1)* rs2032582(G2677T/A, Ala893Ser)				
Han et al. (2007)	107 NSCLC	DDP + CPT-11	Associated with grade 4 neutropenia (the incident rate of grade 4 neutropenia for GG, GT/GA and TT/TA/AA were 34.6%, 53.8%, and 11.5%, *p* = 0.030)	Increase
*ABCB1(MDR1)* rs1128503(C1236T, Gly412Gly)				
Lambrechts et al. (2015)	290 Ovarian cancer	CBP + PTX CBP mono-therapy	Increased risk of grade 3-4 anemia in additive model (OR = 1.71; 95% CI: 1.07–2.71; *p* = 0.023)	Increase
Ferracini et al. (2021)	112 Ovarian cancer (Epithelial ovarian cancer)	CBP + PTX CBP mono-therapy	Increased risk of grade 1-4 thrombocytopenia (TT vs. CC OR = 3.63; 95% CI: 0.98–13.47; *p* = 0.05, TT vs. CT + CC OR = 3.50; 95% CI: 1.12–10.97; *p* = 0.03)	Increase
*OCT2* rs316019 (808G/T, p.270Ala > Ser)				
Qian et al. (2016)	403 NSCLC	DDP/CBP + GEM/PEM/PTX/TXT/NVB	Decreased risk of grade 3-4 hematological toxicity in additive model (OR = 0.58; 95% CI: 0.34–0.97; *p* = 0.039)	Decrease
*MATE1* rs2289669 (G/A)				
Qian et al. (2016)	403 NSCLC	DDP/CBP + GEM/PEM/PTX/TXT/NVB	Increased risk of grade 3-4 hematological toxicity in recessive model (OR = 1.92; 95% CI: 1.13–3.25; *p* = 0.016)	Increase
*GSTP1* rs1695 (A313G, Ile105Val)				
Ferracini et al. (2021)	112 Ovarian cancer (Epithelial ovarian cancer)	CBP + PTX CBP mono-therapy	Decreased risk of grade 3-4 anemia (AG vs. AA OR = 0.16; 95% CI: 0.03–0.84; *p* = 0.03, AG + GG vs. AA OR = 0.17; 95% CI: 0.04–0.69; *p* = 0.01), grade 3-4 thrombocytopenia (AG vs. AA OR = 0.32; 95% CI: 0.12–0.82; *p* = 0.01, GG vs. AA OR = 0.11; 95% CI: 0.02–0.59; *p* < 0.01, AG + GG vs. AA OR = 0.27; 95% CI: 0.12–0.64; *p* < 0.01, GG vs. AA+ AG OR = 0.18; 95% CI: 0.03–0.85; *p* = 0.03)	Decrease
Kim et al. (2009)	118 Ovarian cancer (Epithelial ovarian cancer)	DDP/CBP + PTX CBP + TXT	Associated with grade 3-4 hematological toxicity (the incident rate of grade 3-4 hematology toxicity for AG/GG and AA were 54.2% and 78.7%, *p* = 0.015)	Decrease
Bushra et al. (2020)	285 NSCLC	DDP/CBP + GEM/NVB/PTX/TXT	Decreased risk of grade 3-4 anemia (GG vs. AA OR = 0.29; 95% CI: 0.10–0.87; *p* = 0.027) and grade 3-4 neutropenia (GG vs. AA OR = 0.31; 95% CI: 0.10–0.96; *p* = 0.043)	Decrease
Yoshihama et al. (2018)	320 Ovarian fallopian tube, peritoneal, uterine, or cervical cancer	CBP + PTX	Decreased risk of severe hematotoxicity (including neutropenia G4, thrombocytopenia ≥ G3,and anemia ≥ G3) (A allele vs. G allele OR = 5.71; 95% CI: 1.77–18.44; *p* = 0.00034)	Decrease
Walia et al. (2021)	317 Lung cancer	DDP/CBP + PEM/CPT-11/TXT/PTX/GEM	Increased risk of grade 3-4 anemia (AG vs. AA OR = 2.12; 95% CI: 0.97–4.62; *p* = 0.04) and grade 2-4 leukopenia (GG vs. AA OR = 2.41; 95% CI: 1.39–4.18; *p* = 0.001)	Increase
Liblab et al. (2020)	52 Ovarian cancer (Epithelial ovarian cancer)	CBP + PTX CBP mono-therapy	Higher incident rate of grade 2-4 anemia [46.34% (AA) vs. 81.82% (AG), *p* = 0.036]	Increase
*GSTM1* gene deletion				
Erčulj et al. (2012)	94 Malignant mesothelioma	DDP/CBP + GEM/PEM DDP + MMC + VCR	Decreased risk of grade 2-4 leukopenia (0/0 vs. 1/1 + 1/0 OR = 0.43; 95% CI: 0.18–0.99; *p* = 0.048)	Decrease
Khrunin et al. (2010)	104 Ovarian cancer	DDP + CTX	Decreased risk of grade 1-4 thrombocytopenia (0/0 vs. 1/0 OR = 0.13; 95% CI: 0.03–0.62; *p* = 0.005), grade 2-4 anemia (0/0 vs. 1/0 OR = 0.29; 95% CI: 0.13–0.66; *p* = 0.003)	Decrease
*ERCC1* rs11615 (C118T, Asn118Asn)				
Lee et al. (2013)	292 Colon cancer	L-OHP + LV + 5-FU	Increased risk of grade 3-4 neutropenia (TT vs. TC + CC OR = 4.58, 95% CI: 1.20–17.40, *p* = 0.026)	Increase
Lavanderos et al. (2019)	119 Testicular cancer	DDP + BLM + VP-16	Increased risk of grade 3-4 febrile neutropenia (TT vs. CC + CT OR = 4.89; 95% CI: 1.06–22.56; *p* = 0.042)	Increase
Lambrechts et al. (2015)	290 Ovarian cancer	CBP + PTX CBP mono-therapy	Increased risk of grade 3-4 anemia in additive model (OR = 1.61; 95% CI: 1.04–2.50; *p* = 0.031)	Increase
Zheng et al. (2017)	437 in the in a discovery cohort and 781 in the validation cohort NSCLC	DDP/CBP + NVB/GEM/PTX/TXT/PEM	Increased risk of grade 3-4 anemia in dominant model in discovery cohort (OR = 2.230; 95% CI: 1.041–4.775; *p* = 0.039)	Increase
Cortejoso et al. (2013)	106 Colorectal cancer	L-OHP + 5-FU + LV L-OHP + CAP	Decreased risk of grade 3-4 neutropenia (CT + TT vs. CC OR = 0.205; 95% CI: 0.061–0.690; *p* = 0.010)	Decrease
*ERCC1* rs3212986 (C8092A)				
Zheng et al. (2017)	437 in the in a discovery cohort and 781 in the validation cohort NSCLC	DDP/CBP + NVB/GEM/PTX/TXT/PEM	Decreased risk of grade 3-4 hematologic toxicity in recessive model in discovery cohort (OR = 0.326; 95% CI: 0.123–0.861; *p* = 0.024)	Decrease
Erčulj et al. (2012)	94 Malignant mesothelioma	DDP/CBP + GEM/PEM DDP + MMC + VCR	Decreased risk of grade 2-4 leukopenia (CA + AA vs. CC OR = 0.18; 95% CI: 0.04–0.86; *p* = 0.032)	Decrease
*ERCC2/XPD* rs13181(A>C, Lys751Gln)				
Isla et al. (2004)	62 NSCLC	DDP + TXT	Associated with grade 2-4 neutropenia (the incident rate of grade 2-4 neutropenia for Lys/Lys, Lys/Gln and Gln/Gln were 48%, 19%, and 14%, *p* = 0.04)	Decrease
*ERCC2/XPD* rs1799793 (G23591A, Asp312Asn)				
Erčulj et al. (2012)	94 Malignant mesothelioma	DDP/CBP + GEM/PEM DDP + MMC + VCR	Decreased risk of grade 1-4 thrombocytopenia (Asp/Asn + Asn/Asn vs. Asp/Asp OR = 0.15; 95% CI: 0.04–0.61; *p* = 0.008)	Decrease
Khrunin et al. (2010)	104 Ovarian cancer	DDP + CTX	Associated with grade 1-4 thrombocytopenia (Asp/Asn vs. Asp/Asp + Asn/Asn OR = 4.05; 95% CI: 1.21–13.58; *p* = 0.027), grade 2-4 anemia (Asp/Asn vs. Asp/Asp + Asn/Asn OR = 2.32; 95% CI: 1.05–5.13; *p* = 0.048)	Increase
*ERCC2/XPD* rs238406 (C22541A, Arg156Arg)				
(Wu et al. 2009)	209 NSCLC	DDP/CBP + NVB/GEM/PTX/TXTOthers DDP/CBP combinations	Increased risk of grade 3-4 hematologic toxicity (AA vs. CC OR = 3.24; 95% CI: 1.35–7.78; *p* = 0.009), grade 3-4 leukopenia toxicity (AA vs. CC OR = 4.88; 95% CI: 1.67–14.26; *p* = 0.005)	Increase
Lavanderos et al. (2019)	119 Testicular cancer	DDP + BLM + VP-16	Increased risk of grade 3-4 leukopenia (CA + AA vs. CC OR = 4.09; 95% CI: 1.04–15.99; *p* = 0.043)	Increase
*ERCC4 (XPF)* rs1799801(T>C, Ser835Ser)				
Zheng et al. (2017)	437 in the in a discovery cohort and 781 in the validation cohort NSCLC	DDP/CBP + NVB/GEM/PTX/TXT/PEM	Increased risk of grade 3-4 hematologic toxicity in additive model (OR = 1.555; 95% CI: 1.041–2.323; *p* = 0.031) and grade 3-4 thrombocytopenia in dominant model (OR = 3.562; 95% CI: 1.513–8.390; *p* = 0.004) in discovery cohort	Increase
*XPC* rs2228001(A>C, Lys939Gln)				
Bushra et al. (2020)	285 NSCLC	DDP/CBP + GEM/NVB/PTX/TXT	Decreased risk of grade 3-4 anemia (CC vs. AA OR = 0.18; 95% CI: 0.04–0.82; *p* = 0.027) and Increased risk of grade 3-4 neutropenia (AC vs. AA OR = 3.31; 95% CI: 1.74–6.31; *p* = 0.0003, AC + CC vs. AA OR = 2.63; 95% CI: 1.41–4.90; *p* = 0.002)	Increase
Zheng et al. (2017)	437 in the in a discovery cohort and 781 in the validation cohort NSCLC	DDP/CBP + NVB/GEM/PTX/TXT/PEM	Increased risk of grade 3-4 leukocytopenia in dominant model in discovery cohort (OR = 2.217; 95% CI: 1.054–4.665; *p* = 0.036)	Increase
*XPG/ERCC5* rs1047768 (T>C, His46His)				
Zheng et al. (2017)	437 in the in a discovery cohort and 781 in the validation cohort NSCLC	DDP/CBP + NVB/GEM/PTX/TXT/PEM	Increased risk of grade 3-4 leukocytopenia in additive model in discovery cohort (OR = 1.701; 95% CI: 1.021–2.835; *p* = 0.041)	Increase
*XPG/ERCC5* rs17655 (G>C, His1104Asp)				
Zheng et al. (2017)	437 in the in a discovery cohort and 781 in the validation cohort NSCLC	DDP/CBP + NVB/GEM/PTX/TXT/PEM	Increased risk of grade 3-4 thrombocytopenia in additive model in discovery cohort (OR = 2.165; 95% CI: 1.191–3.938; *p* = 0.011)	Increase
*XPCC1* rs25487 (G23885A, Arg399Gln)				
Bushra et al. (2020)	285 NSCLC	DDP/CBP + GEM/NVB/PTX/TXT	Increased risk of grade 3-4 anemia (AA+ AG vs. GG OR = 2.0; 95% CI: 1.19–3.35; *p* = 0.008, AG vs. GG OR = 2.27; 95% CI: 1.32–3.91; *p* = 0.003), grade 3-4 neutropenia (AG vs. GG OR = 2.37; 95% CI: 1.37–4.07; *p* = 0.002, AA+ AG vs. GG OR = 1.98; 95% CI: 1.18–3.33; *p* = 0.010), grade 3-4 leukopenia (AG vs. GG OR = 1.79; 95% CI: 1.0–3.18; *p* = 0.049) and grade 3-4 thrombocytopenia (AG vs. GG OR = 2.14; 95% CI: 1.09–4.20; *p* = 0.027, AA+ AG vs. GG OR = 2.11; 95% CI: 1.10–4.06; *p* = 0.025)	Increase
Peng et al. (2014)	235 NSCLC	DDP + PTX/GEM/NVB/PEM	Increased risk of grade 3-4 hematologic toxicity (AG vs. GG OR = 1.929; 95% CI: 1.069-3.481 *p* = 0.029, AA vs. GG OR = 4.885; 95% CI: 1.147–20.197; *p* = 0.032, AG + AA vs. GG OR = 2.135; 95% CI: 1.207–3.777; *p* = 0.009)	Increase
Zheng et al. (2017)	437 in the in a discovery cohort and 781 in the validation cohort NSCLC	DDP/CBP + NVB/GEM/PTX/TXT/PEM	Increased risk of grade 3-4 leukocytopenia in recessive model (OR = 2.841; 95% CI: 1.051–7.681; *p* = 0.040) and grade 3-4 thrombocytopenia in additive model (OR = 2.033; 95% CI: 1.113–3.715; *p* = 0.021) in discovery cohort	Increase
Deng et al. (2015)	97 NSCLC	DDP + GEM/NVB/PTX/TXT	Decreased risk of grade 1-4 lymphopenia (AG + AA vs. GG OR = 0.323; 95% CI: 0.121–0.862; *p* = 0.024)	Decrease
Khrunin et al. (2010)	104 Ovarian cancer	DDP + CTX	Decreased risk of grade 3-4 neutropenia (GG vs. AG + AA OR = 3.02; 95% CI: 1.33–6.88; *p* = 0.009)	Decrease
*APE1* rs1130409 (T>G, Asp148Glu)				
Zheng et al. (2017)	437 in the in a discovery cohort and 781 in the validation cohort NSCLC	DDP/CBP + NVB/GEM/PTX/TXT/PEM	Decreased risk of grade 3-4 leukocytopenia in dominant model (OR = 0.460; 95% CI: 0.241–0.879; *p* = 0.019), grade 3-4 neutropenia in dominant model (OR = 0.557; 95% CI: 0.321–0.967; *p* = 0.038) in discovery cohort	Decrease
*REV3* rs465646 (G>A)				
Ye et al. (2015)	663 NSCLC	DDP/CBP + NVB/GEM/PTX/TXTOthers DDP/CBP combinations	Increased risk of grade 3-4 hematologic toxicity (A/G + A/A vs. G/G OR = 2.54; 95% CI: 1.17–5.42; *p* = 0.016)	Increase
*REV1* rs3087386 (A>G, Phe257Ser)				
Goričar et al. (2014)	139 Malignant mesothelioma	DDP + GEM/PEM Other DDP doublets	Decreased risk of grade 2-4 neutropenia (GA + AA vs. GG OR = 0.38; 95% CI: 0.17–0.84; *p* = 0.017)	Decrease
*MTHFR* rs1801133 (C677T, Ala222Val)				
Walia et al. (2022)	123 Lung adenocarcinoma cancer	DDP/CBP + PEM	Increased risk of grade 1-3 neutropenia (CT vs. CC OR = 5.34; 95% CI: 1.49–19.06; *p* = 0.009, CT + TT vs. CC OR = 4.45; 95% CI: 1.28–15.43; *p* = 0.019)	Increase
Lee et al. (2013)	292 Colon cancer	L-OHP + LV + 5-FU	Increased risk of grade 3-4 neutropenia (TT vs. CC + CT OR = 2.32, 95% CI: 1.19–4.55, *p* = 0.014)	Increase
Li et al. (2014)	1004 NSCLC	DDP/CBP + NVB/GEM/PTX/TXTOther DDP/CBP combinations	Decreased risk of grade 3-4 thrombocytopenia (CT vs. CC OR = 0.40; 95% CI: 0.19–0.85; *p* = 0.016)	Decrease
*RRM1* rs12806698(-37C/A)				
Isla et al. (2004)	62 NSCLC	DDP + TXT	Associated with grade 2-4 leukopenia (the incident rate of grade 2-4 leukopenia for CC and CA were 31% and 10%, *p* = 0.05)	Decrease
Zheng et al. (2017)	437 in the in a discovery cohort and 781 in the validation cohort NSCLC	DDP/CBP + NVB/GEM/PTX/TXT/PEM	Increased risk of grade 3-4 leukocytopenia in recessive model (OR = 5.095; 95% CI: 2.132–12.170; *p* = 0.0002), grade 3-4 neutropenia in recessive model (OR = 2.561; 95% CI: 1.075–6.099; *p* = 0.034) in discovery cohort	Increase
*MDM2* rs2279744 (309T>G)				
Zheng et al. (2017)	437 in the in a discovery cohort and 781 in the validation cohort NSCLC	DDP/CBP + NVB/GEM/PTX/TXT/PEM	Decreased risk of grade 3-4 thrombocytopenia in additive model (OR = 0.472; 95% CI: 0.257–0.866; *p* = 0.015) in discovery cohort	Decrease
Wang et al. (2014)	119 SCLC	DDP + VP-16	Decreased risk of grade 3-4 neutropenia in additive model (OR = 0.48; 95% CI: 0.2652–0.8709; *p* = 0.015) and in recessive model (OR = 0.27; 95% CI: 0.08763–0.8859; *p* = 0.030)	Decrease
Guo et al. (2016)	292 Lung adenocarcinoma	DDP/CBP combinations	Increased risk of grade 3-4 hematologic toxicity in recessive model (OR = 2.128; 95% CI: 1.198–3.777; *p* = 0.010)	Increase
*TP53* rs1042522 (C>G, Pro72Arg)				
Khrunin et al. (2010)	104 Ovarian cancer	DDP + CTX	Increased risk of grade 3-4 neutropenia (GG vs. CC + CG OR = 8.57; 95% CI: 1.05–69.8; *p* = 0.023)	Increase
Wang et al. (2014)	119 SCLC	DDP + VP-16	Increased risk of grade 3-4 neutropenia in recessive model (OR = 3.44; 95% CI: 1.302–9.111; *p* = 0.012)	Increase

Abbreviations: BLM, bleomycin; CAP, capecitabine; CBP, carboplatin; CI, confidence interval; CPT-11, irinotecan; CTX, cyclophosphamide; DDP, cisplatin; GEM, gemcitabine; L-OHP, oxaliplatin; LV, leucovorin; MMC, mitomycin C; NSCLC, non-small cell lung cancer; NVB, navelbine; OR, odds ratios; PEM, pemetrexed; PTX, paclitaxel; SCLC, small cell lung cancer; TXT, docetaxel; VCR, vincristine; VP-16, etoposide; 5-FU, fluorouracil.

#### 3.3.1 Transportation for influx

##### 3.3.1.1 SLC31A1 (CTR1)

The main route of platinum agents entering the cell is through the copper transporter CTR1 (SLC31A1) (Giuliano, 2012). Because of the lack of candidate SNPs, studies were limited. *SLC31A1(CTR1)* rs10981699 (Xu et al., 2012; Sun et al., 2018), rs10817465 (Xu et al., 2012; Sun et al., 2018), rs12686377 (Xu et al., 2012; Kumpiro et al., 2016) were analyzed in two studies respectively. No association has been found.

#### 3.3.2 Transportation for efflux

##### 3.3.2.1 ABCC2 (MRP2)

The multi-drug resistance protein (MRP2), encoded for by *ABCC2*, involved in pumping cisplatin out of the cell (Cara and M Eileen, 2006). The frequently investigated genetic polymorphisms in *ABCC2* were rs717620 (C-24T) (n = 6), rs3740066 (C3972T, Ile1324Ile) (n = 5), rs2273697 (G1249A) (n = 4), rs12762549(*+9383C>G) (n = 2), and rs2073337 (c.1668 + 148A>G) (n = 2). *ABCC2* rs717620 (C-24 T) and *ABCC2* rs3740066 (C3972T, Ile1324Ile) may not affect the expression of MRP2 mRNA or protein (Moriya et al., 2002; Meyer zu Schwabedissen et al., 2005), while *ABCC2* rs2273697 (G1249A) resulted in a significantly reduced expression of mRNA in human preterm placenta (Meyer zu Schwabedissen et al., 2005).

Positive association were reported in only few studies. *ABCC2* rs717620 (C-24T) CC genotype present increased rate of grade 3-4 thrombocytopenia in contrast to CT and TT genotypes in 292 colon cancer patients treated with FOLFOX chemotherapy (5.6% vs. 0.8%, *p* = 0.047) (Lee et al., 2013). *ABCC2* rs3740066(C3972T, Ile1324Ile) T allele was suggested as a risk factor for grade 3-4 thrombocytopenia in 445 NSCLC patients receiving platinum-based chemotherapy (OR = 2.43; 95% CI: 1.06–5.56; *p* = 0.034) (Han et al., 2011). As for *ABCC2* rs12762549(G1249A), two studies show inconsistent results. Nomura et al. found a higher risk of grade 3-4 neutropenia in GG genotype in 158 esophageal cancer patients treated with docetaxel, cisplatin, and 5-fluorouracil chemotherapy (OR = 2.342; 95% CI: 1.108–4.948; *p* = 0.026) (Nomura et al., 2020). In contrast, *ABCC2* rs12762549 is associated with decreased risk of grade 3-4 anemia in additive model (OR = 0.51; 95% CI: 0.33–0.81; *p* = 0.004) among 290 ovarian cancer patients upon treatment with paclitaxel and carboplatin (Lambrechts et al., 2015). No association has been found with the *ABCC2* rs2073337 (Marsh et al., 2007; Lambrechts et al., 2015) and rs2273697 (Han et al., 2007; Marsh et al., 2007; Han et al., 2011; Qian et al., 2016).

##### 3.3.2.2 ABCB1 (MDR1)

ABCB1 (MDR1 or p-glycoprotein) is thought to play a role in platinum efflux, although to a much lesser extent than the copper transporters or MRP2 (Cara and M Eileen, 2006). As to *ABCB1* rs1045642(C3435T), the homozygous T-allele is associated with significantly lower duodenal MDR-1 expression and the highest digoxin plasma levels (Hoffmeyer et al., 2000). 12 studies were available for *ABCB1* rs1045642(C3435T) but the results have not been consistent. De Troia B et al. reported that patients carrying 3435T allele had a lower risk of grade 3-4 neutropenia in 82 lung cancer patients treated with platinum-based chemotherapy (OR = 0.24; 95% CI: 0.07–0.75; *p* = 0.01) (De Troia et al., 2019). In contrast to these results, Nomura, H. et al. found T allele carriers were more likely to develop grade 3-4 neutropenia (OR = 2.191; 95% CI: 1.087–4.417; *p* = 0.028) in 158 esophageal cancer patients receiving docetaxel, cisplatin, and 5-fluorouracil therapy (Nomura et al., 2020).

Studies have also focused on *ABCB1* rs2032582 (G2677TA, Ala893Ser) (n = 7) and *ABCB1* rs1128503(C1236T) (n = 6). The two SNPs are associated with altered P-glycoprotein function, in which the Ser893 variant transporter resulted in a 47% lower intracellular digoxin concentration (*p* < .002) than did the Ala893 variant *in vitro* (Kim et al., 2001) and homozygous carriers of *ABCB1* rs1128503(C1236T) polymorphism was significantly correlated with a decreased docetaxel clearance (Bosch et al., 2006). *ABCB1* rs2032582 (G2677TA, Ala893Ser) displayed differential genotypic distribution between groups with grade 4 neutropenia and grade 1-3 neutropenia in 107 NSCLC patients (*p* = 0.030) (Han et al., 2007). *ABCB1* rs1128503 (C1236T) were significantly associated with grade 3-4 anemia in additive model (OR = 1.71; 95% CI: 1.07–2.71; *p* = 0.023) in 290 ovarian cancer patients (Lambrechts et al., 2015). Similarly, another study in 112 ovarian cancer patients found that carriers of TT genotype were more frequently experienced grade 1-4 thrombocytopenia compared to those CC or CT genotype (OR = 3.50; 95% CI: 1.12–10.97; *p* = 0.03) (Ferracini et al., 2021).

##### 3.3.2.3 ABCG2 (BCRP)

ABCG2 (breast cancer resistance protein or BCRP) is efflux transporter proteins that play a role in the development of chemoresistance to platinum agents (Ding-Wu et al., 2012). No association has been shown with the *ABCG2* rs2231142 (Han et al., 2007; Marsh et al., 2007; Lambrechts et al., 2015; Nomura et al., 2020; Wang et al., 2021) and *ABCG2* rs2231137 (Han et al., 2007; Nomura et al., 2020; Wang et al., 2021).

##### 3.3.2.4 OCT2 (SLC22A2) and MATE1 (SLC47A1)

OCT2 is considered the predominant transporter mediating active accumulation of cisplatin in the kidney. MATE1 is thought to mediate the final step of renal tubular secretion of cisplatin (Giuliano, 2012). The *OCT2* rs316019 (808G/T, p.270Ala > Ser) variant significantly impaired uptake kinetics of endogenous compounds and drugs (Zolk et al., 2009; Zolk, 2012). *OCT2* rs316019 (808G/T, p.270Ala > Ser) and *MATE1* rs2289669 (G/A) were analyzed in two studies (Iwata et al., 2012; Qian et al., 2016). Qian et al. found significant correlations with grade 3-4 hematological toxicity in these two SNPs among 403 NSCLC patients (Qian et al., 2016).

#### 3.3.3 Metabolism

Platinum compounds can be detoxified by conjugation with glutathione through the aid of glutathione S-transferases (GSTs) (R et al., 2000). GSTP1, GSTM1, and GSTT1 belongs to Human GSTs and were mostly analyzed for the functional polymorphisms in gene regions.

##### 3.3.3.1 GSTP1

Two common nonsynonymous polymorphisms in *GSTP1*, rs1695 (Ile105Val, A313G) and rs1138272 (Ala114Val), were shown to decrease GSTP1 enzyme activity (Moyer et al., 2008). *GSTP1* rs1695 (Ile105Val, A313G) was the most common studied variant with 18 studies available. Four studies show a protective effect for hematological toxicity. One study in Brazil reported AG or GG genotype was associated with decreased risk of grade 3-4 anemia and grade 3-4 thrombocytopenia in 112 epithelial ovarian cancer patients (Ferracini et al., 2021). Another study in Korean showed that patients with A/G or G/G genotype showed lower rate of hematological toxicity (13/24, 54.2%) than those with A/A genotype (74/94, 78.7%) among 118 epithelial ovarian cancer patients (*p* = 0.015) (Kim et al., 2009). Bushra et al. found that GG genotype is associated with decreased risk of grade 3-4 anemia (OR = 0.29; 95% CI: 0.10–0.87; *p* = 0.027) and grade 3-4 neutropenia (OR = 0.31; 95% CI: 0.10–0.96; *p* = 0.043) in 285 NSCLC patients in Bangladesh (Bushra et al., 2020). Perhaps worth to mention is a comprehensive pharmacogenomic analysis in 320 gynecological cancers patients, of which *GSTP1* rs1695 showed the lowest *p*-value against severe hematological toxicity (*p* = 0.00034) (Yoshihama et al., 2018). In contrast, Walia et al. found a significantly increased risk of grade 3-4 anemia with the AG genotype (OR = 2.12; 95% CI: 0.97–4.62; *p* = 0.04) and increased risk of grade 2-4 leukopenia with the GG genotype (OR = 2.41; 95% CI: 1.39–4.18; *p* = 0.001) in 317 North Indian lung cancer patients (Walia et al., 2021). Liblab et al. (2020) found that carriers of AG genotype were more frequently experienced grade 2-4 anemia than AA genotype in 52 ovarian cancer patients (81.82% vs. 46.34%, *p* = 0.036). Five studies demonstrated no significant association of *GSTP1* rs1138272 (Ala114Val) polymorphism.

##### 3.3.3.2 GSTT1 and GSTM1

Gene deletion in GSTT1 and GSTM1 can lead to an absence of enzymatic activity (Pemble et al., 1994; Xu et al., 1998). Nine studies showed no effect of *GSTT1* gene deletion on the risk of hematological toxicity. Eight studies analyzed *GSTM1* gene deletion, in which two studies showed a protective effect (Khrunin et al., 2010; Erčulj et al., 2012). In 94 malignant mesothelioma patients treated with platinum-based chemotherapy, Patients with homozygous *GSTM1* gene deletion have a significantly decreased risk of grade 2-4 leukopenia compared with those of at least one functional allele (OR = 0.43; 95% CI: 0.18–0.99; *p* = 0.048) (Erčulj et al., 2012). Another study in 104 ovarian cancer patients that received paclitaxel and carboplatin found carriers of a homozygous *GSTM1* gene deletion were less likely to develop grade 1-4 thrombocytopenia (OR = 0.13; 95% CI: 0.03–0.62; *p* = 0.005) and grade 2-4 anemia (OR = 0.29; 95% CI: 0.13–0.66; *p* = 0.003) than those of functional GSTM1 variants (Khrunin et al., 2010).

#### 3.3.4 NER pathway

The bulky DNA intra-strand adducts generated by platinum are mainly repaired by the nucleotide-excision repair (NER) pathway (Clarissa Ribeiro Reily et al., 2018).

##### 3.3.4.1 ERCC1

ERCC1 interact with XPF to make an incision at the damaged DNA lesion in NER pathway. *ERCC1* rs11615 (C118T, Asn118Asn) (n = 19) and *ERCC1* rs3212986 (C8092A) (n = 12) have been wildly studied. *ERCC1* rs11615 reduces the transcription and mRNA levels of *ERCC1*, resulting in lower ERCC1 expression (Yu et al., 2000). *ERCC1* rs3212986 resides in the 30-untranslated region that might affect mRNA stability (Chen et al., 2000). Four studies reported a significantly increased risk with *ERCC1* rs11615 (C118T, Asn118Asn). In a study of 292 colon cancer patients receiving adjuvant oxaliplatin plus leucovorin plus 5-fluorouracil (FOLFOX) chemotherapy, TT genotype was more prone to develop grade 3-4 neutropenia (OR = 4.58, 95% CI: 1.20–17.40, *p* = 0.026) (Lee et al., 2013). Another study in 119 testicular cancer patients also reported an increased risk of grade 3-4 febrile neutropenia caused by bleomycin, etoposide, and cisplatin (BEP) chemotherapy in TT genotype (OR = 4.89; 95% CI: 1.06–22.56; *p* = 0.042) (Lavanderos et al., 2019). *ERCC1* rs11615 was associated with an increased risk of grade 3-4 anemia in 290 ovarian cancer patients (OR = 1.61; 95% CI: 1.04–2.50; *p* = 0.031) (Lambrechts et al., 2015) and 437 NSCLC patients (OR = 2.230; 95% CI: 1.041–4.775; *p* = 0.039) (Zheng et al., 2017). On the contrary, Cortejoso et al. (2013) found individuals carrying T allele had a reduced risk of grade 3-4 neutropenia in 106 colon cancer patients (OR = 0.205; 95% CI: 0.061–0.690; *p* = 0.010). As for *ERCC1* rs3212986(C8092A), two studies reported a decreased risk. Zheng et al. found that *ERCC1* rs3212986 was significantly associated with a reduced risk of grade 3-4 hematologic toxicity in recessive model in 437 NSCLC patients (OR = 0.326; 95% CI: 0.123–0.861; *p* = 0.024) (Zheng et al., 2017). Erčulj et al. (2012) reported that patients carrying A allele had a lower risk of grade 2-4 leukopenia in 94 malignant mesothelioma patients (OR = 0.18; 95% CI: 0.04–0.86; *p* = 0.032). No association has been established between *ERCC1* rs3212961 (17677G>T) and the development of hematologic toxicity (Marsh et al., 2007; Lambrechts et al., 2015; Song et al., 2017).

##### 3.3.4.2 ERCC2/XPD

ERCC2 (XPD) is a helicase that can unwind the DNA strands to facilitate the binding of other NER proteins. *ERCC2* rs13181 (Lys751Gln), rs1799793(Asp312Asn), rs238406 (C22541A, Arg156Arg), rs1052555 (Asp711Asp) have been evaluated in 17 studies, 13 studies, five studies and three studies respectively. *ERCC2* rs13181(Lys751Gln) can alter mRNA transcription levels, leading to reduced levels of *ERCC2* mRNA(82)*.* For *ERCC2* rs13181 (Lys751Gln), grade 2-4 neutropenia was more frequent among individual with 751 Lys/Lys genotype in 62 NSCLC patients treated with cisplatin and docetaxel (*p* = 0.04) (Isla et al., 2004).

*ERCC2* rs1799793 (Asp312Asn) is associated with significantly decreased constitutive XPD mRNA levels in lymphocytes of healthy subjects (Wolfe et al., 2007). Results on rs1799793 (Asp312Asn) have not been consistent. Erčulj et al. found patients carrying 312Asp/Asn and 312Asn/Asn genotype had a lower risk of grade 1-4 thrombocytopenia in 94 malignant mesothelioma patients (OR = 0.15; 95% CI: 0.04–0.61; *p* = 0.008) (Erčulj et al., 2012). Whereas, Khrunin et al. (2010) found patients carrying Asp/Asn genotype were more prone to grade 1-4 thrombocytopenia (OR = 4.05; 95% CI: 1.21–13.58; *p* = 0.027) and grade 2-4 anemia (OR = 2.32; 95% CI: 1.05–5.13; *p* = 0.048) in 104 ovarian cancer patients. *ERCC2* rs238406(C22541A, Arg156Arg) can reduce the levels of mRNA and ultimately affect XPD activity and function (Wolfe et al., 2007). The incidence of grade 3-4 hematologic toxicity (OR = 3.24; 95% CI: 1.35–7.78; *p* = 0.009) and grade 3-4 leukopenia (OR = 4.88; 95% CI: 1.67–14.26; *p* = 0.005) was significantly higher in variant homozygotes AA genotype, when compared with CC genotype in 209 NSCLC patients (Wu et al., 2009). Likewise, another study in 119 testicular cancer patients showed the increased risk of grade 3-4 leukopenia with the AA or CA genotype (OR = 4.09; 95% CI: 1.04–15.99; *p* = 0.043) (Lavanderos et al., 2019). Studies on *ERCC2* rs1052555 (Asp711Asp) (n = 3) showed no significant difference (Wu et al., 2009; Song et al., 2017; Zheng et al., 2017).

##### 3.3.4.3 ERCC4/XPF

ERCC4/XPF participates in the removal of damaged DNA strands by acting as an endonuclease with ERCC1. Of two studies in *ERCC4* rs1799801 (T>C, Ser835Ser) (Song et al., 2017; Zheng et al., 2017), one study in 437 NSCLC patients demonstrated an association of the *ERCC4* rs1799801 with grade 3-4 hematologic toxicity in additive model (OR = 1.555; 95% CI: 1.041–2.323; *p* = 0.031) and grade 3-4 thrombocytopenia in dominant model (OR = 3.562; 95% CI: 1.513–8.390; *p* = 0.004) (Zheng et al., 2017).

##### 3.3.4.4 XPC

XPC cooperates with RAD23B to recognize DNA lesions and start the initial step of damage recognition. Three studies concentrate on *XPC* rs2228001(A>C, Lys939Gln) (Song et al., 2017; Zheng et al., 2017; Bushra et al., 2020). *XPC* rs2228001(A>C, Lys939Gln) was associated with lower DNA repair capacity (Zhu et al., 2008). In 285 Bangladesh NSCLC patients, carriers of *XPC* rs2228001 AC or CC genotypes showed significant suffering from grade 3-4 neutropenia (OR = 2.63; 95% CI: 1.41–4.90; *p* = 0.002), while those harboring CC genotypes are less likely to develop grade 3-4 anemia, compared with AA genotype carriers (OR = 0.18; 95% CI: 0.04–0.82; *p* = 0.027) (Bushra et al., 2020). Another study conducted in 437 Chinese NSCLC patients show *XPC* rs2228001 is associated with an increased risk of grade 3-4 leukocytopenia in dominant model (OR = 2.217; 95% CI: 1.054–4.665; *p* = 0.036) (Zheng et al., 2017).

##### 3.3.4.5 ERCC5/XPG

ERCC5 (XPG) encodes a structure-specific endonuclease that can cleave the 3ʹ-end of damaged DNA lesions. Two studies focus on *XPG* rs1047768(A>C, Lys939Gln) and rs17655(A>C, Lys939Gln). Zheng et al. found a significant association of *XPG* rs1047768 with grade 3-4 leukocytopenia in additive model (OR = 1.701; 95% CI: 1.021–2.835; *p* = 0.041) and *XPG* rs17655 with grade 3-4 thrombocytopenia in additive model (OR = 2.165; 95% CI: 1.191–3.938; *p* = 0.011) (Zheng et al., 2017). The other study reported no association with these two investigated SNPs (Song et al., 2017).

##### 3.3.4.6 Other NER genes

*XPA* rs1800975, *RPA1* rs17734, *RPA1* rs12727 and *CCNH* rs2230641 (Song et al., 2017; Zheng et al., 2017) were examined twice and showed no significant associations.

#### 3.3.5 BER pathway

The base excision repair (BER) pathway is mainly responsible for removing the oxidative DNA lesions generated by platinum drug exposure (Jana et al., 2018).

##### 3.3.5.1 XRCC1

XRCC1 acts as a central scaffolding protein to finish the final steps of BER. *XPCC1* rs25487 (G>A, Arg399Gln) can cause missense mutation in the coding region, leading to decreased damage DNA repair activity (Wang et al., 2003; Vodicka et al., 2004). 14 studies investigated a link of *XPCC1* rs25487 (G>A, Arg399Gln) with hematological toxicity and results have not been consistent. In a study of 285 NSCLC patients, *XRCC1* rs25487 polymorphism showed significant associations with increased risk of grade 3-4 anemia, grade 3-4 neutropenia, grade 3-4 leukopenia and grade 3-4 thrombocytopenia (Bushra et al., 2020). A higher risk of grade 3-4 hematologic toxicity was also observed in AG and GG carriers in a cohort of 235 NSCLC patients (OR = 2.135; 95% CI: 1.207–3.777; *p* = 0.009) (Peng et al., 2014). In keeping with this, *XRCC1* rs25487 was identified as risk factors for grade 3-4 leukocytopenia in recessive model (OR = 2.841; 95% CI: 1.051–7.681; *p* = 0.040), grade 3-4 thrombocytopenia in additive model (OR = 2.033; 95% CI: 1.113–3.715; *p* = 0.021) in discovery cohort of 437 NSCLC patients (Zheng et al., 2017). In contrast, AA or AG carriers had a decreased risk of grade 1-4 lymphopenia as observed in 94 NSCLC patients (OR = 0.323; 95% CI: 0.121–0.862; *p* = 0.024) (Deng et al., 2015). In 104 ovarian cancer that received cisplatin and cyclophosphamide, an increased risk of grade 3-4 neutropenia was found in GG wildtype carriers (OR = 3.02; 95% CI: 1.33–6.88; *p* = 0.009) (Khrunin et al., 2010). Other studies failed to reveal a positive association.

*XPCC1* rs1799782 (Arg194Trp) can leading to decreased DNA repair activity (Wang et al., 2003)*. XPCC1* rs1799782 (Arg194Trp) (n = 4) (Wang et al., 2008; Kim et al., 2009; Khrunin et al., 2010; Lee et al., 2013) and *XPCC1* rs25489 (Arg280His) (n = 3) (Khrunin et al., 2010; Lee et al., 2013; Zheng et al., 2017) showed no significant difference with hematological toxicity.

##### 3.3.5.2 APE1 and OGG1

APE1 and OGG1 are key component in the BER pathway. Two studies examined *APE1* rs1130409 (Asp148Glu) and *OGG1* rs1052133 (Ser326Cys) (Peng et al., 2014; Zheng et al., 2017). *APE1* rs1130409(Asp148Glu) variant exhibiting normal *in vitro* nuclease capacity (Daviet et al., 2007; Wilson et al., 2011) and *OGG1* rs1052133(Ser326Cys) polymorphism negatively impacts OGG1 function (Wilson et al., 2011). *APE1* rs1130409 was associated with a decreased risk of grade 3-4 leukocytopenia (OR = 0.460; 95% CI: 0.241–0.879; *p* = 0.019) and grade 3-4 neutropenia (OR = 0.557; 95% CI: 0.321–0.967; *p* = 0.038) in dominant model (Zheng et al., 2017). *OGG1* rs1052133 failed to show significant association with hematological toxicity (Peng et al., 2014; Zheng et al., 2017).

#### 3.3.6 DSB pathway

Homologous recombination (HR) and non-homologous and joining pathways are responsible for repairing the DSBs generated by platinum-induced ICLs which are the most hazardous type of DNA damage. XRCC3 are one of the crucial proteins involved in mediating the HR pathway (Giovanna and Massimo, 2019). *XRCC3* rs861539(C>T, Thr241Met) was show to reduce DNA damage repair capacity (Matullo et al., 2001). However, no association was detected in three analyses on *XRCC3* rs861539 (Thr241Met) (Ludovini et al., 2011; Ruzzo et al., 2014; Zheng et al., 2017).

#### 3.3.7 TLS

TLS is performed by a series of low-fidelity polymerases to tolerate platinum-induced DNA lesions (The Vinh and Orlando, 2010). *REV3L* rs462779 (Goričar et al., 2014; Zheng et al., 2017), *REV3L* rs465646 (Goričar et al., 2014; Ye et al., 2015; Zheng et al., 2017), *REV7* rs746218 (Ye et al., 2015; Zheng et al., 2017), *REV7* rs2233006 (Ye et al., 2015; Zheng et al., 2017), *REV1* rs3087386 (Goričar et al., 2014; Zheng et al., 2017) and *Rad18* rs373572 (Chu et al., 2016; Zheng et al., 2017) were analyzed in more than one study. *REV3* rs465646 were associated with grade 3-4 hematologic toxicity in 663 NSCLC patients (OR = 2.54; 95% CI: 1.17–5.42; *p* = 0.016) (Ye et al., 2015). In a study of 139 malignant mesothelioma patients receiving cisplatin-based chemotherapy, carriers of the *REV1* rs3087386 A allele were less prone to develop grade 2-4 neutropenia (OR = 0.38; 95% CI: 0.17–0.84; *p* = 0.017) (Goričar et al., 2014). Other studies show no significant associations.

#### 3.3.8 DNA synthesis

##### 3.3.8.1 MTHFR

MTHFR is involved in folate metabolism, essential for the synthesis of nucleic acids and amino acids. Two common nonsynonymous polymorphisms in MTHFR, rs1801133 (C677T, Ala222Val) and rs1801131 (A1298C, Glu429Ala) were shown to decrease MTHFR enzyme activity *in vitro* (van der Put et al., 1998). These two SNPs were analyzed in six studies (Lee et al., 2013; Corrigan et al., 2014; Kanazawa et al., 2014; Li et al., 2014; Ruzzo et al., 2014; Walia et al., 2022). The results for *MTHFR* rs1801133 (C677T, Ala222Val) were inconsistent. An increased risk of grade 1-3 neutropenia was detected in carriers of the T allele (OR = 4.45; 95% CI: 1.28–15.43; *p* = 0.019) among 123 lung adenocarcinoma patients treated with platinum and pemetrexed chemotherapy (Walia et al., 2022). Likewise, carriers of the TT genotype were more likely to develop grade 3-4 neutropenia compared with those carrying CC and CT genotype in 292 colon cancer patients (OR = 2.32, 95% CI: 1.19–4.55, *p* = 0.014) (Lee et al., 2013). While, the heterozygote CT genotype show a protective effect for grade 3-4 thrombocytopenia in Li et al.’s study of 1004 NSCLC patients (OR = 0.40; 95% CI: 0.19–0.85; *p* = 0.016) (Li et al., 2014). *MTHFR* rs1801131 (A1298C, Glu429Ala) showed no effect on risk of hematological toxicity.

##### 3.3.8.2 RRM1

RRM1 encodes the regulatory subunit of ribonucleotide reductase and is involved in the production of deoxyribonucleotides during DNA synthesis. *RRM1* – 37A allele impacted promoter activity with increased RRM1 mRNA expression *in vitro* (Bepler et al., 2005; Rodriguez et al., 2011). *RRM1* rs12806698 (−37C/A) was suggested as a candidate risk factor for grade 3-4 leukocytopenia (OR = 5.095; 95% CI: 2.132–12.170; *p* = 0.0002) and grade 3-4 neutropenia (OR = 2.561; 95% CI: 1.075–6.099; *p* = 0.034) in recessive model in 437 NSCLC patients (Zheng et al., 2017), while in another study of 63 NSCLC patients, grade 2-4 leukopenia was more frequent among patients with wild-type *RRM1* rs12806698 CC genotype than carriers of CA genotype (37% versus 10%, *p* = 0.05) (Isla et al., 2004).

#### 3.3.9 Apoptosis

##### 3.3.9.1 MDM2

*MDM2* rs2279744 (309T>G) resulted in higher levels of MDM2 RNA and protein and the subsequent attenuation of the p53 DNA damage response (Bond et al., 2004). For *MDM2* rs2279744 (309T>G) (n = 4), two studies in lung cancer patients observed a protective effect for grade 3-4 thrombocytopenia (OR = 0.472; 95% CI: 0.257–0.866; *p* = 0.015) (Zheng et al., 2017) and grade 3-4 neutropenia (OR = 0.27; 95% CI: 0.08763–0.8859; *p* = 0.030) respectively (Wang et al., 2014). Another study in 292 lung adenocarcinoma patients show opposite results that *MDM2* rs2279744 was associated with an increased risk of grade 3-4 hematologic toxicity in recessive model (OR = 2.128; 95% CI: 1.198–3.777; *p* = 0.010) (Guo et al., 2016).

##### 3.3.9.2 TP 53

TP53 (p53) is a key regulator of cell cycle control and DNA damage response as well as apoptosis initiation. *TP53* rs1042522 (Arg72Pro) Arg72 variant is more efficient than the Pro72 variant in inducing apoptosis (Dumont et al., 2003). *TP53* rs1042522 (Arg72Pro) was associated with an increased risk of grade 3-4 neutropenia in recessive model (OR = 3.44; 95% CI: 1.302–9.111; *p* = 0.012) in 119 SCLC patients treated with cisplatin and etoposide (Wang et al., 2014). Another study in 104 ovarian cancer patients showed similar result that patients carrying GG genotype were prone to experience grade 3-4 neutropenia, compared with CC + CG genotype (OR = 8.57; 95% CI: 1.05–69.8; *p* = 0.023) (Khrunin et al., 2010). The remaining studies (n = 7) showed no significant difference.

### 3.4 Significant associations in other less commonly investigated variants

The array of SNPs that investigated only once are listed in Supplementary Table S8. Lots of studies analyzed those less commonly investigated variants in main candidate genes such as *SLC31A1 (CTR1)* (Xu et al., 2012; Sun et al., 2018), *ATP7A* (Deng et al., 2015; Li et al., 2018), *ATP7B* (Li et al., 2018), *ABCC2* (Ruzzo et al., 2014), *ABCB1* (Chen et al., 2010; Chen et al., 2016), *ABCG2* (Chen et al., 2016; Wang et al., 2021), *OCT2* (Chen et al., 2016; Qian et al., 2016), *MDM2* (Zheng et al., 2014; Qian et al., 2015), *TP53* (Khrunin et al., 2010) or tagSNPs on genes that are not directly affect the influx, efflux and metabolism of platinum agent or play as mediators or modulators of center cellular activities. Those genes and the functions were listed in Supplementary Table S8, including the transport genes (*SLCs*, *AQP2*, *AQP9*, *TMEM205*, *SIRT1*, *ABCC1*, *MVP*, *AQP1*) (Chen et al., 2016; Senk et al., 2019), Metabolism gene (*CYP2E1*, *GSTA1*, *GSTM3*, *AGXT*, *MAPT*, *MPO*) (Marsh et al., 2007; Khrunin et al., 2010; Lee et al., 2013), MicroRNAs (Zhan et al., 2012; Fang et al., 2017), Long Non-Coding RNA (Hu et al., 2016; Gong et al., 2017). Apoptosis-related genes (*BCL2*, *BAX*, *CASP3*, *CASP8*, *CASP10*, *TNFα* and *MIF*) (Gu et al., 2012; Qian et al., 2012; Liu D. et al., 2017a), *EPO* (Zheng et al., 2021), *VCP* (Peng et al., 2013), *STAT3* (Gong et al., 2019), *WISP1* (Chen et al., 2014), *TERT* (Zhao et al., 2015), *CHEK2* (Xu et al., 2016), *MMP-2* (Zhao et al., 2012), *RICTOR* (Wang et al., 2016), *eIF3 a* (Yin et al., 2015), HSP genes and Rho family genes (Zou et al., 2016), CDC25 family genes (Cai et al., 2014), The JNK and P38 MAPK pathways genes (Jia et al., 2016), MIF signaling pathway (Tan et al., 2014). As to DNA repair pathway, there were studies focus on tagSNPs in less investigated NER genes (Chen et al., 2016; Song et al., 2017; Zheng et al., 2017) and BER genes (Peng et al., 2014; Zheng et al., 2017). DNA repair pathway that not directly involved in repairing platinum-induced DNA lesions like MMR (Liu JY. et al., 2017b; Zheng et al., 2017), DSB (Zheng et al., 2017), TLS (Goričar et al., 2014; Shao et al., 2014; Ye et al., 2015; Chu et al., 2016; Zheng et al., 2017), FA pathway (Zheng et al., 2017), have received more attention recently. Some significant association was found, while these SNPs were not previously analyzed and should be validated in future studies.

### 3.5 High throughput researches

Four studies are derived from the same cohort in Sweden population of 215 NSCLC patients treated with gemcitabine/carboplatin chemotherapy (Gréen et al., 2016; Svedberg et al., 2020; Björn et al., 2020a; Björn et al., 2020b). Three of them were whole-exome sequence studies concentrating of gemcitabine/carboplatin-induced grade 3-4 thrombocytopenia (Gréen et al., 2016; Björn et al., 2020a), leukopenia (Svedberg et al., 2020) and neutropenia (Gréen et al., 2016; Svedberg et al., 2020). The fourth study performed a GWAS in a subset of 96 patients (Björn et al., 2020b). These studies identified and validated several genetic variations, genes and hematopoiesis-related pathways to be potential significance and created weighted genetic risk score (wGRS) prediction models for predicting the risk of chemotherapy-induced hematological toxicity. There were GWAS concentrating on NSCLC (Cao et al., 2016), cervical cancer (Huang et al., 2015), and unclassified carcinomas (Low et al., 2013). A list of novel genetic variants such as rs13014982 at 2q24.3 and rs9909179 at 17p12 were identified (Cao et al., 2016) and the artificial neural networks model based on the multiple risk factors were constructed (Huang et al., 2015). Yin et al. developed a strategy to establish a predicted model of toxicity integrating both genetic and clinical factors using DM techniques (Yin et al., 2016).

## 4 Discussion

### 4.1 Main findings

This systematic review has reported and evaluated the findings of published studies that investigated the genetic associations with hematological toxicities in the cancer patients receiving platinum-based chemotherapy. We found that *ABCC2* rs12762549, *ABCB1* rs1045642, *ABCB1* rs1128503, *GSTP1* rs1695, *GSTM1* gene deletion, *ERCC1* rs11615, *ERCC1* rs3212986, *ERCC2* rs238406, *ERCC2* rs1799793, *XPC* rs2228001, *XPCC1* rs25487, *MTHFR* rs1801133, *MDM2* rs2279744, *RRM1* rs12806698, *TP53* rs1042522 show positive associations in more than two studies, but most associations were not consistently replicated across the reviewed studies. Among them, *ABCB1* rs1128503, *GSTP1* rs1695, *GSTM1* gene deletion, *ERCC1* rs11615, *ERCC1* rs3212986, *ERCC2* rs238406, *XPC* rs2228001, *XPCC1* rs25487, *MTHFR* rs1801133, *MDM2* rs2279744, *TP53* rs1042522 had consistent results across at least two independent populations. These genetic variants may provide insights into the molecular mechanisms towards platinum-induced hematological toxicities (Qihai et al., 2023).

It's also worth mentioning that *GSTP1* rs1695 (A313G, Ile105Val) show significant association in six studies. Among them, four studies show protective role of *GSTP1* rs1695 against platinum-induced hematological toxicities, including a comprehensive analysis that targeted resequencing of 100 notable pharmacokinetics-related genes in which *GSTP1* rs1695 showed the smallest *p*-value (*p* = 0.00034) (Yoshihama et al., 2018). Positive associations of *GSTP1* rs1695 with increased risk of platinum-induced hematological toxicity was found in two meta-analysis (Lv et al., 2018; Kim et al., 2022), while these two meta-analysis studies face the limitations of insufficient data availability. Alteration of DNA repair ability might play an important role in the development of platinum-induced hematological toxicity. Genetic variants in the candidate NER genes may affect the repair function and are most promising in predicting platinum-related hematological toxicity, since it is the main pathway responsible of repairing the bulky DNA intra-strand adducts generated by platinum agents (Hilary et al., 2024). *ERCC1* rs11615 (C118T, Asn118Asn) and *ERCC1* rs3212986 (C8092A) are two common variant that affect ERCC1 (key enzyme in NER pathway) mRNA expression or mRNA stability (Chen et al., 2000; Yu et al., 2000). *ERCC1* rs11615 present consistent results with increased risk of grade 3-4 neutropenia or anemia in four studies, and *ERCC1* rs3212986 show decreased risk of grade 3-4 hematologic toxicity in two studies. These two SNPs may be important molecular biomarkers for predicting platinum-induced hematological toxicities. *XRCC1* rs25487 (G23885A, Arg399Gln) show positive association in six studies, emphasizing the potential contribution of BER pathway in oxidative stress in platinum-induced hematological toxicity, since cisplatin can exert cytotoxic effects through the generation of ROS (Zheng et al., 2020).

Apart from that, lots of genetic variants located in genes not directly linked to drug exposure are investigated. The GWAS have identified a handful of candidate genetic variants associated with platinum-based hematological toxicities and novel biologic pathways of potential impact. But these studies still face the challenge of statistically underpowered and stringent threshold of multiple testing. Additional validation in multiple independent sample sets or functional analyses are required to further elucidate the gene-phenotype relationship (Low et al., 2014).

### 4.2 Quality and inconsistency among studies

Genetic association studies require a large number of patients to provide adequate power, as a rare variant with large effect, or common variant with modest effect is more probable in genetic epidemiology (Campbell et al., 1995). While the majority of the studies in our systematic review did not indicate the sample size calculation in their statistical analysis, and the sample size in most studies in our systematic review are much smaller than that would be implied, which may be underpowered to detect a statistically significant relationship (Jorgensen and Williamson, 2008).

One of the challenges in pharmacogenomics is the ethnic background of the study population. The prevalence of toxicity varies according to the ethnic background (O’Donnell and Dolan, 2009; Li and Meyre, 2013). For example, higher rates of toxicities have been observed in east Asian populations compared to European and North American populations (Watanabe et al., 2003). In addition, allele frequency of genetic variants vary depending on the ethnic background or even the geographical location. When cases and controls are drawn from multiple ethnic or geographic groups, population stratification exists, which may put the study at risk of confounding and can lead to false positive associations (Jorgensen and Williamson, 2008). Population stratification was seldom assessed in most genetic association studies, which has been cited as a major reason for lack of replication.

Different treatment protocols are recommended according to cancer types. The composition, proportion and cycles of chemotherapy regimen can influence the incidence and degree of hematological toxicity. Furthermore, treatment protocols may also differ with regard to the use of concomitant supportive treatments. The time and dose of granulocyte colony-stimulating factor administered may be various across institutions, which are seldom mentioned in the method part and may bring confounds in genetic association studies. Moreover, there are overlap in metabolic pathway between platinum and other antineoplastic drugs, which can alter the pharmacogenetic effects of polymorphisms. For example, The ABC transporter ABCB1 and ABCC2 is responsible for the efflux of many commonly used antineoplastic drugs that usually used in combination with platinum agents, including taxanes (Marsh, 2006; Lambrechts et al., 2015). If Pt-DNA lesions are not repaired, the DNA lesion triggers activation of the apoptosis pathway, an essential step for the effectiveness of platinum-based chemotherapeutics for killing tumor cells, which is also the apoptosis pathway of many other cytotoxic drugs (Fulda and Debatin, 2006). Apart from the role of participating in DNA synthesis, MTHFR encode key enzymes for the metabolism of 5-FU (Lee et al., 2013) and RRM1 was also the primary target for gemcitabine (Yuan et al., 2015).

Genetic association studies are heavily reliant on the phenotype, but it may be difficult to establish the true phenotype. Although myelosuppression is quantitative, the degree of myelosuppression could be missed based on the frequency of measurement. Apart from that, differences in the toxicity assessment criterion and endpoints may hamper reproducibility of previous findings. Some studies analyzed the total hematological toxicity (Wang et al., 2008; Kim et al., 2009; Gu et al., 2012), while other studies analyzed the detail hematological toxicity (leukopenia, neutropenia, thrombocytopenia or anemia) (Isla et al., 2004; Tibaldi et al., 2008; Xu et al., 2012). The detail hematological toxicity may be more accurate and clinically relevant, but its low incidence requires cohort study with large sample size, which are unattainable in the current researches. Furthermore, methodological flaws were observed in the imprecise dichotomization of patients with mild toxicity and severe toxicity. The majority use the occurrence of grade 3-4 as endpoints, other studies use grade 2-4 (Isla et al., 2004; Erčulj et al., 2012; Huang et al., 2015; Jia et al., 2016; Liblab et al., 2020) or grade 1-4 (Chen et al., 2010; Erčulj et al., 2012; Deng et al., 2015; Walia et al., 2022). Medical interventions of dose reduction and/or treatment discontinuation were taken when grade 3-4 hematological toxicity arises. Therefore, the toxicity endpoints of grade 2-4 or grade 1-4 may not be clinically relevant (Supplementary Table S4).

There is statistical heterogeneity in the data analysis (Supplementary Table S5). Some studies just used chi-square test or fisher’s exact test to estimate the difference of genotype distribution in cases and controls (Kimcurran et al., 2011; Kalikaki et al., 2015). Others use logistic regression to make comparisons between groups and generate odds ratios and 95% confidence intervals. Logistic regression is more appropriate as it can provide a quantitative measure of the relationship between the groups, allow adjustment for confounding factors, and detect gene-gene or gene-environment interactions. Some studies made a clear statement about mode of inheritance assumed for analysis, and used more than one assumption (Peng et al., 2014; Bushra et al., 2020; Ferracini et al., 2021; Walia et al., 2022), while other studies only compared the three categories of genotype frequencies (homozygous wild type, heterozygous, homozygous variant) between cases and controls (Isla et al., 2004; Han et al., 2007; Kim et al., 2009; Lee et al., 2013; Liblab et al., 2020). 30 of the included studies performed the correction for multiple comparisons and only 10 studies performed validation of the results or by splitting the cohort for a primary and an exploratory analysis (Marsh et al., 2007; Qian et al., 2012; Cao et al., 2016; Gréen et al., 2016; Jia et al., 2016; Yin et al., 2016; Zheng et al., 2017; Björn et al., 2020a; Björn et al., 2020b; Svedberg et al., 2020).

### 4.3 Limitations

This review has several limitations, which mainly reflects the status of current genetic association studies. In the search process, many studies had to be excluded because hematological toxicity was not clearly described or there is no toxicity grading criterion. What’s more, we observed that the most studies did not provide toxicity statistics data (no *p*-value) or just provide insufficient toxicity data (for example, some studies only mentioned correlation in the results section without providing the original statistical data), which may raise some doubts of data authenticity. Also, some studies did not control exclusion criteria of prior history of chemotherapy and/or radiation or a considerable proportion of the patients receive chemotherapy combined with radiation or other non-platinum chemotherapy regimens, which may increase the likelihood of cumulative hematological toxicity. There is much heterogeneity between incorporated studies, thus we were unable to perform a quantitative comparison and meta-analysis.

### 4.4 Implications for clinical practice and research

Future studies should focus on the following aspects. Firstly, genetic association study inevitably faces the concerns of bias and confounding, and are susceptible to inappropriate conclusions, therefore it calls for careful planning of study design to improving quality of methodology (Saito et al., 2006). Secondly, the majority genetic polymorphisms identified in the eligible publications were repeated in only one or two studies (Hilary et al., 2024). SNPs screened out as potential factors in susceptibility to hematological toxicity in our systematic review require well-planned, methodologically robust studies to validate them. Thirdly, functional predictions of significant genetic variants needed to be confirmed and validated *in vitro* or *in vivo* work (Felipe Antonio de Oliveira et al., 2022). Fourthly, more studies should emphasize on the hematopoiesis-related pathways identified in a whole-exome sequenced study of 215 NSCLC patients treated with a single treatment (Björn et al., 2020a). Moreover, clinical trial with large sample size to perform subgroup analysis should be conducted to allow proper stratified analysis. Finally, owing to the complexity of mechanisms of platinum action, a single SNP alone may have low effect to platinum response, thus supporting a polygenetic effect in platinum‐induced hematological toxicity (Daniel et al., 2023). Future direction should be establishing appropriate statistical methods with capacity to integrate multiple genetic, phenotypic, epidemiological and clinical variables effects.

## 5 Conclusion

To summarize, this systematic review has successfully reported and evaluated studies on genetic associations of platinum-based hematological toxicities. Review of these studies identified several genetic variants that potentially affect the risk of platinum-induced hematological toxicity. Many methodological issues exist that may affect reproducibility of results and lead to inconsistency, including insufficient sample size, population stratification, various treatment schedule, heterogeneity in the assessment of hematological toxicity and statistics. Well-designed studies with sufficient samples sizes and standardization of phenotypes are warranted to address the limitations of the current studies and to ensure the robust findings that can be more effective to be used in personalized therapeutics.

## Data Availability

The original contributions presented in the study are included in the article/Supplementary Material, further inquiries can be directed to the corresponding authors.

## Abbreviations

AUC, area under curve; BER, base excision repair; CI, confidence interval; DSB, double-strand break repair; FA, fanconi anemia; FOLFOX, oxaliplatin, leucovorin and 5-fluorouracil; FR, flanking region; GWAS, genome-wide association studies; HR, homologous recombination; ICL, interstrand crosslink; MMR, mismatch repair; NCI-CTCAE, National Cancer Institute-Common Terminology Criteria for Adverse Events; NSCLC, non-small cell lung cancer; NER, nucleotide excision repair; OR, odds ratios; PRISMA, Preferred Reporting Items for Systematic Reviews and Meta-Analyses; ROS, reactive oxygen species; SNP, single nucleotide polymorphism; TLS, translesion DNA synthesis; UTR, untranslated region; WHO, World Health Organization.
