# Supplementary material for: Genetic polymorphisms and platinum-induced hematological toxicity: a systematic review
Source: Front Pharmacol. 2024 Aug 21;15:1445328. doi: 10.3389/fphar.2024.1445328 (PMC11371761; doi:10.3389/fphar.2024.1445328)
Supplement: Supplementary file 2 [file Table8.docx]

Supplementary Material

## Supplementary Table 8 Overview of genetic polymorphisms investigated once for a possible association with platinum-induced hematological toxicity

| Reference | Sample size (cancer) | Treatment | Pathway | Gene | Rs number | Association |
| --- | --- | --- | --- | --- | --- | --- |
| Sun et al.2018[57] | 1004(NSCLC) | DDP/CBP + NVB/GEM/PTX/TXT Other DDP/CBP combinations | Transporter | *SCL31A1 (CTR1)* | rs4979223, rs4978536, rs2233914, rs10817464, rs10513202, rs10759637. | *SLC31A1* rs4979223 grade 3-4 thrombocytopenia (OR = 2.60; 95% CI: 1.20-5.64; P = 0.015).  *SLC31A1* rs4978536 grade 3-4 thrombocytopenia (OR = 2.59; 95% CI: ;1.23-5.47 P = 0.012). *SLC31A1* rs10817464 grade 3-4 leucopenia (OR = 2.16; 95% CI: 1.19-3.92; P = 0.011), grade 3-4 thrombocytopenia (OR = 3.09; 95% CI: 1.20-7.93; P = 0.019) and grade 3-4 hematologic toxicity (OR = 1.98 ; 95% CI: 1.19-3.29; P = 0.008). *SLC31A1* rs10759637 grade 3-4 thrombocytopenia (OR = 2.69; 95% CI: 1.24-5.83; P = 0.012). |
| Xu et al.2012[58] | 204(NSCLC) | DDP + GEM/VP-16/TXT/VDS | Transporter | *SCL31A1 (CTR1)* | rs41280219, rs148009913, rs186968371, rs10981694, rs10981699, rs7851395, rs181949075, rs7851623,  rs12686377, rs10817465, rs10981704, rs13289462, rs10759636, rs115901881, rs7853528, rs7853810, rs2233917, rs192693896, rs1143245, rs111976327. | Negative |
| Li et al.2018[107] | 427(NSCLC) | DDP/CBP + GEM/PEM/TXT/NVB /PTX | Transporter | *ATP7A* | rs2227291, rs6622665. | Negative |
| Deng et al.2015[89] | 97(NSCLC) | DDP + GEM/NVB/PTX/TXT | Transporter | *ATP7A* | C2299G | Negative |
| Li et al.2018[107] | 427(NSCLC) | DDP/CBP + GEM/PEM/TXT/NVB /PTX | Transporter | *ATP7B* | rs1061472, rs9535826. | Negative |
| Ruzzo et al.2014[33] | 517(Colorectal cancer) | L-OHP + 5-FU + LV L-OHP + CAP | Transporter | *ABCC2(MRP2)* | rs1885301 | Negative |
| Ruzzo et al.2014[33] | 517(Colorectal cancer) | L-OHP + 5-FU + LV L-OHP + CAP | Transporter | *ABCC2(MRP2)* | rs4148386 | Negative |
| Chen et al.2010[53] | 95(NSCLC) | DDP + GEM/NVB/TXT | Transporter | *ABCB1(MDR1)* | rs3213619 | Negative |
| Chen et al.2015[108] | 317(NSCLC + SCLC) | DDP/CBP + GEM/PTX/NVB/VP-16/CPT-11 | Transporter | *ABCB1(MDR1)* | rs495714 | Negative |
| Wang et al.2021[69] | 1004(NSCLC) | DDP/CBP + NVB/GEM/PTX/TXT Other DDP/CBP combinations | Transporter | *ABCG2* | rs6857600, rs3109823, rs2725252, rs17731538, rs2231138, rs11931123, rs1871744, rs2231146, rs12505410, rs4148157, rs2231164, rs2231137, rs2231142. | *ABCG2* rs12505410: grade 3-4 neutropenia (CC vs. AA OR = 2.29; 95% CI: 1.28-4.10; P = 0.005, CC vs. AA + AC OR = 2.09; 95% CI: 1.23-3.57; P = 0.007). *ABCG2* rs1871744: grade 3-4 anemia (AG vs. AA OR = 0.37; 95% CI: 0.14-0.95; P = 0.039, AG + GG vs. AA OR = 0.42; 95% CI: 0.19-0.95; P = 0.038). *ABCG2* rs2231138: grade 3-4 anemia (AG vs. AA OR = 2.57; 95% CI: 1.12-5.89; P = 0.026, AG vs. AA + GG OR = 2.60; 95% CI: 1.13-5.94; P = 0.024). |
| Chen et al.2015[108] | 317(NSCLC + SCLC) | DDP/CBP + GEM/PTX/NVB/VP-16/CPT-11 | Transporter | *ABCG2* | rs1448784 | Negative |
| Chen et al.2015[108] | 317(NSCLC + SCLC) | DDP/CBP + GEM/PTX/NVB/VP-16/CPT-11 | Transporter | *OCT2* | rs1869641, rs1883306, rs195854, rs195862, rs2444933, rs3823036. | Negative |
| Qian et al.2016[63] | 403(NSCLC) | DDP/CBP + GEM/PEM/PTX/TXT/NVB | Transporter | *OCT2* | rs316003 | Negative |
| Chen et al.2015[108] | 317(NSCLC + SCLC) | DDP/CBP + GEM/PTX/NVB/VP-16/CPT-11 | Transporter | *SIRT1* | rs12778366, rs3740051, rs3758391, rs4746720. | Negative |
| Chen et al.2015[108] | 317(NSCLC + SCLC) | DDP/CBP + GEM/PTX/NVB/VP-16/CPT-11 | Transporter | *SLC2A1* | rs1385129, rs3738514, rs3806400, rs3820589, rs4658, rs841844. | Negative |
| Chen et al.2015[108] | 317(NSCLC + SCLC) | DDP/CBP + GEM/PTX/NVB/VP-16/CPT-11 | Transporter | *TMEM205* | rs172731, rs7251786, rs896412. | Negative |
| Chen et al.2015[108] | 317(NSCLC + SCLC) | DDP/CBP + GEM/PTX/NVB/VP-16/CPT-11 | Transporter | *AQP2* | rs10875989, rs296766, rs3759125, rs3759126, rs461872, rs7305534, rs7314734. | *AQP2* rs10875989: grade 3-4 hematologic toxicity in additive model (OR = 1.53; 95% CI: 1.06-2.21; P = 0.025) and dominant model (OR = 1.83; 95% CI: 1.03-2.07; P = 0.039). |
| Chen et al.2015[108] | 317(NSCLC + SCLC) | DDP/CBP + GEM/PTX/NVB/VP-16/CPT-11 | Transporter | *AQP9* | rs1516400, rs1554203, rs1867380, rs2077737, rs8023369, rs9920375 | Negative |
| Senk et al.2019[50] | 194(Malignant mesothelioma) | DDP + GEM/PEM | Transporter | *AQP1* | rs1049305 | *AQP1* rs1049305: grade 2-4 anemia (GC vs. GG OR = 0.46; 95% CI: 0.23-0.92; P = 0.029), grade 2-4 anemia (GC + CC vs. GG OR = 0.52; 95% CI: 0.27-0.99; P = 0.046) and grade 2-4 leukopenia (CC vs. GG OR = 3.03; 95% CI: 1.10-8.38; P = 0.033, GC + CC vs. GG OR = 2.09; 95% CI: 1.00-4.35; P = 0.049). |
| Senk et al.2019[50] | 194(Malignant mesothelioma) | DDP + GEM/PEM | Transporter | *AQP1* | rs1476597 | Negative |
| Senk et al.2019[50] | 194(Malignant mesothelioma) | DDP + GEM/PEM | Transporter | *AQP1* | rs28362731 | *AQP1* rs28362731: grade 1-4 thrombocytopenia (GA vs. GG OR = 4.63; 95% CI: 1.13-19.05; P = 0.034). |
| Lee et al.2013[37] | 292(Colon cancer) | L-OHP + LV + 5-FU | Metabolism | *AGXT* | rs34116584 | Negative |
| Lee et al.2013[37] | 292(Colon cancer) | L-OHP + LV + 5-FU | Metabolism | *AGXT* | rs4426527 | Negative |
| Khrunin et al.2012[40] | 104(Ovarian cancer) | DDP + CTX | Metabolism | *CYP2E1* | 96-bp insertion | Negative |
| Khrunin et al.2012[40] | 104(Ovarian cancer) | DDP + CTX | Metabolism | *CYP2E1* | 1053C＞T | Negative |
| Khrunin et al.2012[40] | 104(Ovarian cancer) | DDP + CTX | Metabolism | *CYP2E1* | 7632T＞A | Negative |
| Khrunin et al.2012[40] | 104(Ovarian cancer) | DDP + CTX | Metabolism | *CYP2E1* | 9896C＞G | Negative |
| Khrunin et al.2012[40] | 104(Ovarian cancer) | DDP + CTX | Metabolism | *GSTA1* | rs3957357 | Negative |
| Khrunin et al.2012[40] | 104(Ovarian cancer) | DDP + CTX | Metabolism | *GSTM3* | AGG deletion | GSTM3 AGG deletion: grade 1-4 thrombocytopenia (AGG/AGG vs. AGG/− + −/− OR = 0.23; 95% CI: 0.07-0.71; P = 0.014) and grade 2-4 anemia (AGG/AGG vs. AGG/− + −/− OR = 0.21; 95% CI: 0.07-0.69; P = 0.007). |
| Khrunin et al.2012[40] | 104(Ovarian cancer) | DDP + CTX | Metabolism | *GSTM3* | rs7483 | Negative |
| Marsh et al.2007[29] | 914 Discovery cohort and validation cohort in ratio 2:1(Ovarian cancer) | CBP + PTX/TXT | Metabolism | *MAPT* | P587P | Negative |
| Marsh et al.2007[29] | 914 Discovery cohort and validation cohort in ratio 2:1(Ovarian cancer) | CBP + PTX/TXT | Metabolism | *MPO* | -463G＞A | Negative |
| Chen et al.2015[108] | 317(NSCLC + SCLC) | DDP/CBP + GEM/PTX/NVB/VP-16/CPT-11 | NER | *HMGB2* | rs6832850, rs7686909. | Negative |
| Zheng et al.2017[46] | 437 in the in a discovery cohort and 781 in the validation cohort (NSCLC) | DDP/CBP + NVB/GEM/PTX/TXT/PEM | NER | *RAD23B* | rs1805329 | Negative |
| Chen et al.2015[108] | 317(NSCLC + SCLC) | DDP/CBP + GEM/PTX/NVB/VP-16/CPT-11 | NER | *RPA1* | rs1131636, rs12727,  rs17292622, rs17339382, rs17339395, rs17734, rs3744766, rs3744767, rs3744768, rs3744769, rs5030740, rs9082,  rs9914073. | Negative |
| Chen et al.2015[108] | 317(NSCLC + SCLC) | DDP/CBP + GEM/PTX/NVB/VP-16/CPT-11 | NER | *SSRP1* | rs10896607 | Negative |
| Chen et al.2015[108] | 317(NSCLC + SCLC) | DDP/CBP + GEM/PTX/NVB/VP-16/CPT-11 | NER | *XPA* | rs3176751, rs3176752,  rs7539638. | Negative |
| Chen et al.2015[108] | 317(NSCLC + SCLC) | DDP/CBP + GEM/PTX/NVB/VP-16/CPT-11 | NER | *XRCC5* | rs1051677, rs1051685,  rs2440, rs6941. | *XRCC5* rs1051685: grade 3-4 hematologic toxicity in additive model (OR = 0.37; 95% CI: 0.14-0.96; P = 0.041) and dominant model (OR = 0.37; 95% CI: 0.14-0.97; P = 0.043). XRCC5 rs6941: grade 3-4 hematologic toxicity in additive model (OR = 1.93; 95% CI: 1.21-3.10; P = 0.006) and dominant model (OR = 2.05; 95% CI: 1.19-3.53; P = 0.001). |
| Song et al.2016[81] | 1004(NSCLC+SCLC) | DDP/CBP + NVB/GEM/PTX/TXT Other DDP/CBP combinations | NER | *XPC, RAD23B, ERCC2, GTF2H1, XPA, ERCC5, ERCC1, ERCC4, ERCC8, ERCC6, DDB2, LIG1, CDK7, CCNH, MNAT1, RPA1, RPA2, RFC1, RFC2, POLD1, POLD2, POLD3, POLD4, POLE, POLE2, GTF2H3, GTF2H4* | 173 SNPs in 27 genes | No SNPs satisfied the significant level of bonferroni correction *GTF2H1* rs4150558: grade 3-4 anemia (OR = 2.74; 95% CI: 1.23-6.09; P = 0.013). *POLD3* rs10857: grade 3-4 neutropenia (OR = 0.55; 95% CI: 0.39-0.76; P = 3.01×10−4). *POLD3* rs6592576: grade 3-4 neutropenia (OR = 0.56; 95% CI: 0.41-0.77; P = 3.58×10−4). *RPA1* rs12727: grade 3-4 thrombocytopenia (OR = 1.81; 95% CI: 1.02-3.21; P = 0.044). *POLD1* rs3219281: grade 3-4 thrombocytopenia (OR = 1.87; 95% CI: 1.4-3.34; P = 0.035). *POLD1* rs3219341: grade 3-4 thrombocytopenia (OR = 1.84; 95% CI: 1.03-3.26; P = 0.039). *POLD1* rs1726801: grade 3-4 thrombocytopenia (OR = 1.86; 95% CI: 1.05-3.30; P = 0.033). |
| Peng et al.2014[88] | 235(NSCLC) | DDP + PTX/GEM/NVB/PEM | BER | *APE1* | rs1760944 | Negative |
| Zheng et al.2017[46] | 437 in the in a discovery cohort and 781 in the validation cohort (NSCLC) | DDP/CBP + NVB/GEM/PTX/TXT/PEM | BER | *APE1* | rs2307486 | Negative |
| Zheng et al.2017[46] | 437 in the in a discovery cohort and 781 in the validation cohort (NSCLC) | DDP/CBP + NVB/GEM/PTX/TXT/PEM | BER | *PARP1* | rs1136410 | Negative |
| Zheng et al.2017[46] | 437 in the in a discovery cohort and 781 in the validation cohort (NSCLC) | DDP/CBP + NVB/GEM/PTX/TXT/PEM | BER | *OGG1* | rs1052133 | Negative |
| Zheng et al.2017[46] | 437 in the in a discovery cohort and 781 in the validation cohort (NSCLC) | DDP/CBP + NVB/GEM/PTX/TXT/PEM | BER | *MUTYH* | rs3219489 | Negative |
| Zheng et al.2017[46] | 437 in the in a discovery cohort and 781 in the validation cohort (NSCLC) | DDP/CBP + NVB/GEM/PTX/TXT/PEM | BER | *ATM* | rs189037, rs4585, rs228589. | Negative |
| Zheng et al.2017[46] | 437 in the in a discovery cohort and 781 in the validation cohort (NSCLC) | DDP/CBP + NVB/GEM/PTX/TXT/PEM | BER | *MDB4* | rs10342, rs140693. | Negative |
| Zheng et al.2017[46] | 437 in the in a discovery cohort and 781 in the validation cohort (NSCLC) | DDP/CBP + NVB/GEM/PTX/TXT/PEM | BER | *EXO1* | rs1047840, rs1776148,  rs735943. | Negative |
| Zheng et al.2017[46] | 437 in the in a discovery cohort and 781 in the validation cohort (NSCLC) | DDP/CBP + NVB/GEM/PTX/TXT/PEM | BER | *POLQ* | rs3218649 | Negative |
| Liu et al.2017[129] | 220(NSCLC+SCLC) | DDP/CBP + GEM/PEM/PTX/TXT/NVB | MMR | *MLH1* | rs10849, rs1540354,  rs749072. | Negative |
| Liu et al.2017[129] | 220(NSCLC+SCLC) | DDP/CBP + GEM/PEM/PTX/TXT/NVB | MMR | *MSH2* | rs10191478, rs12999145 rs13019654, rs1981929, rs2303428, rs4608577, rs4952887, rs6544991, rs7602094. | Negative |
| Liu et al.2017[129] | 220(NSCLC+SCLC) | DDP/CBP + GEM/PEM/PTX/TXT/NVB | MMR | *MSH3* | rs245340, rs245346, rs26778, rs26784, rs3816729, rs6151627, rs6151670, rs6151892, rs6151914, rs7709909. | *MSH3* rs6151627: grade 3-4 hematologic toxicity in dominant model (OR = 2.38; 95% CI: 1.23-4.60; P = 0.010).  *MSH3* rs6151670: grade 3-4 hematologic toxicity in dominant model (OR = 2.05; 95% CI: 1.07-3.93; P = 0.031).  *MSH3* rs7709909: grade 3-4 hematologic toxicity in dominant model (OR = 2.38; 95% CI: 1.23-4.64; P = 0.010). |
| Liu et al.2017[129] | 220(NSCLC+SCLC) | DDP/CBP + GEM/PEM/PTX/TXT/NVB | MMR | *MSH4* | rs3806162, rs5745532. | Negative |
| Liu et al.2017[129] | 220(NSCLC+SCLC) | DDP/CBP + GEM/PEM/PTX/TXT/NVB | MMR | *MSH5* | rs3117572, rs409558, rs707937, rs707938, rs707939, rs805304. | *MSH5* rs805304: grade 3-4 hematologic toxicity in dominant model (OR = 1.99; 95% CI: 1.01-3.90; P = 0.047). |
| Liu et al.2017[129] | 220(NSCLC+SCLC) | DDP/CBP + GEM/PEM/PTX/TXT/NVB | MMR | *MSH6* | rs2020910, rs2348244, rs2710163, rs3136329, rs3732190, rs6713506, rs6742522. | Negative |
| Zheng et al.2017[46] | 437 in the in a discovery cohort and 781 in the validation cohort  (NSCLC) | DDP/CBP + NVB/GEM/PTX/TXT/PEM | MMR | *hMLHl* | rs1800734 | Negative |
| Zheng et al.2017[46] | 437 in the in a discovery cohort and 781 in the validation cohort  (NSCLC) | DDP/CBP + NVB/GEM/PTX/TXT/PEM | MMR | *hMSH2* | rs2303425, rs2303428. | Negative |
| Zheng et al.2017[46] | 437 in the in a discovery cohort and 781 in the validation cohort (NSCLC) | DDP/CBP + NVB/GEM/PTX/TXT/PEM | MMR | *hMSH3* | rs1650697, rs26279. | Negative |
| Zheng et al.2017[46] | 437 in the in a discovery cohort and 781 in the validation cohort (NSCLC) | DDP/CBP + NVB/GEM/PTX/TXT/PEM | MMR | *MLH3* | rs175080 | Negative |
| Zheng et al.2017[46] | 437 in the in a discovery cohort and 781 in the validation cohort  (NSCLC) | DDP/CBP + NVB/GEM/PTX/TXT/PEM | MMR | *MSH5* | rs2075789 | Negative |
| Zheng et al.2017[46] | 437 in the in a discovery cohort and 781 in the validation cohort  (NSCLC) | DDP/CBP + NVB/GEM/PTX/TXT/PEM | MMR | *hMSH6* | rs1042821 | Negative |
| Zheng et al.2017[46] | 437 in the in a discovery cohort and 781 in the validation cohort (NSCLC) | DDP/CBP + NVB/GEM/PTX/TXT/PEM | MMR | *PMS1* | rs5742933 | Negative |
| Zheng et al.2017[46] | 437 in the in a discovery cohort and 781 in the validation cohort (NSCLC) | DDP/CBP + NVB/GEM/PTX/TXT/PEM | MMR | *PMS2* | rs1062372, rs2228006, rs17420802. | Negative |
| Shao et al.2014[130] | 663(NSCLC) | DDP/CBP + NVB/GEM/PTX/TXT Other DDP/CBP combinations | TLS | *POLK* | rs3213801, rs5744533, rs1018119, rs4604177, rs10077427, rs5744545, rs3756558, rs449106,  rs5744653, rs5744651,  rs5744655. | *POLK* rs3756558: grade 3-4 hematological toxicity in additive model (OR = 0.71; 95% CI: 0.50-0.98; P = 0.042). |
| Chu et al.2016[97] | 1021(NSCLC) | DDP/CBP + NVB/GEM/PTX /TXT Other DDP/CBP combinations | TLS | *Rad18* | rs615967, rs588232,  rs586014, rs654448,  rs6763823, rs9880051,  rs618784, rs686195,  rs669906. | *RAD18* rs586014: grade 3-4 hematological toxicity in non-smoker (AG + GG vs. AA OR = 0.48; 95% CI: 0.26-0.88; P = 0.015). *RAD18* rs654448: grade 3-4 hematological toxicity in non-smoker (AA + AG vs. GG OR = 2.13; 95% CI: 1.05-4.27; P = 0.033). *RAD18* rs9880051: grade 3-4 hematological toxicity in non-smoker (AA + AG vs. GG OR = 1.95; 95% CI: 1.11-3.49; P = 0.021). *RAD18* rs6763823: grade 3-4 leukocytopenia toxicity in smoker (AA + AG vs. GG OR = 0.60; 95% CI: 0.38-0.95; P = 0.031). *RAD18* rs9880051: grade 3-4 leukocytopenia toxicity in smoker (AA + AG vs. GG OR = 0.39; 95% CI: 0.22-0.70; P = 0.002). |
| Goričar et al.2013[49] | 139(Malignant mesothelioma) | DDP + GEM/PEM Other DDP doublets | TLS | *REV1* | rs3087399, rs3087403. | *REV1* rs3087386: grade 2-4 neutropenia (GA+AA vs. GG OR = 0.38; 95% CI: 0.17-0.84; P = 0.017). |
| Goričar et al.2013[49] | 139(Malignant mesothelioma) | DDP + GEM/PEM Other DDP doublets | TLS | *REV3L* | rs3204953, rs455732, | Negative |
| Ye et al.2015[96] | 663(NSCLC) | DDP/CBP + NVB/GEM/PTX/TXT Other DDP/CBP combinations | TLS | *REV3* | rs240966, rs240969,  rs9487643, rs4945880, rs3218573, rs456865,  rs3218606, rs17510346. | *REV3* rs240966: grade 3-4 hematologic toxicity (OR = 0.44; 95% CI: 0.21-0.94; P = 0.03). *REV3* rs4945880: grade 3-4 hematologic toxicity (OR = 1.50; 95% CI: 1.05-2.15; P = 0.025). |
| Ye et al.2015[96] | 663(NSCLC) | DDP/CBP + NVB/GEM/PTX/TXT Other DDP/CBP combinations | TLS | *REV7* | rs2336030, rs2233025, | *REV7* rs2233025: grade 3-4 hematologic toxicity (OR = 0.29; 95% CI: 0.10-0.82; P = 0.018). |
| Zheng et al.2017[46] | 437 in the in a discovery cohort and 781 in the validation cohort (NSCLC) | DDP/CBP + NVB/GEM/PTX/TXT/PEM | TLS | *POLH* | rs6941583 | Negative |
| Zheng et al.2017[46] | 437 in the in a discovery cohort and 781 in the validation cohort (NSCLC) | DDP/CBP + NVB/GEM/PTX/TXT/PEM | TLS | *REV7* | rs2233004 | Negative |
| Zheng et al.2017[46] | 437 in the in a discovery cohort and 781 in the validation cohort  (NSCLC) | DDP/CBP + NVB/GEM/PTX/TXT/PEM | TLS | *Rad18* | rs615961 | Negative |
| Zheng et al.2017[46] | 437 in the in a discovery cohort and 781 in the validation cohort  (NSCLC) | DDP/CBP + NVB/GEM/PTX/TXT/PEM | DSB | *BRCA1* | rs799917 | Negative |
| Zheng et al.2017[46] | 437 in the in a discovery cohort and 781 in the validation cohort (NSCLC) | DDP/CBP + NVB/GEM/PTX/TXT/PEM | DSB | *RAD52* | rs7963551,  rs1051669. | Negative |
| Zheng et al.2017[46] | 437 in the in a discovery cohort and 781 in the validation cohort (NSCLC) | DDP/CBP + NVB/GEM/PTX/TXT/PEM | DSB | *XRCC2* | rs6464268 | Negative |
| Zheng et al.2017[46] | 437 in the in a discovery cohort and 781 in the validation cohort (NSCLC) | DDP/CBP + NVB/GEM/PTX/TXT/PEM | DSB | *RAD51* | rs1801321, rs1801320, rs4417527, rs12593359. | In discovery corhort, *RAD51* rs1801320: grade 3-4 hematologic toxicity in additive model (OR = 0.552; 95% CI: 0.330–0.924; P = 0.024) *RAD51* rs12593359 grade 3-4 leukocytopenia in dominant model (OR = 0.434; 95% CI: 0.199–0.949; P = 0.037) |
| Zheng et al.2017[46] | 437 in the in a discovery cohort and 781 in the validation cohort (NSCLC) | DDP/CBP + NVB/GEM/PTX/TXT/PEM | DSB | *MRE11A* | rs11020802 | Negative |
| Zheng et al.2017[46] | 437 in the in a discovery cohort and 781 in the validation cohort (NSCLC) | DDP/CBP + NVB/GEM/PTX/TXT/PEM | DSB | *NBS1* | rs1805794, rs13312840, rs2735383. | Negative |
| Zheng et al.2017[46] | 437 in the in a discovery cohort and 781 in the validation cohort (NSCLC) | DDP/CBP + NVB/GEM/PTX/TXT/PEM | DSB | *XRCC3* | rs1799794 | Negative |
| Zheng et al.2017[46] | 437 in the in a discovery cohort and 781 in the validation cohort (NSCLC) | DDP/CBP + NVB/GEM/PTX/TXT/PEM | DSB | *WRN* | rs2230009, rs1801195. | Negative |
| Zheng et al.2017[46] | 437 in the in a discovery cohort and 781 in the validation cohort (NSCLC) | DDP/CBP + NVB/GEM/PTX/TXT/PEM | FA | *FANCA* | rs2239359, rs11646374, rs9282681. | Negative |
| Zheng et al.2017[46] | 437 in the in a discovery cohort and 781 in the validation cohort (NSCLC) | DDP/CBP + NVB/GEM/PTX/TXT/PEM | FA | *FANCB* | rs2185383 | Negative |
| Zheng et al.2017[46] | 437 in the in a discovery cohort and 781 in the validation cohort (NSCLC) | DDP/CBP + NVB/GEM/PTX/TXT/PEM | FA | *FANCC* | rs4647554 | Negative |
| Zheng et al.2017[46] | 437 in the in a discovery cohort and 781 in the validation cohort (NSCLC) | DDP/CBP + NVB/GEM/PTX/TXT/PEM | FA | *FANCD1* | rs206118, rs543304. | Negative |
| Zheng et al.2017[46] | 437 in the in a discovery cohort and 781 in the validation cohort (NSCLC) | DDP/CBP + NVB/GEM/PTX/TXT/PEM | FA | *FANCD2* | rs2272125 | Negative |
| Zheng et al.2017[46] | 437 in the in a discovery cohort and 781 in the validation cohort (NSCLC) | DDP/CBP + NVB/GEM/PTX/TXT/PEM | FA | *FANCE* | rs9462088, rs3823434,  rs6907678. | Negative |
| Zheng et al.2017[46] | 437 in the in a discovery cohort and 781 in the validation cohort (NSCLC) | DDP/CBP + NVB/GEM/PTX/TXT/PEM | FA | *FANCF* | rs3740615, rs4447177,  rs4442551. | Negative |
| Zheng et al.2017[46] | 437 in the in a discovery cohort and 781 in the validation cohort (NSCLC) | DDP/CBP + NVB/GEM/PTX/TXT/PEM | FA | *FANCJ* | rs4986765, rs4986764, rs2048718, rs11079454. | Negative |
| Zheng et al.2017[46] | 437 in the in a discovery cohort and 781 in the validation cohort (NSCLC) | DDP/CBP + NVB/GEM/PTX/TXT/PEM | FA | *FANCM* | rs3776332 | Negative |
| Qian et al.2015[109] | 663(NSCLC) | DDP/CBP + NVB/GEM/PTX/TXT Other DDP/CBP combinations | Apoptosis | *MDM2* | rs1470383, rs1690924, rs3730488, rs3730581, rs3730635. | *MDM2* rs1470383: grade 3-4 hematologic toxicity (GG vs. AA OR = 4.10; 95% CI: 1.73-9.71; P = 0.001, GG vs. AG + AA OR = 4.08; 95% CI: 1.73-9.58; P = 0.001). |
| Zheng et al.2014[110] | 444(NSCLC) | DDP/CBP + GEM /VP-16 /PTX /PEM  Other DDP/CBP combinations | Apoptosis | *MDM2* | rs937282 | *MDM2* rs937282: grade 3-4 hematologic toxicity (CG vs. GG OR = 1.121; 95% CI: 1.062-1.184; P = 0.026). |
| Khrunin et al.2012[40] | 104(Ovarian cancer) | DDP + CTX | Apoptosis | *TP53* | 16bp duplication | Negative |
| Khrunin et al.2012[40] | 104(Ovarian cancer) | DDP + CTX | Apoptosis | *TP53* | 13494G＞A | Negative |
| Gu et al.2012[115] | 445(NSCLC) | DDP/CBP + NVB/GEM/PTX /TXT  Other DDP/CBP combinations | Apoptosis | *BAX* | rs4645878 | Negative |
| Gu et al.2012[115] | 445(NSCLC) | DDP/CBP + NVB/GEM/PTX /TXT  Other DDP/CBP combinations | Apoptosis | *BCL2* | rs1564483 | Negative |
| Gu et al.2012[115] | 445(NSCLC) | DDP/CBP + NVB/GEM/PTX /TXT  Other DDP/CBP combinations | Apoptosis | *BCL2* | rs1801018 | Negative |
| Gu et al.2012[115] | 445(NSCLC) | DDP/CBP + NVB/GEM/PTX /TXT  Other DDP/CBP combinations | Apoptosis | *BCL2* | rs2279115 | Negative |
| Qian et al.2012[44] | 279 in a discovery set 384 in a validation set (NSCLC) | DDP/CBP + NVB/GEM/PTX/TXT Other DDP/CBP combination | Apoptosis | *CASP10* | rs10190756, rs11674246, rs3731714 | Negative |
| Gu et al.2012[115] | 445(NSCLC) | DDP/CBP + NVB/GEM/PTX /TXT  Other DDP/CBP combinations | Apoptosis | *CASP10* | rs13006529 | Negative |
| Gu et al.2012[115] | 445(NSCLC) | DDP/CBP + NVB/GEM/PTX /TXT  Other DDP/CBP combinations | Apoptosis | *CASP10* | rs3900115 | Negative |
| Gu et al.2012[115] | 445(NSCLC) | DDP/CBP + NVB/GEM/PTX /TXT  Other DDP/CBP combinations | Apoptosis | *CASP3* | rs6948 | *CASP3* rs6948: grade 3-4 hematologic toxicity (AC + CC vs. AA OR = 0.524; 95% CI: 0.333-0.824; P = 0.005). |
| Qian et al.2012[44] | 279 in a discovery set 384 in a validation set (NSCLC) | DDP/CBP + NVB/GEM/PTX/TXT Other DDP/CBP combination | Apoptosis | *CASP8* | rs10192461, rs1045494, rs12990906, rs3754935, rs3769821, rs3769825, rs3769827, rs6743518, rs7608692. | *CASP8* rs12990906: grade 3-4 hematologic toxicity (GG vs. AA OR = 0.45; 95% CI: 0.26-0.78; P = 0.004, AG + GG vs. AA OR = 0.64; 95% CI: 0.44-0.94; P = 0.023) |
| Liu et al.2017[116] | 555(Lung adenocarcinoma) | DDP/CBP + PTX/TXT/NVB /VP-16 /BEV | Apoptosis | *CASP8* | rs3769827, rs7608692, rs3769825, rs12990906, rs3754935, rs3769821,  rs1045494. | *CASP8* rs7608692: grade 3-4 neutropenia in additive model (OR = 0.52; 95% CI: 0.32-0.85; P = 0.009). |
| Gu et al.2012[115] | 445(NSCLC) | DDP/CBP + NVB/GEM/PTX /TXT  Other DDP/CBP combinations | Apoptosis | *CASP8* | rs3834129 | Negative |
| Qian et al.2015[109] | 663(NSCLC) | DDP/CBP + NVB/GEM/PTX/TXT Other DDP/CBP combinations | Apoptosis | *MDM2* | rs1470383, rs1690924, rs3730488, rs3730581, rs3730635. | *MDM2* rs1470383: grade 3-4 hematologic toxicity (GG vs. AA OR = 4.10; 95% CI: 1.73-9.71; P = 0.001, GG vs. AG + AA OR, 4.08; 95% CI: 1.73-9.58; P = 0.001). |
| Zheng et al.2014[110] | 444(NSCLC) | DDP/CBP + GEM /VP-16 /PTX /PEM  Other DDP/CBP combinations | Apoptosis | *MDM2* | rs937282 | *MDM2* rs937282: grade 3-4 hematologic toxicity (CG vs. GG OR = 1.121; 95% CI: 1.062-1.184; P = 0.026). |
| Gu et al.2012[115] | 445(NSCLC) | DDP/CBP + NVB/GEM/PTX /TXT  Other DDP/CBP combinations | Apoptosis | *MIF* | rs755622 | Negative |
| Gu et al.2012[115] | 445(NSCLC) | DDP/CBP + NVB/GEM/PTX /TXT  Other DDP/CBP combinations | Apoptosis | *TNFα* | rs1800629 | Negative |
| Zheng et al.2021[117] | 437(NSCLC) | DDP/CBP + NVB/GEM/PTX/TXT/PEM | Others | *EPO* | rs1617640 | *EPO* rs1617640: grade 3-4 hematologic toxicity (TT vs. GG + GT OR = 1.783; 95% CI: 1.098-2.898; P = 0.019, TT vs. GG OR = 4.702; 95% CI: 1.048-21.095; P = 0.043). |
| Peng et al.2013[118] | 663(NSCLC) | DDP/CBP + NVB/GEM/PTX/TXT Other DDP/CBP combinations | Others ATPases | *VCP* | rs1053318, rs2074549, rs514492. | *VCP* rs2074549: grade 3-4 neutropenia (OR = 2.863; 95% CI: 1.564-5.241; P = 0.001). |
| Gong et al.2019[119] | 467(NSCLC + SCLC) | DDP/CBP + PEM/GEM/PTX /TXT/VP-16 other DDP/CBP-based chemotherapy (CPT-11 + DDP/CBP, NVB +DDP/CBP) | Others act as an oncogene that prevents cell-cycle arrest and cell death | *STAT3* | rs4796793 | *STAT3* rs4796793: grade 3-4 hematological toxicity in additive model (OR = 1.352; 95% CI: 1.001-1.826; P = 0.049). |
| Chen et al.2014[120] | 412(NSCLC + SCLC) | DDP/CBP + GEM/PTX/NVB/VP-16/CPT-11 | Others belongs to The Wnt signaling pathway regulates the transcription of many genes that are involved in cell proliferation, differentiation | *WISP1* | rs10956696, rs10956697, rs11778573, rs16893344, rs16904853, rs2013146, rs2929946, rs2929965, rs2929969, rs2929970, rs2929973, rs2929986, rs2977519, rs2977529, rs2977530, rs2977536, rs2977537, rs2977549, rs2977551, rs3739262, rs4330674, rs62514003, rs62514004, rs72731505, rs72731507, rs754958,  rs7828685, rs7843546. | *WISP1* rs16904853: grade 3-4 hematologic toxicity in recessive model (OR = 0.45; 95% CI: 0.23-0.89; P = 0.021). *WISP1* rs2929970: grade 3-4 hematologic toxicity in recessive model (OR = 0.34; 95% CI: 0.13-0.89; P = 0.028). *WISP1* rs2977549: grade 3-4 hematologic toxicity in recessive model (OR = 0.39; 95% CI: 0.17-0.88; P = 0.024)  *WISP1* rs2977551: grade 3-4 hematologic toxicity in recessive model (OR = 0.43; 95% CI: 0.19-0.99; P = 0.048) |
| Zhao et al.2014[121] | 1004(NSCLC) | DDP/CBP + NVB/GEM/PTX/TXT Other DDP/CBP combinations | Others Cancer initiation and progression | *TERT* | rs2736118, rs2075786, rs4975605, rs2736100, rs2853676, rs2736098,  rs2736109. | Negative |
| Xu et al.2016[122] | 272 female patients (NSCLC) | DDP/CBP + NVB/GEM/PTX/TXT Other DDP/CBP combinations | Others G2/M checkpoint kinase | *CHEK2* | rs2236141, rs2236142, rs4035540, rs5762746. | Negative |
| Zhao et al.2011[123] | 663(NSCLC) | DDP/CBP + NVB/GEM/PTX/TXT Other DDP/CBP combinations | Others Mediator of extracellular matrix (ECM) degradation | *MMP-2* | 16 tag SNPs  rs1477017, rs865094, rs11076101, rs17301608, rs12934241, rs2241146, rs9928731, rs243849, rs243847, rs243844, rs243843, rs1992116, rs2287075, rs11639960, rs243836, rs7201. | *MMP-2* rs1477017: grade 3-4 neutropenia in additive model (OR = 1.53; 95% CI: 1.08-2.15; P = 0.016). *MMP-2* rs17301608: grade 3-4 neutropenia in recessive model (OR = 2.11; 95% CI: 1.21-3.69; P=0.009). *MMP-2* rs12934241: grade 3-4 neutropenia in recessive model (OR = 8.08; 95% CI: 2.83-23.06; P = 9.5*10-5c). *MMP-2* rs243847: grade 3-4 neutropenia in additive model (OR = 0.67; 95% CI: 0.47-0.95; P = 0.026). *MMP-2* rs243844: grade 3-4 neutropenia in additive model (OR = 0.67; 95% CI: 0.46-0.97; P = 0.036). *MMP-2* rs11639960: grade 3-4 neutropenia in additive model (OR = 1.47; 95% CI: 1.05-2.07; P = 0.026). *MMP-2* rs1992116: grade 3-4 hematologic toxicity in additive model (OR = 1.40; 95% CI: 1.06-1.85; P = 0.017), grade 3-4 neutropenia in additive model (OR = 1.46; 95% CI: 1.02-2.09; P = 0.040), and grade 3-4 anemia in additive model (OR = 3.11; 95% CI: 1.48-6.50; P = 0.003). |
| Wang et al.2016[124] | 1004(NSCLC) | DDP/CBP + NVB/GEM/PTX/TXT Other DDP/CBP combinations | Others PI3K/PTEN/AKT/mTOR | *RICTOR* | 10 tag SNPs | *RICTOR* rs7703002 : grade 3-4 anemia (AC vs. CC OR = 2.55; 95% CI: 1.17–5.54; P = 0.018, AA + AC vs. CC OR = 2.45; 95% CI: 1.16–5.30; P = 0.020). *RICTOR* rs4321771: grade 3-4 thrombocytopenia (AG vs GG OR = 2.84; 95% CI: 1.25-6.46; P = 0.013, AA + GA vs GG OR = 2.75; 95% CI: 1.21-6.25; P = 0.016). |
| Yin et al.2015[125] | 325(NSCLC) | DDP/CBP + GEM /VP-16 /PTX /PEM  Other DDP/CBP combinations | Others Translation initiation | *eIF3a* | rs1409314, rs4752219, rs4752220, rs7091672, rs10510050, rs10886342, rs11198804, rs2275112, rs10787899, rs4752269. | *eIF3a* rs1409314, rs4752219, rs4752220 and rs7091672 were significantly correlated with the toxicities tested (neutropenia, anemia, thrombocytopenia) |
| Zou et al.2016[126] | 317(NSCLC+SCLC) | DDP/CBP + GEM/PTX/NVB/VP-16/CPT-11 | Others mediators or modulators of important cellular activities, | *HSPA4* | rs3088225, rs4616886. | *RAC1* rs836554: grade 3-4 hematologic toxicity in age >55 subgroup in recessive model (OR = 3.32; 95% CI: 1.23-8.99; P = 0.018) and grade 3-4 hematologic toxicity in NSCLC subgroup in recessive model (OR = 3.50; 95% CI: 1.37-8.99; P = 0.009). *RAC1* rs4720672: grade 3-4 hematologic toxicity in age ≤55 subgroup in dominant model (OR = 2.73; 95% CI: 1.15-6.47; P = 0.023). *RAC1* rs12536544: grade 3-4 hematologic toxicity in female subgroup in additive model (OR = 0.24; 95% CI: 0.07-0.78; P = 0.018) and in dominant model (OR = 0.14; 95% CI: 0.04-0.57; P = 0.006) in NSCLC subgroup in additive model (OR = 0.56; 95% CI: 0.33-0.95; P = 0.032) in non-smoker subgroup in dominant model (OR = 0.38; 95% CI: 0.15-0.96; P = 0.041). |
| Zou et al.2016[126] | 317(NSCLC+SCLC) | DDP/CBP + GEM/PTX/NVB/VP-16/CPT-11 | Others mediators or modulators of important cellular activities, | *HSPB1* | rs2009836, rs2070804, rs2868370, rs2868371,  rs7459185. | Negative |
| Zou et al.2016[126] | 317(NSCLC+SCLC) | DDP/CBP + GEM/PTX/NVB/VP-16/CPT-11 | Others mediators or modulators of important cellular activities, | *HSPE1* | rs13386066, rs17730989, rs2605039. | Negative |
| Zou et al.2016[126] | 317(NSCLC+SCLC) | DDP/CBP + GEM/PTX/NVB/VP-16/CPT-11 | Others mediators or modulators of important cellular activities, | *RAC1* | rs10951983, rs12536544, rs3813517, rs4720672,  rs836548, rs836554,  rs836556. | Negative |
| Zou et al.2016[126] | 317(NSCLC+SCLC) | DDP/CBP + GEM/PTX/NVB/VP-16/CPT-11 | Others mediators or modulators of important cellular activities, | *RhoA* | rs3811699, rs9878943 | Negative |
| Cai et al.2014[127] | 663(NSCLC) | DDP/CBP + NVB/GEM/PTX/VP-16/BEV | Others Key regulators | *CDC25A* | rs3731485, rs3731513, rs1380053, rs13099560, rs3731557. | Negative |
| Cai et al.2014[127] | 663(NSCLC) | DDP/CBP + NVB/GEM/PTX/VP-16/BEV | Others Key regulators | *CDC25B* | rs3761218, rs6116042, rs1010608, rs2295348. | *CDC25B* rs3761218: grade 3-4 hematology toxicity (P = 0.008). |
| Cai et al.2014[127] | 663(NSCLC) | DDP/CBP + NVB/GEM/PTX/VP-16/BEV | Others Key regulators | *CDC25C* | rs3734166, rs4522986, rs6861656, rs11567952, rs11567949. | Negative |
| Jia et al.2016[43] | in a discovery group of 345 patients, in a replication group of 344 patients (NSCLC) | DDP/CBP + PEM/TXT/PTX/GEM | Others JNK and P38α | *GADD45A* | rs581000 | Negative |
| Jia et al.2016[43] | in a discovery group of 345 patients, in a replication group of 344 patients (NSCLC) | DDP/CBP + PEM/TXT/PTX/GEM | Others JNK and P38α | *GADD45B* | rs2024144 | *GADD45B* rs2024144: grade 3-4 hematologic toxicity in discovery group (CT + TT vs. CC OR = 1.80; 95% CI: 1.01-3.18; P = 0.046). *GADD45B* rs2024144: grade 3-4 hematologic toxicity in replication group (CT + TT vs. CC OR = 1.87; 95% CI: 1.02-3.42; P = 0.042). *GADD45B* rs2024144: grade 3-4 hematologic toxicity in all patients (CT + TT vs. CC OR = 1.80; 95% CI: 1.19-2.71; P = 0.005). |
| Jia et al.2016[43] | in a discovery group of 345 patients, in a replication group of 344 patients (NSCLC) | DDP/CBP + PEM/TXT/PTX/GEM | Others JNK and P38α | *GADD45G* | rs8252 | Negative |
| Jia et al.2016[43] | in a discovery group of 345 patients, in a replication group of 344 patients (NSCLC) | DDP/CBP + PEM/TXT/PTX/GEM | Others JNK and P38α | *MAP2K4* | rs3826392 | Negative |
| Jia et al.2016[43] | in a discovery group of 345 patients, in a replication group of 344 patients (NSCLC) | DDP/CBP + PEM/TXT/PTX/GEM | Others JNK and P38α | *MAP2K7* | rs2115107 | Negative |
| Jia et al.2016[43] | in a discovery group of 345 patients, in a replication group of 344 patients (NSCLC) | DDP/CBP + PEM/TXT/PTX/GEM | Others JNK and P38α | *MAP2K7* | rs3679 | Negative |
| Jia et al.2016[43] | in a discovery group of 345 patients, in a replication group of 344 patients (NSCLC) | DDP/CBP + PEM/TXT/PTX/GEM | Others JNK and P38α | *MAP3K4* | rs1488 | Negative |
| Jia et al.2016[43] | in a discovery group of 345 patients, in a replication group of 344 patients (NSCLC) | DDP/CBP + PEM/TXT/PTX/GEM | Others JNK and P38α | *MAP3K4* | rs678290 | Negative |
| Jia et al.2016[43] | in a discovery group of 345 patients, in a replication group of 344 patients (NSCLC) | DDP/CBP + PEM/TXT/PTX/GEM | Others JNK and P38α | *MAPK14* | rs3804451 | Negative |
| Jia et al.2016[43] | in a discovery group of 345 patients, in a replication group of 344 patients (NSCLC) | DDP/CBP + PEM/TXT/PTX/GEM | Others JNK and P38α | *MAPK8* | rs10857561 | Negative |
| Jia et al.2016[43] | in a discovery group of 345 patients, in a replication group of 344 patients (NSCLC) | DDP/CBP + PEM/TXT/PTX/GEM | Others JNK and P38α | *MAPK9* | rs6703 | Negative |
| Tan et al.2014[128] | 1004(NSCLC) | DDP/CBP + NVB/GEM/PTX/TXT Other DDP/CBP combinations | Others MIF signaling pathway | *MIF, JAB1, SKP1, CUL1, RBX1, NEDD8, CAND1,* and *CD74* | 40 SNPs | *MIF* rs4822443 (G>A): increased risk of grade 3-4 hematologic toxicity (AG vs. GG OR = 1.42; 95% CI: 1.04-1.94; P < 0.05)  *MIF* rs4822446 (A>G): increased risk of grade 3-4 hematologic toxicity (AG vs. AA OR = 1.39; 95% CI: 1.02-1.90; P < 0.05)  *MIF* rs12485068 (A>G): increased risk of grade 3-4 hematologic toxicity (AG vs. AA OR = 1.42; 95% CI: 1.04-1.94; P < 0.05)  *CD74* rs2748249 (C>A): increased risk of grade 3-4 hematologic toxicity (CA vs. CC OR = 1.72; 95% CI: 1.24-2.39; P < 0.001)  *CD74* rs1560661 (G>A): decreased risk of grade 3-4 hematologic toxicity (GG vs. AA OR = 0.42; 95% CI: 0.25-0.70; P < 0.001) |

Abbreviations: ADM: doxorubicin; BER: base excision repair; BEV: bevacizumab; BLM: bleomycin; CAP: capecitabine; CBP: carboplatin; CI: confidence interval; CPT-11:irinotecan; CTCAE: Common Terminology Criteria for Adverse Events; CTX: cyclophosphamide; DDP: cisplatin; DSB: double-strand break repair; GEM: gemcitabine; L-OHP: oxaliplatin; LV: leucovorin; MMR: mismatch repair; MMC: mitomycin C; NER: nucleotide excision repair; NSCLC: non-small cell lung cancer; NVB: navelbine; OR: odds ratios; PEM: pemetrexed; PTX: paclitaxel; SCLC: small cell lung cancer; TLS: translesion DNA synthesis. TXT: docetaxel; VCR: vincristine; VDS: vindesine; VP-16: etoposide; 5-FU: fluorouracil.
