# Supplementary material for: Genetic polymorphisms and platinum-induced hematological toxicity: a systematic review
Source: Front Pharmacol. 2024 Aug 21;15:1445328. doi: 10.3389/fphar.2024.1445328 (PMC11371761; doi:10.3389/fphar.2024.1445328)
Supplement: Supplementary file 3 [file Table7.docx]

Supplementary Material

## Supplementary Table 7 Overview of genetic polymorphisms investigated more than twice for a possible association with platinum-induced hematological toxicity

| Reference | Sample size (Cancer) | Treatment | Pathway | Gene | Rs number | Association |
| --- | --- | --- | --- | --- | --- | --- |
| Sun et al.2018[57] | 1004(NSCLC) | DDP/CBP + NVB/GEM/PTX/TXT Other DDP/CBP combinations | Transporter | *SCL31A1 (CTR1)* | rs10981699 | Negative |
| Xu et al.2012[58] | 204(NSCLC) | DDP + GEM/VP-16/TXT/VDS | Transporter | *SCL31A1 (CTR1)* | rs10981699 | Negative |
| Sun et al.2018[57] | 1004(NSCLC) | DDP/CBP + NVB/GEM/PTX/TXT Other DDP/CBP combinations | Transporter | *SCL31A1 (CTR1)* | rs10817465 | Negative |
| Xu et al.2012[58] | 204(NSCLC) | DDP + GEM/VP-16/TXT/VDS | Transporter | *SCL31A1 (CTR1)* | rs10817465 | Negative |
| Kumpiro et al.2016[38] | 32(NSCLC) | CBP + GEM | Transporter | *SCL31A1 (CTR1)* | rs12686377 | Negative |
| Xu et al.2012[58] | 204(NSCLC) | DDP + GEM/VP-16/TXT/VDS | Transporter | *SCL31A1 (CTR1)* | rs12686377 | Negative |
| Lee et al.2013[37] | 292(Colon cancer) | L-OHP + LV + 5-FU | Transporter | *ABCC2(MRP2)* | rs717620 (C-24T) | *ABCC2* rs717620: lower incident rate of grade 3-4 thrombocytopenia [5.6 % (9 out of 160 patients with CC) vs 0.8 % (1 out of 124 with CT or TT), P = 0.047]. |
| Han et al.2011[62] | 445(NSCLC) | DDP/CBP + NVB/GEM/PTX/TXT Other DDP/CBP combinations | Transporter | *ABCC2(MRP2)* | rs717620 (C-24T) | Negative |
| Qian et al.2016[63] | 403(NSCLC) | DDP/CBP + GEM/PEM/PTX/TXT/NVB | Transporter | *ABCC2(MRP2)* | rs717620 (C-24T) | Negative |
| Han et al.2006[55] | 107(NSCLC) | DDP + CPT-11 | Transporter | *ABCC2(MRP2)* | rs717620 (C-24T) | Negative |
| De Troia et al.2018[65] | 82(NSCLC + SCLC) | DDP/CBP + VP-16/NVB DDP + GEM/PEM/TXT DDP monotherapy | Transporter | *ABCC2(MRP2)* | rs717620 (C-24T) | Negative |
| Marsh et al.2007[29] | 914 Discovery cohort and validation cohort in ratio 2:1(Ovarian cancer) | CBP + PTX/TXT | Transporter | *ABCC2(MRP2)* | rs717620 (C-24T) | Negative |
| Han et al.2011[62] | 445(NSCLC) | DDP/CBP + NVB/GEM/PTX/TXT Other DDP/CBP combinations | Transporter | *ABCC2(MRP2)* | rs3740066 (C3972T, Ile1324Ile) | *ABCC2(MRP2)* rs3740066: increased risk of grade 3-4 thrombocytopenia (CT + TT vs. CC OR= 2.43; 95% CI: 1.06-5.56; P = 0.034) |
| Lee et al.2013[37] | 292(Colon cancer) | L-OHP + LV + 5-FU | Transporter | *ABCC2(MRP2)* | rs3740066 (C3972T, Ile1324Ile) | Negative |
| Ruzzo et al.2014[33] | 517(Colorectal cancer) | L-OHP + 5-FU + LV L-OHP + CAP | Transporter | *ABCC2(MRP2)* | rs3740066 (C3972T, Ile1324Ile) | Negative |
| Qian et al.2016[63] | 403(NSCLC) | DDP/CBP + GEM/PEM/PTX/TXT/NVB | Transporter | *ABCC2(MRP2)* | rs3740066 (C3972T, Ile1324Ile) | Negative |
| Han et al.2006[55] | 107(NSCLC) | DDP + CPT-11 | Transporter | *ABCC2(MRP2)* | rs3740066 (C3972T, Ile1324Ile) | Negative |
| Han et al.2011[62] | 445(NSCLC) | DDP/CBP + NVB/GEM/PTX/TXT Other DDP/CBP combinations | Transporter | *ABCC2(MRP2)* | rs2273697 (G1249A, Val417Ile) | Negative |
| Qian et al.2016[63] | 403(NSCLC) | DDP/CBP + GEM/PEM/PTX/TXT/NVB | Transporter | *ABCC2(MRP2)* | rs2273697 (G1249A, Val417Ile) | Negative |
| Han et al.2006[55] | 107(NSCLC) | DDP + CPT-11 | Transporter | *ABCC2(MRP2)* | rs2273697 (G1249A, Val417Ile) | Negative |
| Marsh et al.2007[29] | 914 Discovery cohort and validation cohort in ratio 2:1(Ovarian cancer) | CBP + PTX/TXT | Transporter | *ABCC2(MRP2)* | rs2273697 (G1249A, Val417Ile) | Negative |
| Nomura et al.2020[32] | 158(Esophageal cancer) | DDP + TXT + 5-FU | Transporter | *ABCC2(MRP2)* | rs12762549 (*+9383C>G) | *ABCC2* rs12762549: increased risk of grade 3-4 neutropenia (GG vs. GC + CC OR = 2.342; 95% CI 1.108-4.948, P = 0.026). |
| Lambrechts et al.2015[27] | 290(Ovarian cancer) | CBP + PTX CBP mono-therapy | Transporter | *ABCC2(MRP2)* | rs12762549 (*+9383C>G) | *ABCC2* rs12762549: decreased risk of grade 3-4 anemia in additive model (OR = 0.51; 95% CI: 0.33-0.81; P = 0.004). |
| Lambrechts et al.2015[27] | 290(Ovarian cancer) | CBP + PTX CBP mono-therapy | Transporter | *ABCC2(MRP2)* | rs2073337 (c.1668+148A>G) | Negative |
| Marsh et al.2007[29] | 914 Discovery cohort and validation cohort in ratio 2:1(Ovarian cancer) | CBP + PTX/TXT | Transporter | *ABCC2(MRP2)* | rs2073337 (c.1668+148A>G) | Negative |
| Nomura et al.2020[32] | 158(Esophageal cancer) | DDP + TXT + 5-FU | Transporter | *ABCB1(MDR1)* | rs1045642 (C3435T, Ile1145Ile) | *ABCB1* rs1045642: increased risk of grade 3-4 neutropenia (CT + TT vs. CC OR = 2.191; 95% CI 1.087-4.417, P = 0.028). |
| De Troia et al.2018[65] | 82(NSCLC + SCLC) | DDP/CBP + VP-16/NVB DDP + GEM/PEM/TXT DDP monotherapy | Transporter | *ABCB1(MDR1)* | rs1045642 (C3435T, Ile1145Ile) | *ABCB1* rs1045642: decreased risk of grade 3-4 hematological toxicity (CT vs. CC OR = 0.18; 95% CI: 0.05-0.65; P = 0.01, CT + TT vs. CC OR = 0.24; 95% CI: 0.07-0.75; P = 0.01). |
| Cortejoso et al.2013[51] | 106(Colorectal cancer) | L-OHP + 5-FU + LV L-OHP + CAP | Transporter | *ABCB1(MDR1)* | rs1045642 (C3435T, Ile1145Ile) | Negative |
| Chen et al.2010[53] | 95(NSCLC) | DDP + GEM/NVB/TXT | Transporter | *ABCB1(MDR1)* | rs1045642 (C3435T, Ile1145Ile) | Negative |
| Han et al.2006[55] | 107(NSCLC) | DDP + CPT-11 | Transporter | *ABCB1(MDR1)* | rs1045642 (C3435T, Ile1145Ile) | Negative |
| Isla et al.2004[54] | 62(NSCLC) | DDP + TXT | Transporter | *ABCB1(MDR1)* | rs1045642 (C3435T, Ile1145Ile) | Negative |
| Lambrechts et al.2015[27] | 290(Ovarian cancer) | CBP + PTX CBP mono-therapy | Transporter | *ABCB1(MDR1)* | rs1045642 (C3435T, Ile1145Ile) | Negative |
| Marsh et al.2007[29] | 914 Discovery cohort and validation cohort in ratio 2:1(Ovarian cancer) | CBP + PTX/TXT | Transporter | *ABCB1(MDR1)* | rs1045642 (C3435T, Ile1145Ile) | Negative |
| Ferracini et al.2020[41] | 112(Ovarian cancer (Epithelial ovarian cancer)) | CBP + PTX CBP mono-therapy | Transporter | *ABCB1(MDR1)* | rs1045642 (C3435T, Ile1145Ile) | Negative |
| Kim et al.2009[36] | 118(Ovarian cancer (Epithelial ovarian cancer)) | DDP/CBP + PTX CBP + TXT | Transporter | *ABCB1(MDR1)* | rs1045642 (C3435T, Ile1145Ile) | Negative |
| Lavanderos et al.2019[80] | 119(Testicular Cancer) | DDP + BLM + VP-16 | Transporter | *ABCB1(MDR1)* | rs1045642 (C3435T, Ile1145Ile) | Negative |
| Qian et al.2016[63] | 403(NSCLC) | DDP/CBP + GEM/PEM/PTX/TXT/NVB | Transporter | *ABCB1(MDR1)* | rs1045642 (C3435T, Ile1145Ile) | Negative |
| Han et al.2006[55] | 107(NSCLC) | DDP + CPT-11 | Transporter | *ABCB1(MDR1)* | rs2032582 (G2677T/A, Ala893Ser) | *ABCB1* rs2032582: associated with grade 4 neutropenia (the incident rate of grade 4 neutropenia for GG, GT/GA and TT/TA/AA were 34.6%, 53.8%, and 11.5%, P = 0.030). |
| Cortejoso et al.2013[51] | 106(Colorectal cancer) | L-OHP + 5-FU + LV L-OHP + CAP | Transporter | *ABCB1(MDR1)* | rs2032582 (G2677T/A, Ala893Ser) | Negative |
| Nomura et al.2020[32] | 158(Esophageal cancer) | DDP + TXT + 5-FU | Transporter | *ABCB1(MDR1)* | rs2032582 (G2677T/A, Ala893Ser) | Negative |
| Chen et al.2010[53] | 95(NSCLC) | DDP + GEM/NVB/TXT | Transporter | *ABCB1(MDR1)* | rs2032582 (G2677T/A, Ala893Ser) | Negative |
| Marsh et al.2007[29] | 914 Discovery cohort and validation cohort in ratio 2:1(Ovarian cancer) | CBP + PTX/TXT | Transporter | *ABCB1(MDR1)* | rs2032582 (G2677T/A, Ala893Ser) | Negative |
| Ferracini et al.2020[41] | 112(Ovarian cancer (Epithelial ovarian cancer)) | CBP + PTX CBP mono-therapy | Transporter | *ABCB1(MDR1)* | rs2032582 (G2677T/A, Ala893Ser) | Negative |
| Kim et al.2009[36] | 118(Ovarian cancer (Epithelial ovarian cancer)) | CBP + PTX/TXT DDP + PTX | Transporter | *ABCB1(MDR1)* | rs2032582 (G2677T/A, Ala893Ser) | Negative |
| Lambrechts et al.2015[27] | 290(Ovarian cancer) | CBP + PTX CBP mono-therapy | Transporter | *ABCB1(MDR1)* | rs1128503 (C1236T, Gly412Gly) | *ABCB1* rs1128503: increased risk of grade 3-4 anemia in additive model (OR = 1.71; 95% CI: 1.07-2.71; P = 0.023). |
| Ferracini et al.2020[41] | 112(Ovarian cancer (Epithelial ovarian cancer)) | CBP + PTX CBP mono-therapy | Transporter | *ABCB1(MDR1)* | rs1128503 (C1236T, Gly412Gly) | *ABCB1* rs1128503: increased risk of grade 1-4 thrombocytopenia (TT vs. CC OR = 3.63; 95% CI: 0.98-13.47; P = 0.05, TT vs. CT + CC OR = 3.50; 95% CI: 1.12-10.97; P = 0.03). |
| Cortejoso et al.2013[51] | 106(Colorectal cancer) | L-OHP + 5-FU + LV L-OHP + CAP | Transporter | *ABCB1(MDR1)* | rs1128503 (C1236T, Gly412Gly) | Negative |
| Nomura et al.2020[32] | 158(Esophageal cancer) | DDP + TXT + 5-FU | Transporter | *ABCB1(MDR1)* | rs1128503 (C1236T, Gly412Gly) | Negative |
| Marsh et al.2007[29] | 914 Discovery cohort and validation cohort in ratio 2:1(Ovarian cancer) | CBP + PTX/TXT | Transporter | *ABCB1(MDR1)* | rs1128503 (C1236T, Gly412Gly) | Negative |
| Han et al.2006[55] | 107(NSCLC) | DDP + CPT-11 | Transporter | *ABCB1(MDR1)* | rs1128503 (C1236T, Gly412Gly) | Negative |
| Wang et al.2021[69] | 1004(NSCLC) | DDP/CBP + NVB/GEM/PTX/TXT Other DDP/CBP combinations | Transporter | *ABCG2* | rs2231142 (421C>A, Gln141Lys) | Negative |
| Nomura et al.2020[32] | 158(Esophageal cancer) | DDP + TXT + 5-FU | Transporter | *ABCG2* | rs2231142 (421C>A, Gln141Lys) | Negative |
| Lambrechts et al.2015[27] | 290(Ovarian cancer) | CBP + PTX CBP mono-therapy | Transporter | *ABCG2* | rs2231142 (421C>A, Gln141Lys) | Negative |
| Han et al.2006[55] | 107(NSCLC) | DDP + CPT-11 | Transporter | *ABCG2* | rs2231142 (421C>A, Gln141Lys) | Negative |
| Marsh et al.2007[29] | 914  Discovery cohort  and validation  cohort in ratio 2:1(Ovarian cancer) | CBP + PTX/TXT | Transporter | *ABCG2* | rs2231142 (421C>A, Gln141Lys) | Negative |
| Wang et al.2021[69] | 1004(NSCLC) | DDP/CBP + NVB/GEM/PTX/TXT Other DDP/CBP combinations | Transporter | *ABCG2* | rs2231137 (34G>A, Val12Met) | Negative |
| Nomura et al.2020[32] | 158(Esophageal cancer) | DDP + TXT + 5-FU | Transporter | *ABCG2* | rs2231137 (34G>A, Val12Met) | Negative |
| Han et al.2006[55] | 107(NSCLC) | DDP + CPT-11 | Transporter | *ABCG2* | rs2231137 (34G>A, Val12Met) | Negative |
| Qian et al.2016[63] | 403(NSCLC) | DDP/CBP + GEM/PEM/PTX/TXT/NVB | Transporter | *OCT2* | rs316019 (808G/T, p.270Ala > Ser) | *OCT2* rs316019: decreased risk of grade 3-4 hematological toxicity in additive model (OR = 0.58; 95% CI: 0.34-0.97; P = 0.039). |
| Iwata et al.2012[31] | 53(advanced carcinomas) | DDP + 5-FU/GEM/TXT/VP-16/PEM/CPT-11 | Transporter | *OCT2* | rs316019 (808G/T, p.270Ala > Ser) | Negative |
| Qian et al.2016[63] | 403(NSCLC) | DDP/CBP + GEM/PEM/PTX/TXT/NVB | Transporter | *MATE1* | rs2289669 (G/A) | *MATE1* rs2289669: increased risk of grade 3-4 hematological toxicity in recessive model (OR = 1.92; 95% CI: 1.13-3.25; P = 0.016). |
| Iwata et al.2012[31] | 53(advanced carcinomas) | DDP + 5-FU/GEM/TXT/VP-16/PEM/CPT-11 | Transporter | *MATE1* | rs2289669 (G/A) | Negative |
| Ferracini et al.2020[41] | 112(Ovarian cancer (Epithelial ovarian cancer)) | CBP + PTX CBP mono-therapy | Metabolism | *GSTP1* | rs1695 (A313G, Ile105Val) | *GSTP1* rs1695: grade 3-4 anemia (AG vs. AA OR = 0.16; 95% CI: 0.03-0.84; P = 0.03, AG + GG vs. AA OR= 0.17; 95% CI: 0.04-0.69; P = 0.01), grade 3-4 thrombocytopenia (AG vs. AA OR = 0.32; 95% CI: 0.12-0.82; P = 0.01, GG vs. AA OR = 0.11; 95% CI: 0.02-0.59; P < 0.01, AG + GG vs. AA OR = 0.27; 95% CI: 0.12-0.64; P < 0.01, GG vs. AA + AG OR = 0.18; 95% CI: 0.03-0.85; P = 0.03). |
| Kim et al.2009[36] | 118(Ovarian cancer (Epithelial ovarian cancer)) | DDP/CBP + PTX CBP + TXT | Metabolism | *GSTP1* | rs1695 (A313G, Ile105Val) | *GSTP1* rs1695: associated with grade 3-4 hematological toxicity (the incident rate of grade 3-4 hematology toxicity for AG/GG and AA were 54.2% and 78.7%, P = 0.015). |
| Bushra et al.2020[74] | 285(NSCLC) | DDP/CBP + GEM/NVB/PTX/TXT | Metabolism | *GSTP1* | rs1695 (A313G, Ile105Val) | *GSTP1* rs1695: decreased risk of grade 3-4 anemia (GG vs. AA OR = 0.29; 95% CI: 0.10-0.87; P = 0.027) and grade 3-4 neutropenia (GG vs. AA OR = 0.31; 95% CI: 0.10-0.96; P = 0.043). |
| Yoshihama et al.2018[25] | 320(Ovarian fallopian tube, peritoneal, uterine, or cervical cancer) | CBP + PTX | Metabolism | *GSTP1* | rs1695 (A313G, Ile105Val) | *GSTP1* rs1695: decreased risk of severe hematotoxicity (including neutropenia G4, thrombocytopenia ≥ G3 and anemia ≥ G3)(A allele vs. G allele OR = 5.71; 95% CI: 1.77-18.44; P = 0.00034) |
| Walia et al.2021a[34] | 317(NSCLC + SCLC) | DDP/CBP + PEM/CPT-11/TXT/PTX/GEM | Metabolism | *GSTP1* | rs1695 (A313G, Ile105Val) | *GSTP1* rs1695: increased risk of grade 3-4 anemia (AG vs. AA OR = 2.12; 95% CI: 0.97-4.62; P = 0.04) and grade 2-4 leukopenia (GG vs. AA OR = 2.41; 95% CI: 1.39-4.18; P = 0.001). |
| Liblab et al.2019[47] | 52(Ovarian cancer (Epithelial ovarian cancer)) | CBP + PTX CBP mono-therapy | Metabolism | *GSTP1* | rs1695 (A313G, Ile105Val) | *GSTP1* rs1695: higher incident rate of grade 2-4 anemia [46.34%(AA) vs. 81.82%(AG), P = 0.036]. |
| Cortejoso et al.2013[51] | 106(Colorectal cancer) | L-OHP + 5-FU + LV L-OHP + CAP | Metabolism | *GSTP1* | rs1695 (A313G, Ile105Val) | Negative |
| Nomura et al.2020[32] | 158(Esophageal cancer) | DDP + TXT + 5-FU | Metabolism | *GSTP1* | rs1695 (A313G, Ile105Val) | Negative |
| Seo et al.2009[52] | 75(Gastric cancer) | L-OHP + 5-FU + LV | Metabolism | *GSTP1* | rs1695 (A313G, Ile105Val) | Negative |
| Erčulj et al.2012[48] | 94(Malignant mesothelioma) | DDP/CBP + GEM/PEM DDP + MMC + VCR | Metabolism | *GSTP1* | rs1695 (A313G, Ile105Val) | Negative |
| De Troia et al.2018[65] | 82(NSCLC + SCLC) | DDP/CBP + VP-16/NVB DDP + GEM/PEM/TXT DDP monotherapy | Metabolism | *GSTP1* | rs1695 (A313G, Ile105Val) | Negative |
| Lambrechts et al.2015[27] | 290(Ovarian cancer) | CBP + PTX CBP mono-therapy | Metabolism | *GSTP1* | rs1695 (A313G, Ile105Val) | Negative |
| Khrunin et al.2012[40] | 104(Ovarian cancer) | DDP + CTX | Metabolism | *GSTP1* | rs1695 (A313G, Ile105Val) | Negative |
| Marsh et al.2007[29] | 914 Discovery cohort and validation cohort in ratio 2:1(Ovarian cancer) | CBP + PTX/TXT | Metabolism | *GSTP1* | rs1695 (A313G, Ile105Val) | Negative |
| Lavanderos et al.2019[80] | 119(Testicular Cancer) | DDP + BLM + VP-16 | Metabolism | *GSTP1* | rs1695 (A313G, Ile105Val) | Negative |
| Lee et al.2013[37] | 292(Colon cancer) | L-OHP + LV + 5-FU | Metabolism | *GSTP1* | rs1695 (A313G, Ile105Val) | Negative |
| Ruzzo et al.2014[33] | 517(Colorectal cancer) | L-OHP + 5-FU + LV L-OHP + CAP | Metabolism | *GSTP1* | rs1695 (A313G, Ile105Val) | Negative |
| Deng et al.2015[89] | 97(NSCLC) | DDP + GEM/NVB/PTX/TXT | Metabolism | *GSTP1* | rs1695 (A313G, Ile105Val) | Negative |
| Erčulj et al.2012[48] | 94(Malignant mesothelioma) | DDP/CBP + GEM/PEM DDP + MMC + VCR | Metabolism | *GSTP1* | rs1138272 (c.341 C>T, Ala114Val) | Negative |
| Lambrechts et al.2015[27] | 290(Ovarian cancer) | CBP + PTX CBP mono-therapy | Metabolism | *GSTP1* | rs1138272 (c.341 C>T, Ala114Val) | Negative |
| Khrunin et al.2012[40] | 104(Ovarian cancer) | DDP + CTX | Metabolism | *GSTP1* | rs1138272 (c.341 C>T, Ala114Val) | Negative |
| Marsh et al.2007[29] | 914 Discovery cohort and validation cohort in ratio 2:1(Ovarian cancer) | CBP + PTX/TXT | Metabolism | *GSTP1* | rs1138272 (c.341 C>T, Ala114Val) | Negative |
| Lee et al.2013[37] | 292(Colon cancer) | L-OHP + LV + 5-FU | Metabolism | *GSTT1* | Gene deletion | Negative |
| Ruzzo et al.2014[33] | 517(Colorectal cancer) | L-OHP + 5-FU + LV L-OHP + CAP | Metabolism | *GSTT1* | Gene deletion | Negative |
| Cortejoso et al.2013[51] | 106(Colorectal cancer) | L-OHP + 5-FU + LV L-OHP + CAP | Metabolism | *GSTT1* | Gene deletion | Negative |
| Nomura et al.2020[32] | 158(Esophageal cancer) | DDP + TXT + 5-FU | Metabolism | *GSTT1* | Gene deletion | Negative |
| Seo et al.2009[52] | 75(Gastric cancer) | L-OHP + 5-FU + LV | Metabolism | *GSTT1* | Gene deletion | Negative |
| Erčulj et al.2012[48] | 94(Malignant mesothelioma) | DDP/CBP + GEM/PEM DDP + MMC + VCR | Metabolism | *GSTT1* | Gene deletion | Negative |
| Khrunin et al.2012[40] | 104(Ovarian cancer) | DDP + CTX | Metabolism | *GSTT1* | Gene deletion | Negative |
| Kim et al.2009[36] | 118(Ovarian cancer (Epithelial ovarian cancer)) | DDP/CBP + PTX CBP + TXT | Metabolism | *GSTT1* | Gene deletion | Negative |
| Lavanderos et al.2019[80] | 119(Testicular Cancer) | DDP + BLM + VP-16 | Metabolism | *GSTT1* | Gene deletion | Negative |
| Erčulj et al.2012[48] | 94(Malignant mesothelioma) | DDP/CBP + GEM/PEM DDP + MMC + VCR | Metabolism | *GSTM1* | Gene deletion | *GSTM1* gene deletion: decreased risk of grade 2-4 leukopenia (0/0 vs. 1/1 + 1/0 OR = 0.43; 95% CI: 0.18-0.99; P = 0.048). |
| Khrunin et al.2012[40] | 104(Ovarian cancer) | DDP + CTX | Metabolism | *GSTM1* | Gene deletion | *GSTM1* gene deletion: decreased risk of grade 1-4 thrombocytopenia (0/0 vs. 1/0 OR = 0.13; 95% CI: 0.03-0.62; P = 0.005), grade 2-4 anemia (0/0 vs. 1/0 OR = 0.29; 95% CI: 0.13-0.66; P = 0.003). |
| Lee et al.2013[37] | 292(Colon cancer) | L-OHP + LV + 5-FU | Metabolism | *GSTM1* | Gene deletion | Negative |
| Ruzzo et al.2014[33] | 517(Colorectal cancer) | L-OHP + 5-FU + LV L-OHP + CAP | Metabolism | *GSTM1* | Gene deletion | Negative |
| Nomura et al.2020[32] | 158(Esophageal cancer) | DDP + TXT + 5-FU | Metabolism | *GSTM1* | Gene deletion | Negative |
| Seo et al.2009[52] | 75(Gastric cancer) | L-OHP + 5-FU + LV | Metabolism | *GSTM1* | Gene deletion | Negative |
| Kim et al.2009[36] | 118(Ovarian cancer (Epithelial ovarian cancer)) | DDP/CBP + PTX CBP + TXT | Metabolism | *GSTM1* | Gene deletion | Negative |
| Lavanderos et al.2019[80] | 119(Testicular Cancer) | DDP + BLM + VP-16 | Metabolism | *GSTM1* | Gene deletion | Negative |
| Lee et al.2013[37] | 292(Colon cancer) | L-OHP + LV + 5-FU | NER | *ERCC1* | rs11615 (C118T, Asn118Asn) | *ERCC1* rs11615: increased risk of grade 3-4 neutropenia (TT vs. TC + CC OR = 4.58, 95 % CI: 1.20-17.40, P = 0.026). |
| Lavanderos et al.2019[80] | 119(Testicular Cancer) | DDP + BLM + VP-16 | NER | *ERCC1* | rs11615 (C118T, Asn118Asn) | *ERCC1* rs11615: increased risk of grade 3-4 febrile neutropenia (TT vs. CC + CT OR = 4.89; 95% CI: 1.06-22.56; P = 0.042). |
| Lambrechts et al.2015[27] | 290(Ovarian cancer) | CBP + PTX CBP mono-therapy | NER | *ERCC1* | rs11615 (C118T, Asn118Asn) | *ERCC1* rs11615: increased risk of grade 3-4 anemia in additive model (OR = 1.61; 95% CI: 1.04-2.50; P = 0.031). |
| Zheng et al.2017[46] | 437 in the in a discovery cohort and 781 in the validation cohort(NSCLC ) | DDP/CBP + NVB/GEM/PTX/TXT/PEM | NER | *ERCC1* | rs11615 (C118T, Asn118Asn) | *ERCC1* rs11615: increased risk of grade 3-4 anemia in dominant model in discovery cohort (OR = 2.230; 95% CI: 1.041-4.775; P = 0.039). |
| Cortejoso et al.2013[51] | 106(Colorectal cancer) | L-OHP + 5-FU + LV L-OHP + CAP | NER | *ERCC1* | rs11615 (C118T, Asn118Asn) | *ERCC1* rs11615: decreased risk of grade 3-4 neutropenia (CT + TT vs. CC OR = 0.205; 95% CI: 0.061-0.690; P = 0.010) |
| Ruzzo et al.2014[33] | 517(Colorectal cancer) | L-OHP + 5-FU + LV L-OHP + CAP | NER | *ERCC1* | rs11615 (C118T, Asn118Asn) | Negative |
| Kim et al.2009[36] | 118(Epithelial ovarian cancer) | DDP/CBP + PTX CBP + TXT | NER | *ERCC1* | rs11615 (C118T, Asn118Asn) | Negative |
| Seo et al.2009[52] | 75(Gastric cancer) | L-OHP + 5-FU + LV | NER | *ERCC1* | rs11615 (C118T, Asn118Asn) | Negative |
| Erčulj et al.2012[48] | 94(Malignant mesothelioma) | DDP/CBP + GEM/PEM DDP + MMC + VCR | NER | *ERCC1* | rs11615 (C118T, Asn118Asn) | Negative |
| Bushra et al.2020[74] | 285(NSCLC) | DDP/CBP + GEM/NVB/PTX/TXT | NER | *ERCC1* | rs11615 (C118T, Asn118Asn) | Negative |
| Chen et al.2010[53] | 95(NSCLC) | DDP + GEM/NVB/TXT | NER | *ERCC1* | rs11615 (C118T, Asn118Asn) | Negative |
| Kalikaki et al.2015[146] | 107(NSCLC) | DDP/CBP + PTX/GEM  DDP + TXT/NVB | NER | *ERCC1* | rs11615 (C118T, Asn118Asn) | Negative |
| Ludovini et al.2011[28] | 189(NSCLC) | DDP + GEM/PTX/NVB | NER | *ERCC1* | rs11615 (C118T, Asn118Asn) | Negative |
| Isla et al.2004[54] | 62(NSCLC) | DDP + TXT | NER | *ERCC1* | rs11615 (C118T, Asn118Asn) | Negative |
| Tibaldi et al.2008[30] | 65(NSCLC) | DDP + GEM | NER | *ERCC1* | rs11615 (C118T, Asn118Asn) | Negative |
| Song et al.2016[81] | 1004(NSCLC+SCLC) | DDP/CBP + NVB/GEM/PTX/TXT Other DDP/CBP combinations | NER | *ERCC1* | rs11615 (C118T, Asn118Asn) | Negative |
| Khrunin et al.2012[40] | 104(Ovarian cancer) | DDP + CTX | NER | *ERCC1* | rs11615 (C118T, Asn118Asn) | Negative |
| Marsh et al.2007[29] | 914 Discovery cohort and validation cohort in ratio 2:1(Ovarian cancer) | CBP + PTX/TXT | NER | *ERCC1* | rs11615 (C118T, Asn118Asn) | Negative |
| Giovannetti et al.2011[26] | 122(Pancreatic cancer) | PEXG, PDXG, EC-GemCap | NER | *ERCC1* | rs11615 (C118T, Asn118Asn) | Negative |
| Zheng et al.2017[46] | 437 in the in a discovery cohort and 781 in the validation cohort (NSCLC) | DDP/CBP + NVB/GEM/PTX/TXT/PEM | NER | *ERCC1* | rs3212986 (C8092A) | *ERCC1* rs3212986: decreased risk of grade 3-4 hematologic toxicity in recessive model in discovery cohort (OR = 0.326; 95% CI: 0.123-0.861; P = 0.024). |
| Erčulj et al.2012[48] | 94(Malignant mesothelioma) | DDP/CBP + GEM/PEM DDP + MMC + VCR | NER | *ERCC1* | rs3212986 (C8092A) | *ERCC1* rs3212986: decreased risk of grade 2-4 leukopenia (CA + AA vs. CC OR = 0.18; 95% CI: 0.04-0.86; P = 0.032). |
| Lee et al.2013[37] | 292(Colon cancer) | L-OHP + LV + 5-FU | NER | *ERCC1* | rs3212986 (C8092A) | Negative |
| Seo et al.2009[52] | 75(Gastric cancer) | L-OHP + 5-FU + LV | NER | *ERCC1* | rs3212986 (C8092A) | Negative |
| KimCurran et al.2011[145] | 300(NSCLC) | DDP/CBP + GEM/NVB/PTX | NER | *ERCC1* | rs3212986 (C8092A) | Negative |
| Kalikaki et al.2015[146] | 107(NSCLC) | DDP/CBP + PTX/GEM  DDP + TXT/NVB | NER | *ERCC1* | rs3212986 (C8092A) | Negative |
| Song et al.2016[81] | 1004(NSCLC+SCLC) | DDP/CBP + NVB/GEM/PTX/TXT Other DDP/CBP combinations | NER | *ERCC1* | rs3212986 (C8092A) | Negative |
| Khrunin et al.2012[40] | 104(Ovarian cancer) | DDP + CTX | NER | *ERCC1* | rs3212986 (C8092A) | Negative |
| Marsh et al.2007[29] | 914 Discovery cohort and validation cohort in ratio 2:1(Ovarian cancer) | CBP + PTX/TXT | NER | *ERCC1* | rs3212986 (C8092A) | Negative |
| Kim et al.2009[36] | 118(Ovarian cancer (Epithelial ovarian cancer)) | DDP/CBP + PTX CBP + TXT | NER | *ERCC1* | rs3212986 (C8092A) | Negative |
| Liblab et al.2019[47] | 52(Ovarian cancer (Epithelial ovarian cancer)) | CBP + PTX CBP mono-therapy | NER | *ERCC1* | rs3212986 (C8092A) | Negative |
| Lavanderos et al.2019[80] | 119(Testicular Cancer) | DDP + BLM + VP-16 | NER | *ERCC1* | rs3212986 (C8092A) | Negative |
| Song et al.2016[81] | 1004(NSCLC+SCLC) | DDP/CBP + NVB/GEM/PTX/TXT Other DDP/CBP combinations | NER | *ERCC1* | rs3212961(17677G>T) | Negative |
| Lambrechts et al.2015[27] | 290(Ovarian cancer) | CBP + PTX CBP mono-therapy | NER | *ERCC1* | rs3212961(17677G>T) | Negative |
| Marsh et al.2007[29] | 914 Discovery cohort and validation cohort in ratio 2:1(Ovarian cancer) | CBP + PTX/TXT | NER | *ERCC1* | rs3212961(17677G>T) | Negative |
| Isla et al.2004[54] | 62(NSCLC) | DDP + TXT | NER | *ERCC2/XPD* | rs13181(A>C, Lys751Gln) | *ERCC2/XPD* rs13181: associated with grade 2-4 neutropenia (the incident rate of grade 2-4 neutropenia for Lys/Lys, Lys/Gln and Gln/Gln were 48%, 19%, and 14%, P = 0.04). |
| Cortejoso et al.2013[51] | 106(Colorectal cancer) | L-OHP + 5-FU + LV L-OHP + CAP | NER | *ERCC2/XPD* | rs13181(A>C, Lys751Gln) | Negative |
| Lee et al.2013[37] | 292(Colon cancer) | L-OHP + LV + 5-FU | NER | *ERCC2/XPD* | rs13181(A>C, Lys751Gln) | Negative |
| Ruzzo et al.2014[33] | 517(Colorectal cancer) | L-OHP + 5-FU + LV L-OHP + CAP | NER | *ERCC2/XPD* | rs13181(A>C, Lys751Gln) | Negative |
| Nairuz et al.2021[39] | 180(Lung cancer) | DDP/CBP + VP-16/PTX/TXT CBP + GEM/ADM | NER | *ERCC2/XPD* | rs13181(A>C, Lys751Gln) | Negative |
| Corrigan et al.2014[42] | 136(NSCLC + Malignant mesothelioma) | DDP + PEM CBP + PEM | NER | *ERCC2/XPD* | rs13181(A>C, Lys751Gln) | Negative |
| Erčulj et al.2012[48] | 94(Malignant mesothelioma) | DDP/CBP + GEM/PEM DDP + MMC + VCR | NER | *ERCC2/XPD* | rs13181(A>C, Lys751Gln) | Negative |
| Kalikaki et al.2015[146] | 107(NSCLC) | DDP/CBP + PTX/GEM  DDP + TXT/NVB | NER | *ERCC2/XPD* | rs13181(A>C, Lys751Gln) | Negative |
| Ludovini et al.2011[28] | 189(NSCLC) | DDP + GEM/PTX/NVB | NER | *ERCC2/XPD* | rs13181(A>C, Lys751Gln) | Negative |
| Tibaldi et al.2008[30] | 65(NSCLC) | DDP + GEM | NER | *ERCC2/XPD* | rs13181(A>C, Lys751Gln) | Negative |
| Zheng et al.2017[46] | 437 in the in a discovery cohort and 781 in the validation cohort (NSCLC) | DDP/CBP + NVB/GEM/PTX/TXT/PEM | NER | *ERCC2/XPD* | rs13181(A>C, Lys751Gln) | Negative |
| Song et al.2016[81] | 1004(NSCLC+SCLC) | DDP/CBP + NVB/GEM/PTX/TXT Other DDP/CBP combinations | NER | *ERCC2/XPD* | rs13181(A>C, Lys751Gln) | Negative |
| Khrunin et al.2012[40] | 104(Ovarian cancer) | DDP + CTX | NER | *ERCC2/XPD* | rs13181(A>C, Lys751Gln) | Negative |
| Marsh et al.2007[29] | 914 Discovery cohort and validation cohort in ratio 2:1(Ovarian cancer) | CBP + PTX/TXT | NER | *ERCC2/XPD* | rs13181(A>C, Lys751Gln) | Negative |
| Kim et al.2009[36] | 118(Ovarian cancer (Epithelial ovarian cancer)) | DDP/CBP + PTX CBP + TXT | NER | *ERCC2/XPD* | rs13181(A>C, Lys751Gln) | Negative |
| Giovannetti et al.2011[26] | 122(Pancreatic cancer) | PEXG, PDXG, EC-GemCap | NER | *ERCC2/XPD* | rs13181(A>C, Lys751Gln) | Negative |
| Lavanderos et al.2019[80] | 119(Testicular Cancer) | DDP + BLM + VP-16 | NER | *ERCC2/XPD* | rs13181(A>C, Lys751Gln) | Negative |
| Erčulj et al.2012[48] | 94(Malignant mesothelioma) | DDP/CBP + GEM/PEM DDP + MMC + VCR | NER | *ERCC2/XPD* | rs1799793 (G23591A, Asp312Asn) | *ERCC2/XPD* rs1799793: decreased risk of grade 1-4 thrombocytopenia (Asp/Asn + Asn/Asn vs. Asp/Asp OR = 0.15; 95% CI: 0.04-0.61; P = 0.008). |
| Khrunin et al.2012[40] | 104(Ovarian cancer) | DDP + CTX | NER | *ERCC2/XPD* | rs1799793 (G23591A, Asp312Asn) | *ERCC2/XPD* rs1799793: associated with grade 1-4 thrombocytopenia (Asp/Asn vs. Asp/Asp + Asn/Asn OR = 4.05; 95% CI: 1.21-13.58; P = 0.027), grade 2-4 anemia (Asp/Asn vs. Asp/Asp + Asn/Asn OR = 2.32; 95% CI: 1.05-5.13; P = 0.048). |
| Lee et al.2013[37] | 292(Colon cancer) | L-OHP + LV + 5-FU | NER | *ERCC2/XPD* | rs1799793 (G23591A, Asp312Asn) | Negative |
| Ruzzo et al.2014[33] | 517(Colorectal cancer) | L-OHP + 5-FU + LV L-OHP + CAP | NER | *ERCC2/XPD* | rs1799793 (G23591A, Asp312Asn) | Negative |
| Wu et al.2009[83] | 209(NSCLC) | DDP/CBP + NVB/GEM/PTX/TXT Other DDP/CBP combinations | NER | *ERCC2/XPD* | rs1799793 (G23591A, Asp312Asn) | Negative |
| Kalikaki et al.2015[146] | 107(NSCLC) | DDP/CBP + PTX/GEM  DDP + TXT/NVB | NER | *ERCC2/XPD* | rs1799793 (G23591A, Asp312Asn) | Negative |
| Isla et al.2004[54] | 62(NSCLC) | DDP + TXT | NER | *ERCC2/XPD* | rs1799793 (G23591A, Asp312Asn) | Negative |
| Tibaldi et al.2008[30] | 65(NSCLC) | DDP + GEM | NER | *ERCC2/XPD* | rs1799793 (G23591A, Asp312Asn) | Negative |
| Zheng et al.2017[46] | 437 in the in a discovery cohort and 781 in the validation cohort (NSCLC) | DDP/CBP + NVB/GEM/PTX/TXT/PEM | NER | *ERCC2/XPD* | rs1799793 (G23591A, Asp312Asn) | Negative |
| Song et al.2016[81] | 1004(NSCLC+SCLC) | DDP/CBP + NVB/GEM/PTX/TXT Other DDP/CBP combinations | NER | *ERCC2/XPD* | rs1799793 (G23591A, Asp312Asn) | No significant association in the whole subgroups. |
| Lambrechts et al.2015[27] | 290(Ovarian cancer) | CBP + PTX CBP mono-therapy | NER | *ERCC2/XPD* | rs1799793 (G23591A, Asp312Asn) | Negative |
| Giovannetti et al.2011[26] | 122(Pancreatic cancer) | PEXG, PDXG, EC-GemCap | NER | *ERCC2/XPD* | rs1799793 (G23591A, Asp312Asn) | Negative |
| Lavanderos et al.2019[80] | 119(Testicular Cancer) | DDP + BLM + VP-16 | NER | *ERCC2/XPD* | rs1799793 (G23591A, Asp312Asn) | Negative |
| Wu et al.2009[83] | 209(NSCLC) | DDP/CBP + NVB/GEM/PTX/TXT Other DDP/CBP combinations | NER | *ERCC2/XPD* | rs238406 (C22541A, Arg156Arg) | *ERCC2/XPD* rs238406 (Arg156Arg): increased risk of grade 3-4 hematologic toxicity (AA vs. CC OR = 3.24; 95% CI: 1.35-7.78; P = 0.009), grade 3-4 leukopenia toxicity (AA vs. CC OR = 4.88; 95% CI: 1.67-14.26; P = 0.005). |
| Lavanderos et al.2019[80] | 119(Testicular Cancer) | DDP + BLM + VP-16 | NER | *ERCC2/XPD* | rs238406 (C22541A, Arg156Arg) | *ERCC2/XPD* rs238406: increased risk of grade 3-4 leukopenia (CA+ AA vs. CC OR = 4.09; 95% CI: 1.04-15.99; P = 0.043). |
| Lee et al.2013[37] | 292(Colon cancer) | L-OHP + LV + 5-FU | NER | *ERCC2/XPD* | rs238406 (C22541A, Arg156Arg) | Negative |
| Zheng et al.2017[46] | 437 in the in a discovery cohort and 781 in the validation cohort (NSCLC) | DDP/CBP + NVB/GEM/PTX/TXT/PEM | NER | *ERCC2/XPD* | rs238406 (C22541A, Arg156Arg) | Negative |
| Song et al.2016[81] | 1004(NSCLC+SCLC) | DDP/CBP + NVB/GEM/PTX/TXT Other DDP/CBP combinations | NER | *ERCC2/XPD* | rs238406 (C22541A, Arg156Arg) | Negative |
| Wu et al.2009[83] | 209(NSCLC) | DDP/CBP + NVB/GEM/PTX/TXT Other DDP/CBP combinations | NER | *ERCC2/XPD* | rs1052555 (G>A, Asp711Asp) | Negative |
| Zheng et al.2017[46] | 437 in the in a discovery cohort and 781 in the validation cohort (NSCLC) | DDP/CBP + NVB/GEM/PTX/TXT/PEM | NER | *ERCC2/XPD* | rs1052555 (G>A, Asp711Asp) | Negative |
| Song et al.2016[81] | 1004(NSCLC+SCLC) | DDP/CBP + NVB/GEM/PTX/TXT Other DDP/CBP combinations | NER | *ERCC2/XPD* | rs1052555 (G>A, Asp711Asp) | Negative |
| Zheng et al.2017[46] | 437 in the in a discovery cohort and 781 in the validation cohort (NSCLC) | DDP/CBP + NVB/GEM/PTX/TXT/PEM | NER | *XPF* | rs1799801(T>C, Ser835Ser) | *XPF* rs1799801: increased risk of grade 3-4 hematologic toxicity in additive model (OR = 1.555; 95% CI: 1.041–2.323; P = 0.031) and grade 3-4 thrombocytopenia in dominant model (OR = 3.562; 95% CI: 1.513–8.390; P = 0.004) in discovery cohort. |
| Song et al.2016[81] | 1004(NSCLC+SCLC) | DDP/CBP + NVB/GEM/PTX/TXT Other DDP/CBP combinations | NER | *XPF* | rs1799801(T>C, Ser835Ser) | Negative |
| Bushra et al.2020[74] | 285(NSCLC) | DDP/CBP + GEM/NVB/PTX/TXT | NER | *XPC* | rs1799801(T>C, Ser835Ser) | *XPC* rs2228001: decreased risk of grade 3-4 anemia (CC vs. AA OR = 0.18; 95% CI: 0.04-0.82; P = 0.027) and increased risk of grade 3-4 neutropenia (AC vs. AA OR = 3.31; 95% CI: 1.74-6.31; P = 0.0003, AC + CC vs. AA OR = 2.63; 95% CI: 1.41-4.90; P = 0.002). |
| Zheng et al.2017[46] | 437 in the in a discovery cohort and 781 in the validation cohort (NSCLC) | DDP/CBP + NVB/GEM/PTX/TXT/PEM | NER | *XPC* | rs1799801(T>C, Ser835Ser) | *XPC* rs2228001: increased risk of grade 3-4 leukocytopenia in dominant model in discovery cohort (OR = 2.217; 95% CI: 1.054-4.665; P = 0.036) |
| Song et al.2016[81] | 1004(NSCLC+SCLC) | DDP/CBP + NVB/GEM/PTX/TXT Other DDP/CBP combinations | NER | *XPC* | rs1799801(T>C, Ser835Ser) | Negative |
| Zheng et al.2017[46] | 437 in the in a discovery cohort and 781 in the validation cohort (NSCLC) | DDP/CBP + NVB/GEM/PTX/TXT/PEM | NER | *XPG/ERCC5* | rs1047768 (T>C, His46His) | *XPG/ERCC5* rs1047768: increased risk of grade 3-4 leukocytopenia in additive model in discovery cohort (OR = 1.701; 95% CI: 1.021-2.835; P = 0.041) |
| Song et al.2016[81] | 1004(NSCLC+SCLC) | DDP/CBP + NVB/GEM/PTX/TXT Other DDP/CBP combinations | NER | *XPG/ERCC5* | rs1047768 (T>C, His46His) | Negative |
| Zheng et al.2017[46] | 437 in the in a discovery cohort and 781 in the validation cohort (NSCLC) | DDP/CBP + NVB/GEM/PTX/TXT/PEM | NER | *XPG/ERCC5* | rs17655 (G>C, His1104Asp) | *XPG/ERCC5* rs17655: increased risk of grade 3-4 thrombocytopenia in additive model in discovery cohort (OR = 2.165; 95% CI: 1.191-3.938; P = 0.011) |
| Song et al.2016[81] | 1004(NSCLC+SCLC) | DDP/CBP + NVB/GEM/PTX/TXT Other DDP/CBP combinations | NER | *XPG/ERCC5* | rs17655 (G>C, His1104Asp) | Negative |
| Zheng et al.2017[46] | 437 in the in a discovery cohort and 781 in the validation cohort (NSCLC) | DDP/CBP + NVB/GEM/PTX/TXT/PEM | NER | *CCNH* | rs2230641 (A>G, Val270Ala) | Negative |
| Song et al.2016[81] | 1004(NSCLC+SCLC) | DDP/CBP + NVB/GEM/PTX/TXT Other DDP/CBP combinations | NER | *CCNH* | rs2230641 (A>G, Val270Ala) | Negative |
| Zheng et al.2017[46] | 437 in the in a discovery cohort and 781 in the validation cohort (NSCLC) | DDP/CBP + NVB/GEM/PTX/TXT/PEM | NER | *XPA* | rs1800975 (T>C) | Negative |
| Song et al.2016[81] | 1004(NSCLC+SCLC) | DDP/CBP + NVB/GEM/PTX/TXT Other DDP/CBP combinations | NER | *XPA* | rs1800975 (T>C) | Negative |
| Chen et al.2015[108] | 317(NSCLC + SCLC) | DDP/CBP + GEM/PTX/NVB/VP-16/CPT-11 | NER | *RPA1* | rs12727 (G>C) | Negative |
| Song et al.2016[81] | 1004(NSCLC+SCLC) | DDP/CBP + NVB/GEM/PTX/TXT Other DDP/CBP combinations | NER | *RPA1* | rs12727 (G>C) | Negative |
| Chen et al.2015[108] | 317(NSCLC + SCLC) | DDP/CBP + GEM/PTX/NVB/VP-16/CPT-11 | NER | *RPA1* | rs17734 (C>T) | Negative |
| Song et al.2016[81] | 1004(NSCLC+SCLC) | DDP/CBP + NVB/GEM/PTX/TXT Other DDP/CBP combinations | NER | *RPA1* | rs17734 (C>T) | Negative |
| Bushra et al.2020[74] | 285(NSCLC) | DDP/CBP + GEM/NVB/PTX/TXT | BER | *XPCC1* | rs25487 (G23885A, Arg399Gln) | *XRCC1* rs25487: increased risk of grade 3-4 anemia (AA + AG vs. GG OR = 2.0; 95% CI: 1.19-3.35; P = 0.008, AG vs. GG OR = 2.27; 95% CI: 1.32-3.91; P = 0.003), grade 3-4 neutropenia (AG vs. GG OR = 2.37; 95% CI: 1.37-4.07; P = 0.002, AA + AG vs. GG OR = 1.98; 95% CI: 1.18-3.33; P = 0.010), grade 3-4 leukopenia (AG vs. GG OR = 1.79; 95% CI: 1.0-3.18; P = 0.049) and grade 3-4 thrombocytopenia (AG vs. GG OR = 2.14; 95% CI: 1.09-4.20; P = 0.027, AA + AG vs. GG OR = 2.11; 95% CI: 1.10-4.06; P = 0.025). |
| Peng et al.2014[88] | 235(NSCLC) | DDP + PTX/GEM/NVB/PEM | BER | *XPCC1* | rs25487 (G23885A, Arg399Gln) | *XPCC1* rs25487: increased risk of grade 3-4 hematologic toxicity (AG vs. GG OR = 1.929; 95% CI: 1.069-3.481 P = 0.029, AA vs. GG OR = 4.885; 95% CI: 1.147-20.197; P = 0.032, AG + AA vs. GG OR = 2.135; 95% CI: 1.207-3.777; P = 0.009). |
| Zheng et al.2017[46] | 437 in the in a discovery cohort and 781 in the validation cohort (NSCLC) | DDP/CBP + NVB/GEM/PTX/TXT/PEM | BER | *XPCC1* | rs25487 (G23885A, Arg399Gln) | *XRCC1* rs25487: increased risk of grade 3-4 leukocytopenia in recessive model (OR = 2.841; 95% CI: 1.051-7.681; P = 0.040) and grade 3-4 thrombocytopenia in additive model (OR = 2.033; 95% CI: 1.113–3.715; P = 0.021) in discovery cohort. |
| Deng et al.2015[89] | 97(NSCLC) | DDP + GEM/NVB/PTX/TXT | BER | *XPCC1* | rs25487 (G23885A, Arg399Gln) | *XRCC1* rs25487: decreased risk of grade 1-4 lymphopenia (AG + AA vs. GG OR = 0.323; 95% CI: 0.121-0.862; P = 0.024). |
| Khrunin et al.2012[40] | 104(Ovarian cancer) | DDP + CTX | BER | *XPCC1* | rs25487 (G23885A, Arg399Gln) | *XRCC1* rs25487: decreased risk of grade 3-4 neutropenia (GG vs. AG + AA OR = 3.02; 95% CI: 1.33-6.88; P = 0.009). |
| Lee et al.2013[37] | 292(Colon cancer) | L-OHP + LV + 5-FU | BER | *XPCC1* | rs25487 (G23885A, Arg399Gln) | Negative |
| Ruzzo et al.2014[33] | 517(Colorectal cancer) | L-OHP + 5-FU + LV L-OHP + CAP | BER | *XPCC1* | rs25487 (G23885A, Arg399Gln) | Negative |
| Kalikaki et al.2015[146] | 107(NSCLC) | DDP/CBP + PTX/GEM  DDP + TXT/NVB | BER | *XPCC1* | rs25487 (G23885A, Arg399Gln) | Negative |
| Wang et al.2008[90] | 139(NSCLC+SCLC) | DDP + NVB/PTX /TXT/GEM/VP-16 | BER | *XPCC1* | rs25487 (G23885A, Arg399Gln) | Negative |
| Marsh et al.2007[29] | 914 Discovery cohort and validation cohort in ratio 2:1(Ovarian cancer) | CBP + PTX/TXT | BER | *XPCC1* | rs25487 (G23885A, Arg399Gln) | Negative |
| Kim et al.2009[36] | 118(Ovarian cancer (Epithelial ovarian cancer)) | DDP/CBP + PTX CBP + TXT | BER | *XPCC1* | rs25487 (G23885A, Arg399Gln) | Negative |
| Liblab et al.2019[47] | 52(Ovarian cancer (Epithelial ovarian cancer)) | CBP + PTX CBP mono-therapy | BER | *XPCC1* | rs25487 (G23885A, Arg399Gln) | Negative |
| Giovannetti et al.2011[26] | 122(Pancreatic cancer) | PEXG, PDXG, EC-GemCap | BER | *XPCC1* | rs25487 (G23885A, Arg399Gln) | Negative |
| Cortejoso et al.2013[51] | 106(Colorectal cancer) | L-OHP + 5-FU + LV L-OHP + CAP | BER | *XRCC1* | rs25487 (G23885A, Arg399Gln) | Negative |
| Lee et al.2013[37] | 292(Colon cancer) | L-OHP + LV + 5-FU | BER | *XPCC1* | rs25489 (G23098A, Arg280His) | Negative |
| Zheng et al.2017[46] | 437 in the in a discovery cohort and 781 in the validation cohort (NSCLC) | DDP/CBP + NVB/GEM/PTX/TXT/PEM | BER | *XPCC1* | rs25489 (G23098A, Arg280His) | Negative |
| Khrunin et al.2012[40] | 104(Ovarian cancer) | DDP + CTX | BER | *XPCC1* | rs25489 (G23098A, Arg280His) | Negative |
| Lee et al.2013[37] | 292(Colon cancer) | L-OHP + LV + 5-FU | BER | *XPCC1* | rs1799782 (C21935T, Arg194Trp) | Negative |
| Khrunin et al.2012[40] | 104(Ovarian cancer) | DDP + CTX | BER | *XPCC1* | rs1799782 (C21935T, Arg194Trp) | Negative |
| Kim et al.2009[36] | 118(Ovarian cancer (Epithelial ovarian cancer)) | CBP + PTX/TXT DDP + PTX | BER | *XPCC1* | rs1799782 (C21935T, Arg194Trp) | Negative |
| Wang et al.2008[90] | 139(NSCLC+SCLC) | DDP + NVB/PTX /TXT/GEM/VP-16 | BER | *XPCC1* | rs1799782 (C21935T, Arg194Trp) | Negative |
| Zheng et al.2017[46] | 437 in the in a discovery cohort and 781 in the validation cohort (NSCLC) | DDP/CBP + NVB/GEM/PTX/TXT/PEM | BER | *APE1* | rs1130409 (T>G, Asp148Glu) | *APE1* rs1130409: decreased risk of grade 3-4 leukocytopenia in dominant model (OR = 0.460; 95% CI: 0.241-0.879; P = 0.019), grade 3-4 neutropenia in dominant model (OR = 0.557; 95% CI: 0.321-0.967; P = 0.038) in discovery cohort. |
| Peng et al.2014[88] | 235(NSCLC) | DDP + PTX/GEM/NVB/PEM | BER | *APE1* | rs1130409 (T>G, Asp148Glu) | Negative |
| Peng et al.2014[88] | 235(NSCLC) | DDP + PTX/GEM/NVB/PEM | BER | *OGG1* | rs1052133 (C>G, Ser326Cys) | Negative |
| Zheng et al.2017[46] | 437 in the in a discovery cohort and 781 in the validation cohort (NSCLC) | DDP/CBP + NVB/GEM/PTX/TXT/PEM | BER | *OGG1* | rs1052133 (C>G, Ser326Cys) | Negative |
| Goričar et al.2013[49] | 139(Malignant mesothelioma) | DDP + GEM/PEM Other DDP doublets | TLS | *REV3L* | rs462779 (G>A, Thr1224Ile) | Negative |
| Zheng et al.2017[46] | 437 in the in a discovery cohort and 781 in the validation cohort (NSCLC) | DDP/CBP + NVB/GEM/PTX/TXT/PEM | TLS | *REV3L* | rs462779 (G>A, Thr1224Ile) | Negative |
| Goričar et al.2013[49] | 139(Malignant mesothelioma) | DDP + GEM/PEM Other DDP doublets | TLS | *REV3L* | rs465646 (G>A) | Negative |
| Zheng et al.2017[46] | 437 in the in a discovery cohort and 781 in the validation cohort (NSCLC) | DDP/CBP + NVB/GEM/PTX/TXT/PEM | TLS | *REV3L* | rs465646 (G>A) | Negative |
| Ye et al.2015[96] | 663(NSCLC) | DDP/CBP + NVB/GEM/PTX/TXT Other DDP/CBP combinations | TLS | *REV3* | rs465646 (G>A) | *REV3* rs465646: increased risk of grade 3-4 hematologic toxicity (A/G + A/A vs. G/G OR = 2.54; 95% CI: 1.17-5.42; P = 0.016). |
| Zheng et al.2017[46] | 437 in the in a discovery cohort and 781 in the validation cohort (NSCLC) | DDP/CBP + NVB/GEM/PTX/TXT/PEM | TLS | *REV7* | rs746218 (G>A) | Negative |
| Ye et al.2015[96] | 663(NSCLC) | DDP/CBP + NVB/GEM/PTX/TXT Other DDP/CBP combinations | TLS | *REV7* | rs746218 (G>A) | Negative |
| Zheng et al.2017[46] | 437 in the in a discovery cohort and 781 in the validation cohort (NSCLC) | DDP/CBP + NVB/GEM/PTX/TXT/PEM | TLS | *REV7* | rs2233006 (T>A) | Negative |
| Ye et al.2015[96] | 663(NSCLC) | DDP/CBP + NVB/GEM/PTX/TXT Other DDP/CBP combinations | TLS | *REV7* | rs2233006 (T>A) | Negative |
| Zheng et al.2017[46] | 437 in the in a discovery cohort and 781 in the validation cohort (NSCLC) | DDP/CBP + NVB/GEM/PTX/TXT/PEM | TLS | *REV1* | rs3087386 (A>G, Phe257Ser) | Negative |
| Goričar et al.2013[49] | 139(Malignant mesothelioma) | DDP + GEM/PEM Other DDP doublets | TLS | *REV1* | rs3087386 (A>G, Phe257Ser) | *REV1* rs3087386: decreased risk of grade 2-4 neutropenia (GA + AA vs. GG OR = 0.38; 95% CI: 0.17-0.84; P = 0.017). |
| Chu et al.2016[97] | 1021(NSCLC) | DDP/CBP + NVB/GEM/PTX /TXT Other DDP/CBP combinations | TLS | *Rad18* | rs373572 (C>T, Arg302Gln) | Negative |
| Zheng et al.2017[46] | 437 in the in a discovery cohort and 781 in the validation cohort (NSCLC) | DDP/CBP + NVB/GEM/PTX/TXT/PEM | TLS | *Rad18* | rs373572 (C>T, Arg302Gln) | Negative |
| Zheng et al.2017[46] | 437 in the in a discovery cohort and 781 in the validation cohort (NSCLC) | DDP/CBP + NVB/GEM/PTX/TXT/PEM | DSB | *XRCC3* | rs861539 (C>T, Thr241Met) | Negative |
| Ruzzo et al.2014[33] | 517(Colorectal cancer) | L-OHP + 5-FU + LV L-OHP + CAP | DSB | *XRCC3* | rs861539 (C>T, Thr241Met) | Negative |
| Ludovini et al.2011[28] | 189(NSCLC) | DDP + GEM/PTX/NVB | DSB | *XRCC3* | rs861539 (C>T, Thr241Met) | Negative |
| Lee et al.2013[37] | 292(Colon cancer) | L-OHP + LV + 5-FU | DNA synthesis | *MTHFR* | rs1801131 (A1298C, Glu429Ala) | Negative |
| Ruzzo et al.2014[33] | 517(Colorectal cancer) | L-OHP + 5-FU + LV L-OHP + CAP | DNA synthesis | *MTHFR* | rs1801131 (A1298C, Glu429Ala) | Negative |
| Walia et al.2021b[35] | 123(Lung adenocarcinoma cancer) | DDP/CBP + PEM | DNA synthesis | *MTHFR* | rs1801131 (A1298C, Glu429Ala) | Negative |
| Corrigan et al.2014[42] | 136(NSCLC + Malignant mesothelioma) | DDP/CBP + PEM | DNA synthesis | *MTHFR* | rs1801131 (A1298C, Glu429Ala) | Negative |
| Kanazawa et al.2014[99] | 41(non-squamous non-small cell lung cancer) | CBP + PEM | DNA synthesis | *MTHFR* | rs1801131 (A1298C, Glu429Ala) | Negative |
| Li et al.2013[100] | 1004(NSCLC) | DDP/CBP + NVB/GEM/PTX/TXT Other DDP/CBP combinations | DNA synthesis | *MTHFR* | rs1801131 (A1298C, Glu429Ala) | Negative |
| Walia et al.2021b[35] | 123(Lung adenocarcinoma cancer) | DDP/CBP + PEM | DNA synthesis | *MTHFR* | rs1801133 (C677T, Ala222Val) | *MTHFR* rs1801133: increased risk of grade 1-3 neutropenia (CT vs. CC OR = 5.34; 95% CI: 1.49-19.06; P = 0.009, CT + TT vs. CC OR = 4.45; 95% CI: 1.28-15.43; P = 0.019). |
| Lee et al.2013[37] | 292(Colon cancer) | L-OHP + LV + 5-FU | DNA synthesis | *MTHFR* | rs1801133 (C677T, Ala222Val) | *MTHFR* rs1801133: increased risk of grade 3-4 neutropenia (TT vs. CC + CT OR = 2.32, 95 % CI 1.19-4.55, P = 0.014). |
| Li et al.2013[100] | 1004(NSCLC) | DDP/CBP + NVB/GEM/PTX/TXT Other DDP/CBP combinations | DNA synthesis | *MTHFR* | rs1801133 (C677T, Ala222Val) | *MTHFR* rs1801133: decreased risk of grade 3-4 thrombocytopenia (CT vs. CC OR = 0.40; 95% CI: 0.19-0.85; P = 0.016). |
| Ruzzo et al.2014[33] | 517(Colorectal cancer) | L-OHP + 5-FU + LV L-OHP + CAP | DNA synthesis | *MTHFR* | rs1801133 (C677T, Ala222Val) | Negative |
| Corrigan et al.2014[42] | 136(Lung cancer Malignant mesothelioma) | DDP + PEM CBP + PEM | DNA synthesis | *MTHFR* | rs1801133 (C677T, Ala222Val) | Negative |
| Kanazawa et al.2014[99] | 41(non-squamous non-small cell lung cancer) | CBP + PEM | DNA synthesis | *MTHFR* | rs1801133 (C677T, Ala222Val) | Negative |
| Isla et al.2004[54] | 62(NSCLC) | DDP + TXT | DNA synthesis | *RRM1* | rs12806698 (-37C/A) | *RRM1* rs12806698: associated with grade 2-4 leukopenia (the incident rate of grade 2-4 leukopenia for CC and CA were 31% and 10%, P = 0.05). |
| Zheng et al.2017[46] | 437 in the in a discovery cohort and 781 in the validation cohort (NSCLC) | DDP/CBP + NVB/GEM/PTX/TXT/PEM | DNA synthesis | *RRM1* | rs12806698 (-37C/A) | *RRM1* rs12806698: increased risk of grade 3-4 leukocytopenia in recessive model (OR = 5.095; 95% CI: 2.132-12.170; P = 0.0002), grade 3-4 neutropenia in recessive model (OR = 2.561; 95% CI: 1.075–6.099; P = 0.034) in discovery cohort. |
| Zheng et al.2017[46] | 437 in the in a discovery cohort and 781 in the validation cohort (NSCLC) | DDP/CBP + NVB/GEM/PTX/TXT/PEM | Apoptosis | *MDM2* | rs2279744 (309T>G) | *MDM2* rs2279744: decreased risk of grade 3-4 thrombocytopenia in additive model (OR = 0.472; 95% CI: 0.257-0.866; P = 0.015) in discovery cohort. |
| Wang et al.2014[104] | 119(SCLC) | DDP + VP-16 | Apoptosis | *MDM2* | rs2279744 (309T>G) | *MDM2* rs2279744: decreased risk of grade 3-4 neutropenia in additive model (OR = 0.48; 95% CI: 0.2652-0.8709; P = 0.015) and in recessive model (OR = 0.27; 95% CI: 0.08763-0.8859; P = 0.030) |
| Guo et al.2016[105] | 292(Lung adenocarcinoma) | DDP/CBP combinations | Apoptosis | *MDM2* | rs2279744 (309T>G) | *MDM2* rs2279744: increased risk of grade 3-4 hematologic toxicity in recessive model (OR = 2.128; 95% CI: 1.198-3.777; P = 0.010) |
| Zheng et al.2014[110] | 444(NSCLC) | DDP/CBP + GEM /VP-16 /PTX /PEM  Other DDP/CBP combinations | Apoptosis | *MDM2* | rs2279744 (309T>G) | Negative |
| Khrunin et al.2012[40] | 104(Ovarian cancer) | DDP + CTX | Apoptosis | *TP53* | rs1042522 (C>G, Pro72Arg) | *TP53* rs1042522: increased risk of grade 3-4 neutropenia (GG vs. CC + CG OR = 8.57; 95% CI: 1.05-69.8; P = 0.023). |
| Wang et al.2014[104] | 119(SCLC) | DDP + VP-16 | Apoptosis | *TP53* | rs1042522 (C>G, Pro72Arg) | *TP53* rs1042522: increased risk of grade 3-4 neutropenia in recessive model (OR = 3.44; 95% CI: 1.302-9.111; P = 0.012). |
| Guo et al.2016[105] | 292(Lung adenocarcinoma) | DDP/CBP combinations | Apoptosis | *TP53* | rs1042522 (C>G, Pro72Arg) | Negative |
| Nairuz et al.2021[39] | 180(Lung cancer) | DDP/CBP + VP-16/PTX/TXT CBP + GEM/ADM | Apoptosis | *TP53* | rs1042522 (C>G, Pro72Arg) | Negative |
| Zheng et al.2014[110] | 444(NSCLC) | DDP/CBP + GEM /VP-16 /PTX /PEM  Other DDP/CBP combinations | Apoptosis | *TP53* | rs1042522 (C>G, Pro72Arg) | Negative |
| Ludovini et al.2011[28] | 189(NSCLC) | DDP + GEM/PTX/NVB | Apoptosis | *TP53* | rs1042522 (C>G, Pro72Arg) | Negative |
| Zheng et al.2017[46] | 437 in the in a discovery cohort and 781 in the validation cohort (NSCLC) | DDP/CBP + NVB/GEM/PTX/TXT/PEM | Apoptosis | *TP53* | rs1042522 (C>G, Pro72Arg) | Negative |
| Lambrechts et al.2015[26] | 290(Ovarian cancer) | CBP + PTX CBP mono-therapy | Apoptosis | *TP53* | rs1042522 (C>G, Pro72Arg) | Negative |
| Marsh et al.2007[28] | 914 Discovery cohort and validation cohort in ratio 2:1(Ovarian cancer) | CBP + PTX/TXT | Apoptosis | *TP53* | rs1042522 (C>G, Pro72Arg) | Negative |

Abbreviations: BER: base excision repair; BLM: bleomycin; CAP: Capecitabine; CBP: Carboplatin; CI: confidence interval; CPT-11: irinotecan; CTX: cyclophosphamide; DDP: cisplatin; DSB: double-strand break repair; EC-GemCap: epirubicin cisplatin (intra-arterial infusion)-gemcitabine capecitabine; GEM: gemcitabine; L-OHP: oxaliplatin; LV: leucovorin; MMC: mitomycin C; MMR: mismatch repair; NER: nucleotide excision repair; NSCLC: non-small cell lung cancer; NVB: navelbine; OR: odds ratios; PDXG: cisplatin, docetaxel capecitabine, gemcitabine; PEM: pemetrexed; PEXG: cisplatin, epirubicin, capecitabine, gemcitabine; PTX: paclitaxel; SCLC: small cell lung cancer; TLS: translesion DNA synthesis; TXT: docetaxel; VCR: vincristine; VP-16: etoposide; 5-FU: fluorouracil.
