# Supplementary material for: Genetic polymorphisms and platinum-induced hematological toxicity: a systematic review
Source: Front Pharmacol. 2024 Aug 21;15:1445328. doi: 10.3389/fphar.2024.1445328 (PMC11371761; doi:10.3389/fphar.2024.1445328)
Supplement: Supplementary file 4 [file Table2.DOCX]

Supplementary Material

## Supplementary Table 2 Search strategy performed 28/1/2022

**Pubmed - 460 refs.**

("Pharmacogenetics"[Mesh] OR "Polymorphism, Genetic"[Mesh] OR Polymorphism[Title/Abstract] OR Polymorphisms[Title/Abstract] OR Genetic polymorphism[Title/Abstract] OR Genetic polymorphisms[Title/Abstract] OR Snp[Title/Abstract] OR Snps[Title/Abstract] OR Single nucleotide polymorphism[Title/Abstract] OR Single nucleotide polymorphisms[Title/Abstract] OR Pharmacogenomic[Title/Abstract] OR Pharmacogenomics[Title/Abstract] OR Pharmacogenetic[Title/Abstract] OR Pharmacogenetics[Title/Abstract] OR Genetic variants[Title/Abstract] OR Genetic variant[Title/Abstract] OR Genetic variation[Title/Abstract] OR Genetic variations[Title/Abstract]) AND (Toxicity[Title/Abstract] OR Toxicities[Title/Abstract] OR Side effect[Title/Abstract] OR Side effects[Title/Abstract] OR "leukopenia"[MeSH] OR leukopenia[Title/Abstract] OR leucopenia[Title/Abstract] OR "neutropenia"[MeSH] OR neutropaenia[Title/Abstract] OR neutropenia[Title/Abstract] OR anaemia[Title/Abstract] OR "anemia"[MeSH] OR anemia[Title/Abstract] OR thrombocytopaenia[Title/Abstract] OR "thrombocytopenia"[MeSH] OR thrombocytopenia[Title/Abstract] OR hematological toxicity[Title/Abstract] OR hematological toxicities[Title/Abstract] OR hematologic toxicity[Title/Abstract] OR hematologic toxicities[Title/Abstract] OR myelosuppression[Title/Abstract]) AND ("Neoplasms"[Mesh] OR Cancer[Title/Abstract] OR Cancers[Title/Abstract] OR Neoplasm[Title/Abstract] OR Neoplasms[Title/Abstract] OR Carcinoma[Title/Abstract] OR Carcinomas[Title/Abstract] OR tumor[Title/Abstract] OR tumors[Title/Abstract]) AND ("Platinum"[Mesh] OR Platinum[Title/Abstract] OR cisplatin[Title/Abstract] OR carboplatin[Title/Abstract] OR nedaplatin[Title/Abstract] OR eptaplatin[Title/Abstract] OR oxaliplatin[Title/Abstract] OR lobaplatin[Title/Abstract]).

Filter used: Humans, English

**EMBASE - 1071 refs.**

('pharmacogenetics'/exp OR 'single nucleotide polymorphism'/exp OR polymorphisms:ab,ti OR polymorphisms:ab,ti OR 'genetic polymorphism':ab,ti OR 'genetic polymorphism':ab,ti OR snp:ab,ti OR snps:ab,ti OR 'single nucleotide polymorphism':ab,ti OR 'single nucleotide polymorphism':ab,ti OR pharmacogenomic:ab,ti OR pharmacogenomics:ab,ti OR pharmacogenetic:ab,ti OR pharmacogenetics:ab,ti OR 'genetic variants':ab,ti OR 'genetic variant':ab,ti OR 'genetic variation':ab,ti OR 'genetic variations':ab,ti) AND ('toxicity'/exp OR toxicity:ab,ti OR toxicities:ab,ti OR 'side effect':ab,ti OR 'side effects':ab,ti OR leukopenia:ab,ti OR 'leukopenia'/exp OR leukopenia:ab,ti OR leucopenia:ab,ti OR 'neutropenia'/exp OR neutropaenia:ab,ti OR neutropenia:ab,ti OR 'thrombocytopenia'/exp OR thrombocytopaenia:ab,ti OR thrombocytopenia:ab,ti OR 'anemia'/exp OR anemia:ab,ti OR anaemia:ab,ti OR 'hematological toxicity':ab,ti OR 'hematological toxicities':ab,ti OR 'hematologic toxicity':ab,ti OR 'hematologic toxicities':ab,ti OR myelosuppression:ab,ti) AND ('neoplasm'/exp OR cancer:ab,ti OR cancers:ab,ti OR neoplasm:ab,ti OR neoplasms:ab,ti OR carcinoma:ab,ti OR carcinomas:ab,ti OR tumor:ab,ti OR tumors:ab,ti) AND ('platinum'/exp OR platinum:ab,ti OR cisplatin:ab,ti OR carboplatin:ab,ti OR nedaplatin:ab,ti OR eptaplatin:ab,ti OR oxaliplatin:ab,ti OR lobaplatin:ab,ti)

Filter used: Humans, English

**Web of Science - 517 refs.**

TI=(Polymorphism OR Polymorphisms OR "Genetic polymorphism" OR "Genetic polymorphisms" OR Snp OR Snps OR "Single nucleotide polymorphism" OR "Single nucleotide polymorphisms" OR Pharmacogenomic OR Pharmacogenomics OR Pharmacogenetic OR Pharmacogenetics OR "Genetic variants" OR "Genetic variant" OR "Genetic variation" OR "Genetic variations") AND TI=(Toxicity OR Toxicities OR Side effect OR Side effects OR leukopenia OR leucopenia OR neutropaenia OR neutropenia OR anaemia OR anemia OR thrombocytopaenia OR thrombocytopenia OR "hematological toxicity" OR "hematological toxicities" OR "hematologic toxicity" OR "hematologic toxicities" OR myelosuppression) AND TI=(Cancer OR Cancers OR Neoplasm OR Neoplasms OR Carcinoma OR Carcinomas OR tumor OR tumors) AND TI=(platinum OR cisplatin OR carboplatin OR nedaplatin OR eptaplatin OR oxaliplatin OR lobaplatin)

OR

AB=(Polymorphism OR Polymorphisms OR "Genetic polymorphism" OR "Genetic polymorphisms" OR Snp OR Snps OR "Single nucleotide polymorphism" OR "Single nucleotide polymorphisms" OR Pharmacogenomic OR Pharmacogenomics OR Pharmacogenetic OR Pharmacogenetics OR "Genetic variants" OR "Genetic variant" OR "Genetic variation" OR "Genetic variations") AND AB =(Toxicity OR Toxicities OR "Side effect" OR "Side effects" OR leukopenia OR leucopenia OR neutropaenia OR neutropenia OR anaemia OR anemia OR thrombocytopaenia OR thrombocytopenia OR "hematological toxicity" OR "hematological toxicities" OR "hematologic toxicity" OR "hematologic toxicities" OR myelosuppression) AND AB =(Cancer OR Cancers OR Neoplasm OR Neoplasms OR Carcinoma OR Carcinomas OR tumor OR tumors) AND AB=(platinum OR cisplatin OR carboplatin OR nedaplatin OR eptaplatin OR oxaliplatin OR lobaplatin)

OR

TS=(Polymorphism OR Polymorphisms OR "Genetic polymorphism" OR "Genetic polymorphisms" OR Snp OR Snps OR "Single nucleotide polymorphism" OR "Single nucleotide polymorphisms" OR Pharmacogenomic OR Pharmacogenomics OR Pharmacogenetic OR Pharmacogenetics OR "Genetic variants" OR "Genetic variant" OR "Genetic variation" OR "Genetic variations") AND AB =(Toxicity OR Toxicities OR "Side effect" OR "Side effects" OR leukopenia OR leucopenia OR neutropaenia OR neutropenia OR anaemia OR anemia OR thrombocytopaenia OR thrombocytopenia OR "hematological toxicity" OR "hematological toxicities" OR "hematologic toxicity" OR "hematologic toxicities" OR myelosuppression) AND AB =(Cancer OR Cancers OR Neoplasm OR Neoplasms OR Carcinoma OR Carcinomas OR tumor OR tumors) AND AB=(platinum OR cisplatin OR carboplatin OR nedaplatin OR eptaplatin OR oxaliplatin OR lobaplatin)

Filter used: English
