# Supplementary material for: Genetic polymorphisms and platinum-induced hematological toxicity: a systematic review
Source: Front Pharmacol. 2024 Aug 21;15:1445328. doi: 10.3389/fphar.2024.1445328 (PMC11371761; doi:10.3389/fphar.2024.1445328)
Supplement: Supplementary file 5 [file Table5.docx]

Supplementary Material

## Supplementary Table 5 Study characteristics

| Authors, year, | Ethnicity | Country | Platinum dose | Observation period | Toxicity criterion | Multiple testing | Statistic | Potential clinical confounders |
| --- | --- | --- | --- | --- | --- | --- | --- | --- |
| Isla et al.2004[54] | NA | NA | Cisplatin 75 mg/m2 on day 1 every 3 weeks | 1-8 cycles | 1979 WHO criteria | 0 | χ2 test | 0 |
| Han et al.2006[55] | NA | NA | Cisplatin 60 mg/m2 on Day 1 every 3 weeks Cisplatin 30 mg/m2 on Days 1 and 8 every 3 weeks | NA | CTCAE version 2.0 | 0 | χ2 test | 0 |
| KimCurran et al.2011[145] | Chinese | China | Cisplatin 75 mg/m2 or Carboplatin AUC 5, both administered on day 1 every 3 weeks | A maximum of 6 cycles | CTCAE version 3.0 | 0 | χ2 test | 0 |
| Marsh et al.2007[29] | Caucasian (Scottish) | NA | Carboplatin AUC 5 | 6 cycles | CTCAE version 2.0 | Controlling the false discovery rate at less than 10% | χ2 test | 0 |
| Tibaldi et al.2008[30] | Caucasian | NA | Cisplatin 80 mg/m2 on day 1 every 3 weeks | A maximum of 6 courses | CTCAE version 3.0 | 0 | χ2 test | 0 |
| Wang et al.2008[90] | Han Chinese | China | NA | 1-6 cycles | CTCAE version 3.0 | 0 | Logistic regression analysis | Type of chemotherapy regimens |
| Kim et al.2009[36] | Korean | NA | Carboplatin AUC 5 | 3 to 5 cycles or 6 to 9 cycles | CTCAE version 2.0 | 0 | Logistic regression analysis | Age, FIGO stage, histological type, grade, chemotherapeutic regimen and cycles of chemotherapy |
| Seo et al.2009[52] | NA | NA | Oxaliplatin 85 mg/m2 on day 1 | 4-5 cycles | CTCAE version 3.0 | 0 | χ2 test | 0 |
| Wu et al.2009[83] | Chinese | China | Cisplatin 75 mg/m2 or Carboplatin AUC 5, both administered on day 1 every 3 weeks | 2-6 cycles | CTCAE version 3.0 | Bonferroni correction | Logistic regression analysis | Performance status and type of treatment regimen |
| Chen et al.2010[53] | NA | China | Cisplatin 75 mg/m2 on day 1 every 3 weeks | At least 2 cycles | 1979 WHO criteria | 0 | χ2 test | 0 |
| Giovannetti et al.2011[26] | Caucasian | Italy | Cisplatin 30 mg/m2 or 42 mg/m2 | NA | CTCAE version 3.0 | Bonferroni correction and the Wacholder method | χ2 test | 0 |
| Han et al.2011[62] | Chinese | China | Cisplatin 75 mg/m2 or Carboplatin AUC 5, both administered on day 1 every 3 weeks | 2-6 cycles | CTCAE version 3.0 | 0 | Logistic regression analysis | Performance status and type of treatment regime |
| Ludovini et al.2011[28] | Caucasian | NA | Cisplatin 75 mg/m2 | 4-6 cycles | CTCAE version 3.0 | 0 | Logistic regression analysis | Age, performance status and type of treatment regimen |
| Zhao et al.2011[123] | Chinese | China | Cisplatin 75 mg/m2 or Carboplatin AUC 5, both administered on day 1 every 3 weeks | 2-6 cycles | CTCAE version 3.0 | Bonferroni correction | Logistic regression analysis | Data were calculated by multivariate Logistic regression with adjustment of patient characteristics with P < .1 in univariate analysis (for severe neutropenia and hematologic toxicity, the adjusting covariate was type of treatment regimen; for severe anemia, the adjusting covariate was smoking status) |
| Erčulj et al.2012[48] | NA | Slovenia | Cisplatin 75 mg/m2 or Carboplatin AUC 5 mg/mL/min Cisplatin 50 mg/m2 | 6 cycles | CTCAE version 2.0 | 0 | Logistic regression analysis | Sex, Eastern Cooperative Oncology Group (ECOG) performance status ‡2, number of first-line chemotherapy cycles, and type of first-line chemotherapy regimen |
| Gu et al.2012[115] | NA | China | Cisplatin 75 mg/m2 or Carboplatin AUC 5, both administered on day 1 every 3 weeks | 2-6 cycles | CTCAE version 3.0 | Bonferroni correction | Logistic regression analysis | Age, gender, smoking status, type of treatment regimen, TNM stage, performance status, and histological type |
| Iwata et al.2012[31] | Japanese | Japan | NA | NA | CTCAE version 4.0 | 0 | χ2 test | 0 |
| Khrunin et al.2012[40] | Eastern Slavonic origin | NA | Carboplatin AUC 5-6 | A maximum of 6 cycles | CTCAE | Bonferroni correction | Logistic regression analysis | 0 |
| Qian et al.2012[44] | Chinese | China | Cisplatin 75 mg/m2 or Carboplatin AUC 5, both administered on day 1 every 3 weeks | 2-6 cycles | CTCAE version 3.0 | Bonferroni correction | Logistic regression analysis | Gender, age at diagnosis, performance status, and type of treatment regimen |
| Xu et al.2012[58] | Han Chinese | China | Cisplatin 100 mg/m2 on day 1 | At least 2 cycles | CTCAE version 3.0 | Bonferroni correction | χ2 test | 0 |
| Zhan et al.2012[112] | Chinese | China | Cisplatin 75 mg/m2 or Carboplatin AUC 5, both administered on day 1 every 3 weeks | 2-6 cycles | CTCAE version 3.0 | 0 | Logistic regression analysis | Covariates for anemia were PS, type of treatment regimen, and TNM stage.  Covariates for other toxicities (hematologic toxicity, leukocytopenia, neutropenia, thrombocytopenia) were PS and type of treatment regimen. |
| Cortejoso et al.2013[51] | NA | Spain | NA | NA | CTCAE version 3.0 | 0 | Logistic regression analysis | Sex, performance status, and adjuvant or metastatic setting |
| Goričar et al.2013[49] | NA | Slovenia | NA | NA | CTCAE version 4.0 | 0 | Logistic regression analysis | Chemotherapy cycles |
| Lee et al.2013[37] | Korean | Korean | Oxaliplatin 85 mg/m2 on day 1 | At most 12 cycles | CTCAE version 3.0 | 0 | Logistic regression analysis | For grade 3/4 neutropenia, the covariates were cycles of chemotherapy delivered, sex, age, body surface area of the patient, stage of the tumor and Eastern Cooperative Oncology Group (ECOG) performance status. |
| Li et al.2013[100] | Chinese | China | Cisplatin 75 mg/m2 or Carboplatin AUC 5, both administered on day 1 every 3 weeks | 2-6 cycles | CTCAE version 3.0 | Benjamini-Hochberg false discovery rates (FDR q values) method | Logistic regression analysis | With adjustment of patient characteristics with P<0.05 in univariate analysis |
| Low et al.2013[19] | Japanese | Japan | NA | NA | CTCAE version 2.0 | 0 | Logistic regression analysis | 0 |
| Peng et al.2013[118] | Chinese | China | NA | NA | CTCAE version 3.0 | Bonferroni correction | Logistic regression analysis | Age, gender, PS, and type of treatment regimen |
| Corrigan et al.2014[42] | Caucasian (European ancestry) African ancestry Asian Other Not stated | UK | NA | 1-4 cycles | CTCAE version 4.0 | Bonferroni correction | Logistic regression analysis | All logistic regression models were adjusted for the first four principal components. |
| Cai et al.2014[127] | Chinese | China | Cisplatin 75 mg/m2 or Carboplatin AUC 5, both administered on day 1 every 3 weeks | 2-6 cycles | CTCAE version 3.0 | The Benjamini and Hochberg method | χ2 test | 0 |
| Chen et al.2014[120] | Chinese | China | NA | At least 2 cycles | CTCAE version 3.0 | 0 | Logistic regression analysis | Age, performance status, stage |
| Kanazawa et al.2014[99] | NA | NA | Carboplatin AUC 5 on day 1 every 3 weeks | 1-6 cycles | CTCAE version 3.0 | 0 | χ2 test | 0 |
| Peng et al.2014[88] | Han Chinese | China | Cisplatin 75 mg/m2 on day 1 every 3 weeks | At least 2 cycles | CTCAE version 3.0 | Bonferroni–Holm method | Logistic regression analysis | Age, gender, ECOG stage, histology, disease stage, smoking status, weight loss and chemotherapy regimens |
| Ruzzo et al.2014[33] | Italian | Italy | Oxaliplatin 130 mg/m2 on day 1  Oxaliplatin 85 mg/m2 on day 1 | 3-month or 6-month | CTCAE version 2.0 | 0 | Logistic regression analysis | 0 |
| Shao et al.2014[130] | Han Chinese | China | Cisplatin 75 mg/m2 or Carboplatin AUC 5, both administered on day 1 every 3 weeks | At least 2 cycles | CTCAE | 0 | Logistic regression analysis | Performance status and type of treatment regimen |
| Tan et al.2014[128] | Chinese | China | Cisplatin 75 mg/m2 or Carboplatin AUC 5, both administered on day 1 every 3 weeks | 2-6 cycles | CTCAE version 3.0 | Bonferroni correction | Logistic regression analysis | TNM and type of treatment regimen. |
| Wang et al.2014[104] | Han Chinese | China | Cisplatin 25 mg/m2 for 3 days | 4-6 cycles | CTCAE version 3.0 | 0 | Logistic regression analysis | Gender, age, BSA |
| Zhao et al.2014[121] | Han Chinese | China | Cisplatin 75 mg/m2 or Carboplatin AUC 5, both administered on day 1 every 3 weeks | 2-6 cycles | CTCAE version 3.0 | Bonferroni correction | Logistic regression analysis | Data were calculated by unconditional Logistic regression, with adjustment of patient characteristics with P < 0.1 in chi-square tests (For hematologic toxicity and neutropenia, chemotherapy regimens; for anemia, ECOG PS, Smoking status, TNM stage, and chemotherapy regimens; for thrombocytopenia, gender and chemotherapy regimens). |
| Zheng et al.2014[110] | Han Chinese | China | Cisplatin 75 mg/m2 or Carboplatin AUC 5, both administered on day 1 every 3 weeks | 2-6 cycles | CTCAE version 3.0 | Bonferroni correction | Logistic regression analysis | Sex, PS and type of chemotherapy regimen |
| Cao et al.2015[18] | Chinese | China | Cisplatin 75 mg/m2 or Carboplatin AUC 5, both administered on day 1  Oxaliplatin and Nedaplatin not mention | 2-6 cycles | CTCAE version 3.0 | 0 | Logistic regression analysis | Age, gender, smoking status, histologic type, stage and principal component |
| Chen et al.2015[108] | NA | China | NA | At least 2 cycles | CTCAE version 3.0 | 0 | Logistic regression analysis | Not mention the specific covariates. |
| Deng et al.2015[89] | Han Chinese | China | Cisplatin 75 mg/m2 on d2-4 | 2-6 cycles | CTCAE version 2.0 | 0 | Logistic regression analysis | Age, sex, smoking history, histological types, and TNM stage at entry |
| Gréen et al.2015[22] | NA | Sweden | Carboplatin AUC 5 or 6 | At least 1 cycle | CTCAE version 4.03 | Hochberg adjustment | Logistic regression analysis | 0 |
| Huang et al.2015[20] | Chinese | China | NA | NA | WHO | 0 | Logistic regression analysis | Chemotherapy regimens |
| Kalikaki et al.2015[146] | NA | NA | Cisplatin 80 mg/m2 or Carboplatin AUC 6, both administered on day 1 every 3 weeks | NA | CTCAE | 0 | χ2 test | 0 |
| Lambrechts et al.2015[27] | 99%Caucasian | Belgian | Cisplatin 100 mg/m2 on day 1 every 3 weeks | 3-6 cycles | CTCAE version 4.0 | Bonferroni correction | Logistic regression analysis | Corrected p values were obtained using a Logistic regression for the presence or absence of anemia/thrombocytopenia/febrile neutropenia while including the following covariates: genetic variant, age, BMI, AUC of carboplatin, number of administered cycles, and use of ESA for anemia or use of CSF for febrile neutropenia. In the regression for anemia, the covariate was the use of ESA. In the regression for grade 3-4 thrombocytopenia, the covariates were age and AUC of administered carboplatin. In the regression for grade 4 neutropenia, the covariates were BMI and age. |
| Qian et al.2015[109] | NA | China | NA | 2-6 cycles | CTCAE version 3.0 | calculating the false-positive report probability (FPRP) | Logistic regression analysis | Sex and chemotherapy regimen |
| Ye et al.2015[96] | Han Chinese | China | Cisplatin 75 mg/m2 or Carboplatin AUC 5, both administered on day 1 every 3 weeks | At least 2 cycles | CTCAE version 3.0 | Bonferroni correction | Logistic regression analysis | Performance status and the type of treatment regimen. |
| Yin et al.2015[125] | Chinese | China | NA | NA | CTCAE version 3.0 | 0 | Logistic regression analysis | 0 |
| Chu et al.2016[97] | Han Chinese | China | Cisplatin 75 mg/m2 or Carboplatin AUC 5, both administered on day 1 every 3 weeks | At least 2 cycles | CTCAE | 0 | Logistic regression analysis | Performance status, type of treatment regimen. |
| Fang et al.2016[111] | Han Chinese | China | NA | At least 2 cycles | CTCAE version 3.0 | false discovery rate (FDR) method | Logistic regression analysis | Age, sex, smoking status, stage, histological type and chemotherapy regimens |
| Guo et al.2016[105] | Han Chinese | China | Cisplatin 75 mg/m2 or Carboplatin AUC 5, both administered on day 1 every 3 weeks | At least 2 cycles | CTCAE version 3.0 | Bonferroni correction | Logistic regression analysis | Gender, age, PS status, TNM stage and types of chemotherapy regimen |
| Hu et al.2016[114] | Chinese | China | NA | At least 2 cycles | CTCAE version 3.0 | 0 | Logistic regression analysis | Sex, age, smoking status, stage, histological type, and chemotherapy regimens |
| Jia et al.2016[43] | Chinese | China | Cisplatin 75 mg/m2 or Carboplatin AUC 5, both administered on day 1 every 3 weeks | At least 2 cycles | CTCAE version 4.0 | 0 | Logistic regression analysis | Sex, age at diagnosis, ECOG score, BMI, TNM stages and type of treatment regimen |
| Kumpiro et al.2016[38] | Thai | Thailand | NA | At least 2 cycles | CTCAE version 4.03 | 0 | χ2 test | 0 |
| Qian et al.2016[63] | Chinese | China | NA | At least 2 cycles | CTCAE version 3.0 | 0 | Logistic regression analysis | Sex, age, smoking status, tumor histology, clinical stage, and Eastern Cooperative Oncology Group (ECOG) performance status |
| Song et al.2016[81] | Chinese | China | NA | NA | CTCAE version 3.0 | Bonferroni correction | Logistic regression analysis | Demographic and clinical factors were test against clinical outcomes by chi-square tests or log-rank test. Factors that had P-value < 0.05 were regarded as covariates. |
| Wang et al.2016[124] | Chinese | China | Cisplatin 75 mg/m2 or Carboplatin AUC 5, both administered on day 1 every 3 weeks | 2-6 cycles | CTCAE version 4.0 | Bonferroni correction | Logistic regression analysis | Data were calculated by multivariate Logistic regression with adjustment of patient characteristics with p < 0.05 in univariate analysis (the adjusting covariates for anemia were smoking status, TNM stage; the adjusting covariate for thrombocytopenia was type of chemotherapy regimens). |
| Xu et al.2016[122] | Chinese | China | NA | 2-6 cycles | CTCAE version 3.0 | 0 | Logistic regression analysis | Age, pathologic type, ECOG PS, adjuvant therapy and TNM stage |
| Yin et al.2016[45] | Chinese | China | NA | At least 2 cycles | CTCAE version 3.0 | 0 | Logistic regression analysis | Age and gender |
| Zou et al.2016[126] | NA | China | NA | At least 2 cycles | CTCAE version 3.0 | 0 | Logistic regression analysis | Variables which were associated with toxicity significantly were considered as the covariates. |
| Gong et al.2017[113] | Chinese | China | NA | At least 2 cycles | CTCAE version 3.0 | 0 | Logistic regression analysis | Age, sex, smoking status, stage, performance score (PS), platinum dose, chemotherapy interval, preventive treatment, the time of examining the blood for hematoxicity, histological type and chemotherapy regimen. |
| Liu et al.2017[116] | Chinese | China | Cisplatin 75 mg/m2 or Carboplatin AUC 5, both administered on day 1 every 3 weeks | 2-6 cycles | CTCAE version 3.0 | false discovery rate (FDR)method | Logistic regression analysis | Not mention the specific covariates. |
| Liu et al.2017[129] | Chinese | China | NA | At least 2 cycles | CTCAE version 3.0 | 0 | Logistic regression analysis | Sex, age, smoking status, tumor histology, clinical stage, and Eastern Cooperative Oncology Group (ECOG) performance status |
| Zheng et al.2017[46] | Chinese | China | Cisplatin 75 mg/m2 or Carboplatin AUC 5, both administered on day 1 every 3 weeks | 2-6 cycles | CTCAE version 4.0 | Bonferroni correction | Logistic regression analysis | Logistic regression with stepwise selection was utilized to select clinical covariates of potential effects. |
| Björn et al.2018[21] | NA | Sweden | Carboplatin AUC 5 on day 1 | At least 1 cycle | CTCAE version 4.03 | Benjamini–Hochberg adjustment | Logistic regression analysis | 0 |
| De Troia et al.2018[65] | NA | Italy | NA | NA | CTCAE version 4.0 | 0 | Logistic regression analysis | Body surface area |
| Li et al.2018[107] | Chinese | China | NA | At least 2 cycles | CTCAE version 3.0 | 0 | Logistic regression analysis | Age, sex, smoking status, histological types, tumor, node, and metastasis stage, and chemotherapeutic regimen |
| Sun et al.2018[57] | Han Chinese | China | Cisplatin 75 mg/m2 or Carboplatin AUC 5, both administered on day 1 every 3 weeks | 2-6 cycles | CTCAE version 3.0 | SNPSpD | Logistic regression analysis | Gender, age, smoking status, ECOG performance status, TNM status, histological types, and treatment regimen |
| Yoshihama et al.2018[25] | Japanese | Japan | Carboplatin AUC 5-6 every 3 weeks | 1-9 cycles | CTCAE version 4.0 | 0 | Logistic regression analysis | 0 |
| Gong et al.2019[119] | Chinese | China | NA | At least 2 cycles | CTCAE version 3.0 | 0 | Logistic regression analysis | Age, sex, stage, histological type, smoking status, and chemotherapy regimens |
| Lavanderos et al.2019[80] | Chilean | Chile | 100 Mg per BSA per cycle day | 2-5 cycles | CTCAE version 4.0 | 0 | Logistic regression analysis | The logistic multivariate models were adjusted stepwise using a forward procedure with p-value ≤ 0.2 to include potentially relevant variables in order to derive statistical association models. |
| Liblab et al.2019[47] | NA | Thailand | Cisplatin 75 mg/m2 or Carboplatin AUC 5, both administered on day 1 every 3 weeks | 6 cycles | CTCAE version 4.0 | 0 | χ2 test | 0 |
| Senk et al.2019[50] | NA | Slovenia | NA | NA | CTCAE version 4.0 | 0 | Logistic regression analysis | For grade 2-4 anemia, the covariate was CRP. For grade 1-4 thrombocytopenia, the covariate was pain at diagnosis. |
| Björn et al.2020[24] | NA | Sweden | Carboplatin AUC 5 on day 1 | At least 1 cycle | CTCAE version 4.03 | Bonferroni correction | χ2 test | 0 |
| Bushra et al.2020[74] | NA | Bangladesh | NA | NA | CTCAE version 3.0 | 0 | Logistic regression analysis | Age, sex, BMI, residence, TNM stage, and drug regimen |
| Ferracini et al.2020[41] | Non-white White | Brazil | Carboplatin was dosed at a starting area under the plasma concentration-vs. time curve (AUC) of 5–6 mg/mL/min | 1-6 cycles | CTCAE version 5.0 | 0 | Logistic regression analysis | Age, histological subtypes, and International Federation of Gynecology and Obstetrics (FIGO) |
| Nomura et al.2020[32] | Japanese | Japan | Cisplatin 70 mg/m2 on day 1 every 3 weeks | 1 cycle | CTCAE version 4.0 | 0 | Logistic regression analysis | Age, baseline ANC |
| Svedberg et al.2020[23] | NA | Sweden | Carboplatin AUC 5 on day 1 | At least 1 cycle | CTCAE version 4.03 | FDR correction | χ2 test | 0 |
| Nairuz et al.2021[39] | Bangladeshi | Bangladesh | NA | NA | CTCAE version 5.0 | 0 | Logistic regression analysis | 0 |
| Walia et al.2021a[34] | Indian | India | Cisplatin 60 mg/m2 or Carboplatin AUC 5 | 4 cycles | CTCAE version 3.0 | 0 | Logistic regression analysis | Age, gender, performance status, and chemotherapeutic regimen |
| Walia et al.2021b[35] | Indian | India | Cisplatin 70 mg/m2 | 3-6 cycles | CTCAE version 3.0 | 0 | Logistic regression analysis | Age, gender, and performance status |
| Wang et al.2021[69] | Han Chinese | China | Cisplatin 75 mg/m2 or Carboplatin AUC 5, both administered on day 1 every 3 weeks | 2-6 cycles | CTCAE version 4.0 | 0 | Logistic regression analysis | Gender, age, smoking status, ECOG performance status, TNM status, histological. |
| Zheng et al.2021[117] | Chinese | China | NA | 2-6 cycles | CTCAE version 4.0 | 0 | Logistic regression analysis | Logistic regression with stepwise selection was utilized to select clinical covariates of potential effects. |

Abbreviations: ANC: absolute neutrophil count; AUC: area under the curve; BMI: body mass Index; BSA: body surface area; CRP: C reactive protein; CSF: colony stimulating factor; CTCAE: Common Terminology Criteria for Adverse Events; ECOG: Eastern Cooperative Oncology Group; ESA: erythropoiesis stimulating agents; FIGO: International Federation of Gynecology and Obstetrics; PS: performance status; TNM: Tumor Node Metastasis.
