# Supplementary material for: Genetic polymorphisms and platinum-induced hematological toxicity: a systematic review
Source: Front Pharmacol. 2024 Aug 21;15:1445328. doi: 10.3389/fphar.2024.1445328 (PMC11371761; doi:10.3389/fphar.2024.1445328)
Supplement: Supplementary file 6 [file Table3.DOCX]

Supplementary Material

## Supplementary Table 3 Quality assessment form

| No. | Quality aspect | Grade |
| --- | --- | --- |
| 1 | Quality of clinical information | |
|  | 1. 1 point: mention the type of platinum-based chemotherapy regimens, the dosage and cycles) | 1 |
|  | 1. 1 point: adequate [description of] selection of participants, for example, providing inclusion and exclusion criteria or objective lab parameters, and baseline characteristics) | 1 |
| 2 | Quality of genotyping | |
|  | 1. 1 point: consideration of the Hardy–Weinberg equilibrium | 1 |
|  | 1. 1 point: consideration of genotyping quality, for example, by reporting percentage of successful genotyping attempts or cross validation with a different technique) | 1 |
| 3 | Quality in reporting of study population origin | |
|  | 1. 1 point: mention geographical point of sample collection | 1 |
|  | 1. 1 point: describe any methods used to assess or address population stratification | 1 |
| 4 | Quality in terms of sample size and statistical correction for multiple testing | |
|  | 1. 1 point: describe the power analysis to determined sample size | 1 |
|  | 1. 1 point: any correction for multiple testing. | 1 |
| 5 | Quality of study analysis | |
|  | 1. 1 point: there was adjustment for important potential confounders | 1 |
|  | 1. 1 point: the outcomes and outcome assessment were clearly defined; there were estimates and their precision (e.g., 95% confidence intervals) and presentation of data in the form of ORs) | 1 |

The scoring system resulted in an overall quality score of 0-10.

Abbreviations: OR: odds ratios
