# Supplementary material for: Genetic polymorphisms and platinum-induced hematological toxicity: a systematic review
Source: Front Pharmacol. 2024 Aug 21;15:1445328. doi: 10.3389/fphar.2024.1445328 (PMC11371761; doi:10.3389/fphar.2024.1445328)
Supplement: Supplementary file 7 [file Table4.docx]

Supplementary Material

## Supplementary Table 4 Overview of pharmacogenetic studies

| Authors, Year, | Sample size, Disease, Chemotherapeutic drugs | Gene, Polymorphism | Toxicity endpoint (rate) | Association | Total score |
| --- | --- | --- | --- | --- | --- |
| Isla et al.2004[54] | 62 NSCLC  DDP + TXT | *ERCC1* rs11615 (C118T, Asn118Asn) *ERCC2/XPD* rs13181 (Lys751Gln) *ERCC2/XPD* rs1799793 (Asp312Asn) *RRM1* rs12806698(-37C/A) *ABCB1/MDR1* rs1045642 (C3435T) | Grade 2-4 anemia (82%) Grade 2-4 leukopenia (74%) Grade 2-4 neutropenia (81%) Grade 2-4 thrombocytopenia | *ERCC2/XPD* rs13181 (Lys751Gln): associated with grade 2-4 neutropenia (the incident rate of grade 2-4 neutropenia for Lys/Lys, Lys/Gln and Gln/Gln were 48%, 19%, and 14%, P = 0.04). *RRM1* rs12806698 (-37C/A): grade 2-4 leukopenia (the incident rate of grade 2-4 leukopenia for CC and CA were 31% and 10%, P = 0.05). | 2 |
| Han et al.2006[55] | 107 NSCLC  DDP + CPT-11 | *ABCB1/MDR1* rs1128503 (C1236T) *ABCB1/MDR1* rs2032582 (G2677TA) *ABCB1/MDR1* rs1045642 (C3435T) *ABCC2/MRP2* rs717620 (C-24T) *ABCC2/MRP2* rs2273697 (G1249A) *ABCC2/MRP2* rs3740066 (C3972T) *ABCG2* rs2231142 (421C>A) *ABCG2* rs2231137 (34G>A) | Grade 4 neutropenia (19.85%) | *ABCB1/MDR1* rs2032582 (G2677TA): associated with grade 4 neutropenia (the incident rate of grade 4 neutropenia for GG, GT/GA and TT/TA/AA were 34.6%, 53.8%, and 11.5%, P = 0.030). | 2 |
| KimCurran et al.2011[145] | 300 NSCLC  DDP/CBP + GEM/NVB/PTX | *ERCC1* rs3212986 (C8092A) | Grade 1-3 hematologic toxicity (54.0%) | No significant association. | 4 |
| Marsh et al.2007[29] | 914 (Discovery cohort and validation cohort in ratio 2:1) Ovarian cancer CBP + PTX/TXT | *ERCC1* rs3212961 (17677G>T) *ERCC1* rs11615 (C118T, Asn118Asn) *ERCC1* rs3212986 (C8092A) *ERCC2/XPD* rs13181 (Lys751Gln) *XPCC1* rs25487 (Arg399Gln) *ABCB1/MDR1* rs1128503 (C1236T) *ABCB1/MDR1* rs2032582 (G2677TA) *ABCB1/MDR1* rs1045642 (C3435T) *ABCC2(MRP2)* rs2073337 (IVS12 148A G) *ABCC2(MRP2)* rs2273697(V417I) *ABCG2* rs2231142 (421C>A) *GSTP1* rs1695 (A313G, Ile105Val) *GSTP1* rs1138272 (c.341 C>T, Ala114Val) *MAPT* P587P *MPO* -463G A *TP53* rs1042522 (C>G, Pro72Arg) | Grade 4 neutropenic toxicity (4%) | No significant association. | 4 |
| Tibaldi et al.2008[30] | 65 NSCLC  DDP + GEM | *ERCC1* rs11615 (C118T, Asn118Asn) *ERCC2/XPD* rs13181 (Lys751Gln) *ERCC2/XPD* rs1799793 (Asp312Asn) | Grade 3-4 neutropenia Grade 3-4 thrombocytopenia Grade 3-4 anemia | No significant association. | 3 |
| Wang et al.2008[90] | 139 NSCLC+SCLC  DDP + NVB/PTX /TXT/GEM/VP-16 | *XPCC1* rs25487 (Arg399Gln) *XPCC1* rs1799782 (Arg194Trp) | Grade 3-4 hematologic toxicity (43.8%) | No significant association. | 4 |
| Kim et al.2009[36] | 118 Epithelial ovarian cancer  DDP/CBP + PTX CBP + TXT | *ERCC1* rs11615 (C118T, Asn118Asn) *ERCC1* rs3212986 (C8092A) *ERCC2/XPD* rs13181 (Lys751Gln) *XPCC1* rs25487 (Arg399Gln) *XPCC1* rs1799782 (Arg194Trp) *ABCB1/MDR1* rs1045642 (C3435T) *ABCB1/MDR1* rs2032582 (G2677TA) *GSTP1* rs1695 (A313G, Ile105Val) *GSTT1* gene deletion *GSTM1* gene deletion | Grade 3-4 hematological toxicity (73.73%) | *GSTP1* rs1695 (A313G, Ile105Val): patients with A/A genotype showed higher rate of hematological toxicity (74/94, 78.7%) than those with A/G or G/G genotype (13/24, 54.2%) (P=0.015). | 5 |
| Seo et al.2009[52] | 75 Gastric cancer  L-OHP + 5-FU + LV | *ERCC1* rs11615 (C118T, Asn118Asn) *ERCC1* rs3212986 (C8092A) *GSTP1* rs1695 (A313G, Ile105Val) *GSTT1* gene deletion *GSTM1* gene deletion | Grade 3-4 neutropenia (37.33%) | No significant association. | 3 |
| Wu et al.2009[83] | 209 NSCLC  DDP/CBP + NVB/GEM/PTX/TXT Others DDP/CBP combinations | *ERCC2/XPD* rs238406 (Arg156Arg) *ERCC2/XPD* rs1799793 (Asp312Asn) *ERCC2/XPD* rs1052555 (Asp711Asp) | Grade 3-4 hematologic toxicity (38.7%) Grade 3-4 leukocytopenia (31.9%) Grade 3-4 anemia (5.0%) Grade 3-4 thrombocytopenia (7.9%) | *XPD* rs238406 (C22541A, Arg156Arg): increased risk of grade 3-4 hematologic toxicity (AA vs. CC OR = 3.24; 95% CI: 1.35-7.78; P = 0.009), grade 3-4 leukopenia toxicity (AA vs. CC OR = 4.88; 95% CI: 1.67-14.26; P = 0.005). | 7 |
| Chen et al.2010[53] | 95 NSCLC  DDP + GEM/NVB/TXT | *ERCC1* rs11615 (C118T, Asn118Asn) *ABCB1/MDR1* rs2032582 (G2677TA) *ABCB1/MDR1* rs3213619 (-129T/C) *ABCB1/MDR1* rs1045642 (C3435T) | Grade≥1 hematologic toxicity (52.6%) | No significant association. | 3 |
| Giovannetti et al.2011[26] | 122 Pancreatic cancer  PEXG, PDXG, EC-GemCap | *ERCC1* rs11615 (C118T, Asn118Asn) *ERCC2/XPD* rs1799793 (Asp312Asn) *ERCC2/XPD* rs13181 (Lys751Gln) *XPCC1* rs25487 (Arg399Gln) | Grade 1-4 hematological toxicity (95.56%) Grade 3-4 hematological toxicity (41.11%) | No significant association. | 5 |
| Han et al.2011[62] | 445 NSCLC  DDP/CBP + NVB/GEM/PTX/TXT Other DDP/CBP combinations | *ABCC2/MRP2* rs717620 (C-24T) *ABCC2/MRP2* rs2273697 (G1249A) *ABCC2/MRP2* rs3740066 (C3972T) | Grade 3-4 hematologic toxicity (32.1%) Grade 3-4 anemia (5.3%) Grade 3-4 agranulocytosis (16.0%) Grade 3-4 leukocytopenia (24.2%) Grade 3-4 thrombocytopenia (6.2%) | *ABCC2/MRP2* rs3740066 (C3972T): increased risk of grade 3-4 thrombocytopenia (CT + TT vs. CC OR= 2.43; 95% CI: 1.06-5.56; P = 0.034). | 7 |
| Ludovini et al.2011[28] | 189 NSCLC  DDP + GEM/PTX/NVB | *ERCC1* rs11615 (C118T, Asn118Asn) *XRCC3* rs861539 (Thr241Met) *ERCC2/XPD* rs13181 (Lys751Gln) *TP53* rs1042522 (C>G, Pro72Arg) | Grade 3-4 hematologic toxicity (16.4%) | No significant association. | 6 |
| Zhao et al.2011[123] | 663 NSCLC  DDP/CBP + NVB/GEM/PTX/TXT Others DDP/CBP combinations | 16 tag SNPs in MMP-2 | Grade 3-4 hematologic toxicity (25.7%) Grade 3-4 neutropenia (13.5%) Grade 3-4 anemia (2.5%) Grade 3-4 thrombocytopenia (4.1%) | *MMP-2* rs1477017: increased risk of grade 3-4 neutropenia in additive model (OR = 1.53; 95% CI: 1.08-2.15; P = 0.016). *MMP-2* rs17301608: increased risk of grade 3-4 neutropenia in recessive model (OR = 2.11; 95% CI: 1.21-3.69; P=0.009). *MMP-2* rs12934241: increased risk of grade 3-4 neutropenia in recessive model (OR = 8.08; 95% CI: 2.83-23.06; P = 9.5*10-5c). *MMP-2* rs243847: decreased risk of grade 3-4 neutropenia in additive model (OR = 0.67; 95% CI: 0.47-0.95; P = 0.026). *MMP-2* rs243844: decreased risk of grade 3-4 neutropenia in additive model (OR = 0.67; 95% CI: 0.46-0.97; P = 0.036).  *MMP-2* rs11639960: increased risk of grade 3-4 neutropenia in additive model (OR = 1.47; 95% CI: 1.05-2.07; P = 0.026). *MMP-2* rs1992116: increased risk of grade 3-4 hematologic toxicity in additive model (OR = 1.40; 95% CI: 1.06-1.85; P = 0.017), grade 3-4 neutropenia in additive model (OR = 1.46; 95% CI: 1.02-2.09; P = 0.040), and grade 3-4 anemia in additive model (OR = 3.11; 95% CI: 1.48-6.50; P = 0.003). | 8 |
| Erčulj et al.2012[48] | 94 Malignant mesothelioma DDP/CBP + GEM/PEM DDP + MMC + VCR | *ERCC2/XPD* rs1799793 (Asp312Asn) *ERCC2/XPD* rs13181 (Lys751Gln) *ERCC1* rs11615 (C118T, Asn118Asn) *ERCC1* rs3212986 (C8092A) *GSTP1* rs1695 (A313G, Ile105Val) *GSTP1* rs1138272 (c.341 C>T, Ala114Val) *GSTM1* gene deletion *GSTT1* gene deletion | Grade 1-4 thrombocytopenia Grade 2-4 leukopenia Grade 2-4 anemia Grade 2-4 neutropenia | *ERCC2/XPD* rs1799793 (Asp312Asn): decreased risk of grade 1-4 thrombocytopenia (Asp/Asn + Asn/Asn vs. Asp/Asp OR = 0.15; 95% CI: 0.04-0.61; P = 0.008). *ERCC1* rs3212986 (C8092A): decreased risk of grade 2-4 leukopenia (CA + AA vs. CC OR = 0.18; 95% CI: 0.04-0.86; P = 0.032). *GSTM1* gene deletion: decreased risk of grade 2-4 leukopenia ( 0/0 vs. 1/1 + 1/0 OR = 0.43; 95% CI: 0.18-0.99; P = 0.048). | 6 |
| Gu et al.2012[115] | 445 NSCLC  DDP/CBP + NVB/GEM/PTX /TXT  Other DDP/CBP combinations | *BCL2* rs1801018 (A>G, Cys224Arg) *BCL2* rs1564483 (1204G>A,3´-UTR) *BCL2* rs2279115 (-938C>A) *BAX* rs4645878 (-248G>A) *CASP3* rs6948 (1296A>C, 3´-UTR) *CASP8* rs3834129 (3´-UTR) *CASP10* rs13006529 (A>T, Ile522Leu) *CASP10* rs3900115 (A>G, Ser59Ser) *TNFa* rs1800629 (-308 G>A) *MIF* rs755622 (-173 G>C) | Grade 3-4 hematologic toxicity (32.6%) | *CASP3* rs6948 (1296A>C, 3´-UTR): decreased risk of grade 3-4 hematologic toxicity (AC + CC vs. AA OR = 0.524; 95% CI: 0.333-0.824; P = 0.005). | 8 |
| Iwata et al.2012[31] | 53 Advanced carcinomas  DDP + 5-FU/GEM/TXT/VP-16/PEM/CPT-11 | *OCT2* rs316019 (808G/T, p.270Ala > Ser) *MATE1* rs2289669(G/A) | Grade 2-4 leukopenia (35.8%) Grade 2-4 thrombocytopenia (39.62%) | No significant association. | 2 |
| Khrunin et al.2012[40] | 104 Ovarian cancer  DDP + CTX | *GSTA1* rs3957357 (–69C＞T) *GSTM1* gene deletion *GSTM3* rs1799735(AGG deletion) *GSTM3* rs7483(Val224Ile) *GSTP1* rs1695 (A313G, Ile105Val) *GSTP1* rs1138272 (c.341 C>T, Ala114Val) *GSTT1* gene deletion *ERCC1* rs11615 (C118T, Asn118Asn) *ERCC1* rs3212986 (C8092A) *ERCC2/XPD* rs1799793 (Asp312Asn) *ERCC2/XPD* rs13181 (Lys751Gln) *XPCC1* rs1799782 (Arg194Trp) *XRCC1* rs2548 (Arg280His) *XPCC1* rs25487 (Arg399Gln) *TP53* 16bp duplication *TP53* rs1042522 (Arg72Pro,C＞G) *TP53* rs1625895(13494G＞A) *CYP2E1* rs17878362(96-bp insertion) *CYP2E1* rs2031920(1053C＞T) *CYP2E1* rs6413432(7632T＞A) *CYP2E1* rs2070676(9896C＞G) | Grade 3-4 neutropenia (59%) Grade 2-4 anemia (54%) Grade 1-4 thrombocitopenia (16%) | *XPCC1* rs25487 (Arg399Gln): grade 3-4 neutropenia (Arg/Arg vs. Arg/Gln + Gln/Gln OR = 3.02; 95% CI: 1.33-6.88; P = 0.009). *TP53* rs1042522 (C>G, Pro72Arg): grade 3-4 neutropenia (Pro/Pro vs. Arg/Arg + Arg/Pro OR = 8.57; 95% CI: 1.05-69.8; P = 0.023). *ERCC2/XPD* rs1799793 (Asp312Asn): grade 1-4 thrombocytopenia (Asp/Asn vs. Asp/Asp + Asn/Asn OR = 4.05; 95% CI: 1.21-13.58; P = 0.027) and grade 2-4 anemia (Asp/Asn vs. Asp/Asp + Asn/Asn OR = 2.32; 95% CI: 1.05-5.13; P = 0.048). *GSTM1* gene deletion: grade 1-4 thrombocytopenia (0/0 vs. 1/0 OR = 0.13; 95% CI: 0.03-0.62; P = 0.005), grade 2-4 anemia (0/0 vs. 1/0 OR = 0.29; 95% CI: 0.13-0.66; P = 0.003). *GSTM3* AGG deletion: grade 1-4 thrombocytopenia (AGG/AGG vs. AGG/− + −/− OR = 0.23; 95% CI: 0.07-0.71; P = 0.014) and grade 2-4 anemia (AGG/AGG vs. AGG/− + −/− OR = 0.21; 95% CI: 0.07-0.69; P = 0.007). | 4 |
| Qian et al.2012[44] | 279 (in a discovery set) and 384 (in a validation set)  NSCLC  DDP/CBP + NVB/GEM/PTX/TXT Other DDP/CBP combination | 13 tag SNPs in *CASP8* and *CASP10* | Grade 3-4 hematologic toxicity (25.7%) Grade 3-4 leukocytopenia (15.7%) Grade 3-4 agranulocytosis (13.5%) Grade 3-4 anemia (2.5%)  Grade 3-4 thrombocytopenia (4.1%) | *CASP8* rs12990906(A>G): decreased risk of grade 3-4 hematologic toxicity (GG vs. AA OR = 0.45; 95% CI: 0.26 - 0.78; P = 0.004, AG + GG vs. AA OR = 0.64; 95% CI: 0.44 - 0.94; P = 0.023). | 8 |
| Xu et al.2012[58] | 204 NSCLC  DDP + GEM/VP-16/TXT/VDS | 20 tag SNPs in *CTR1* | Grade 3-4 neutropenia (59.3%) Grade 3-4 anemia (67.2%) Grade 3-4 thrombocytopenia (86.3%) | No significant association. | 5 |
| Zhan et al.2012[112] | 445 NSCLC  DDP/CBP + NVB/GEM/PTX/TXT Others DDP/CBP combinations | Hsa-miR-196a2 rs11614913 | Grade 3-4 hematologic toxicity (29.0%) Grade 3-4 leukocytopenia (23.4%) Grade 3-4 neutropenia (14.6%) Grade 3-4 thrombocytopenia (5.8%) Grade 3-4 anemia (4.9%) | No significant association. | 6 |
| Cortejoso et al.2013[51] | 106 Colorectal cancer  L-OHP + 5-FU + LV L-OHP + CAP | *ABCB1/MDR1* rs1045642 (C3435T) *ABCB1/MDR1* rs2032582 (G2677TA) *ABCB1/MDR1* (C1236T) *XPCC1* rs25487 (Arg399Gln) *ERCC1* rs11615 (C118T, Asn118Asn) *ERCC2/XPD* rs13181 (Lys751Gln) *GSTP1* rs1695 (A313G, Ile105Val) *GSTT1* gene deletion | Grade 3-4 hematological toxicity (22.6%) Grade 3-4 anemia (0.0%) Grade 3-4 neutropenia (19.8%) Grade 3-4 neutropenia febrile (1.9%) Grade 3-4 leucopenia (2.8%) Grade 3-4 thrombocytopenia (1.9%) | *ERCC1* rs11615 (C118T, Asn118Asn): decreased risk of grade 3-4 neutropenia (CT + TT vs. CC OR = 0.205; 95% CI: 0.061-0.690; P = 0.010) | 6 |
| Goričar et al.2013[49] | 139 Malignant mesothelioma  DDP + GEM/PEM Other DDP doublets | *REV1* rs3087399 (Asn373Ser) *REV1* rs3087386 (Phe257Ser) *REV1* rs3087403 (Val138Met) *REV3L* rs455732 (Val1430Val) *REV3L* rs462779 (Thr1224Ile) *REV3L* rs3204953 (Val3064Ile)  *REV3L* rs465646 (3´-UTR) | Grade 2-4 neutropenia (34.53%) Grade 2-4 leukopenia (25.18%) Grade 2-4 anemia (46.8%) Grade 2-4 thrombocytopenia (2.2%) | *REV1* rs3087403(C>T): increased risk of grade 2-4 leukopenia (CT + TT vs. CC OR = 2.70; 95% CI: 1.19-6.12; P = 0.018). *REV1* rs3087386(A>G): decreased risk of grade 2-4 neutropenia (GA + AA vs. GG OR = 0.38; 95% CI: 0.17-0.84; P = 0.017). | 5 |
| Lee et al.2013[37] | 292 Colon cancer  L-OHP + LV + 5-FU | *MTHFR* rs1801131(A1298C) *MTHFR* rs1801133(C677T) *ERCC1* rs11615 (C118T, Asn118Asn) *ERCC1* rs3212986 (C8092A) *ERCC2/XPD* rs238406 (Arg156Arg) *ERCC2/XPD* rs1799793 (Asp312Asn) *ERCC2/XPD* rs13181 (Lys751Gln) *XPCC1* rs25487 (Arg399Gln) *XPCC1* rs1799782 (Arg194Trp) *XPCC1* rs25489 (Arg280His) *AGXT* rs4426527(A1142G) *AGXT* rs34116584(C154T)  *ABCC2/MRP2* rs3740066 (C3972T) *ABCC2/MRP2* rs717620 (C-24T) *GSTT1* gene deletion *GSTM1* gene deletion *GSTP1* rs1695 (A313G, Ile105Val) | Grade 3-4 neutropenia (60.5%) Grade 3-4 anemia (0.4%) Grade 3-4 thrombocytopenia (3.5%) Grade 3-4 febrile neutropenia (1.1%) | *MTHFR* rs1801133(C677T): increased risk of grade 3-4 neutropenia (TT vs. CC + CT OR = 2.32, 95 % CI 1.19-4.55, P = 0.014). *ERCC1* rs11615 (C118T, Asn118Asn): increased risk of grade 3-4 neutropenia (TT vs. TC + CC OR = 4.58, 95 % CI: 1.20-17.40, P = 0.026). *ABCC2/MRP2* rs717620 (C-24T): associated with grade 3-4 thrombocytopenia [5.6 % (9 out of 160 patients with CC) vs.0.8 % (1 out of 124 with CT or TT), P = 0.047]. | 6 |
| Li et al.2013[100] | 1004 NSCLC  DDP/CBP + NVB/GEM/PTX/TXT Other DDP/CBP combinations | 10 tagging and potentially functional SNPs in *MTHFR* | Grade 3-4 hematologic toxicity (23.7%) Grade 3-4 neutropenia (12.3%) Grade 3-4 leucopenia (15.2%) Grade 3-4 anemia (3.1%) Grade 3-4 thrombocytopenia (3.6%) | *MTHFR* rs1537514(G>C): increased risk of grade 3-4 thrombocytopenia (CC vs. GG OR = 9.34; 95% CI: 1.75-49.72; P = 0.009).  *MTHFR* rs1801133(C677T): decreased risk of grade 3-4 thrombocytopenia (AG vs. GG OR = 0.40; 95% CI: 0.19-0.85; P = 0.016). | 8 |
| Low et al.2013[19] | 1171 Carcinomas  DDP/CBP-based chemotherapy | GWAS | Grade 3-4 neutropenia/leucopenia | SNPs showing the most significant association with chemotherapy-induced severe neutropenia ⁄ leucopenia are: rs4886670 (Pmin = 9.86 910 7, OR = 1.61, 95% CI = 1.33–1.94) near *RPL36AP45* for (i); rs10253216 (Pmin = 1.68 9 10 7, OR = 1.48, 95% CI =1.16–1.89) near *AGR2* for (ii); and rs11071200 (Pmin = 8.51 9 10 7, OR = 8.24, 95% CI = 2.89–23.5) on PRTG. | 5 |
| Peng et al.2013[118] | 663 NSCLC  DDP/CBP + NVB/GEM/PTX/TXT Other DDP/CBP combinations | *VCP* rs1053318 *VCP* rs2074549 *VCP* rs514492 | Grade 3-4 hematologic toxicity (25.7%) Grade 3-4 neutropenia (13.5%) Grade 3-4 anemia (2.5%) Grade 3-4 thrombocytopenia (4.1%) | *VCP* rs2074549: grade 3-4 neutropenia in recessive model (OR = 2.863; 95% CI: 1.564-5.241; P = 0.001). | 7 |
| Corrigan et al.2014[42] | 136 NSCLC or Malignant mesothelioma  DDP/CBP + PEM | *MTHFR* rs1801131(A1298C) *MTHFR* rs1801133(C677T) *ERCC2/XPD* rs13181 (Lys751Gln) | Grade 3-4 hematologic toxicity (25.8%) Grade 3-4 neutropaenia (17.4%) | No significant association. | 8 |
| Cai et al.2014[127] | 663 NSCLC DDP/CBP + NVB/GEM/PTX/VP-16/BEV | 14 SNPs of *CDC25A*, *CDC25B* and *CDC25C* | Grade 3-4 hematology toxicity (25.4%) | *CDC25B* rs3761218: associated with grade 3-4 hematology toxicity (the incident rate of grade 3-4 hematology toxicity for AA, AG and GG were 23.48%, 24.14%, and 41.54%, P = 0.008). | 6 |
| Chen et al.2014[120] | 412 NSCLC + SCLC  DDP/CBP + GEM/PTX/NVB/VP-16/CPT-11 | 28 SNPs in *WISP1* | Grade 3-4 hematologic toxicity (22.1%) | *WISP1* rs16904853: decreased risk of grade 3-4 hematologic toxicity in recessive model (OR = 0.45; 95% CI: 0.23-0.89; P = 0.021). *WISP1* rs2929970: decreased risk of grade 3-4 hematologic toxicity in recessive model (OR = 0.34; 95% CI: 0.13-0.89; P = 0.028). *WISP1* rs2977549: decreased risk of grade 3-4 hematologic toxicity in recessive model (OR = 0.39; 95% CI: 0.17-0.88; P = 0.024)  *WISP1* rs2977551: decreased risk of grade 3-4 hematologic toxicity in recessive model (OR = 0.43; 95% CI: 0.19-0.99; P = 0.048) | 6 |
| Kanazawa et al.2014[99] | 41 Non-squamous non-small cell lung cancer  CBP + PEM | *MTHFR* rs1801131(A1298C) *MTHFR* rs1801133(C677T) | Grade 3-4 leukopenia (19.5%) Grade 3-4 neutropenia (29.3%) Grade 3-4 anemia (34.1%) Grade 3-4 thrombocytopenia (17.1%) | No significant association. | 4 |
| Peng et al.2014[88] | 235 NSCLC  DDP + PTX/GEM/NVB/PEM | *OGG1* rs1052133 (Ser326Cys) *APE1* rs1130409 (Asp148Glu) *APE1* rs1760944 (-141T/G) *XPCC1* rs25487 (Arg399Gln) | Grade 3-4 hematologic toxicity (38.7%) | *XPCC1* rs25487: grade 3-4 hematologic toxicity (GA + AA vs. GG OR = 2.135; 95% CI: 1.207-3.777; P = 0.009). | 7 |
| Ruzzo et al.2014[33] | 517 Colorectal cancer  L-OHP + 5-FU + LV L-OHP + CAP | *MTHFR* rs1801131(A1298C) *MTHFR* rs1801133(C677T) *ERCC1* rs11615 (C118T, Asn118Asn) *XPCC1* rs25487 (Arg399Gln) *ERCC2/XPD* rs1799793 (Asp312Asn) *ERCC2/XPD* rs13181 (Lys751Gln) *XRCC3* rs861539 (Thr241Met) *GSTP1* rs1695 (A313G, Ile105Val) *GSTT1* gene deletion *GSTM1* gene deletion *ABCC2/MRP2* rs3740066 (C3972T) *ABCC2* rs1885301 *ABCC2* rs4148386 | Grade 3-4 neutropenia (29.0%) | No significant association. | 7 |
| Shao et al.2014[130] | 663 NSCLC  DDP/CBP + NVB/GEM/PTX/TXT Other DDP/CBP combinations | 173 SNPs in *POLK* | Grade 3-4 hematological toxicity (25.7%) Grade 3-4 anemia (2.5%) Grade 3-4 agranulocytosis (13.5%) Grade 3-4 leukocytopenia (15.7%) Grade 3-4 thrombocytopenia (4.1%) | *POLK* rs3756558: grade 3-4 hematological toxicity in additive model (OR = 0.71; 95% CI: 0.50-0.98; P = 0.042). | 6 |
| Tan et al.2014[128] | 1004 NSCLC  DDP/CBP + NVB/GEM/PTX/TXT Others DDP/CBP combinations | 40 SNPs in *MIF*, *JAB1*, *SKP1*, *CUL1*, *RBX1*, *NEDD8*, *CAND1*, and *CD74* | Grade 3-4 hematologic toxicity (23.7%) Grade 3-4 neutropenia (12.3%) Grade 3-4 leucopenia (15.2%) Grade 3-4 anemia (3.1%) Grade 3-4 thrombocytopenia (3.6%) | *MIF* rs4822443(G>A): increased risk of grade 3-4 hematologic toxicity (AG vs. GG OR = 1.42; 95% CI: 1.04-1.94; P < 0.05) *MIF* rs4822446(A>G): increased risk of grade 3-4 hematologic toxicity (AG vs. AA OR = 1.39; 95% CI: 1.02-1.90; P < 0.05) *MIF* rs12485068(A>G): increased risk of grade 3-4 hematologic toxicity (AG vs. AA OR = 1.42; 95% CI: 1.04-1.94; P < 0.05) *CD74* rs2748249(C>A): increased risk of grade 3-4 hematologic toxicity (CA vs. CC OR = 1.72; 95% CI: 1.24-2.39; P < 0.001)  *CD74* rs1560661(G>A): decreased risk of grade 3-4 hematologic toxicity (GG vs. AA OR = 0.42; 95% CI: 0.25-0.70; P < 0.001) | 7 |
| Wang et al.2014[104] | 119 SCLC  DDP + VP-16 | *MDM2* rs2279744 (309T>G) *TP53* rs1042522 (C>G, Pro72Arg) | Grade 3-4 neutropenia (30.25%) | *MDM2* rs2279744: grade 3-4 neutropenia in additive model (OR = 0.48; 95% CI: 0.2652-0.8709; P = 0.015) and in recessive model (OR = 0.27; 95% CI: 0.08763-0.8859; P = 0.030) *TP53* rs1042522: grade 3-4 neutropenia in recessive model (OR = 3.44; 95% CI: 1.302-9.111; P = 0.012) | 6 |
| Zhao et al.2014[121] | 1004 NSCLC  DDP/CBP + NVB/GEM/PTX/TXT Others DDP/CBP combinations | 7 tag-SNPs in *TERT* | Grade 3-4 hematologic toxicity (23.9%) Grade 3-4 neutropenia (12.3%) Grade 3-4 anemia (3.1%) Grade 3-4 thrombocytopenia (3.6%) | No significant association | 8 |
| Zheng et al.2014[110] | 444 NSCLC  DDP/CBP + GEM /VP-16 /PTX /PEM  Other | *TP53* rs1042522 (C>G, Pro72Arg) *MDM2* rs2279744 (309T>G) *MDM2* rs937282 (c.-461C>G) | Grade 3-4 hematologic toxicity (32.2%) | *MDM2* rs937282: grade 3-4 hematologic toxicity (CG vs. GG OR = 1.121; 95% CI: 1.062-1.184; P = 0.026). | 7 |
| Cao et al.2015[18] | 333 (in discovery cohort) and 876 (in validation cohort) NSCLC  DDP/CBP + GEM/PTX/TXT | 588 732 SNPs | Grade 3-4 myelosuppression (18.6%) | Rs13014982 at 2q24.3 and rs9909179 at 17p12 exhibited consistently significant associations with myelosuppression risk in both the genome-wide association studies (GWAS) scan and the replication stage | 7 |
| Chen et al.2015[108] | 317 NSCLC + SCLC  DDP/CBP + GEM/PTX/NVB/VP-16/CPT-11 | 60 SNPs in *ABCB1*, *ABCG2*, *AQP2*, *AQP9*, *MVP*, *OCT2*, *SIRT1*, SLC2A1, TMEM205, *HMGB2*, *RPA1*, *SSRP1*, *XPA*, and *XRCC5* | Grade 3-4 hematologic toxicity (32.2%) | *XRCC5* rs1051685: decreased risk of grade 3-4 hematologic toxicity in additive model (OR = 0.37; 95% CI: 0.14-0.96; P = 0.041) and dominant model (OR = 0.37; 95% CI: 0.14-0.97; P = 0.043). *XRCC5* rs6941: increased risk of grade 3-4 hematologic toxicity in additive model (OR = 1.93; 95% CI: 1.21-3.10; P = 0.006) and dominant model (OR = 2.05; 95% CI: 1.19-3.53; P = 0.001). *AQP2* rs10875989: increased risk of grade 3-4 hematologic toxicity in additive model (OR = 1.53; 95% CI: 1.06-2.21; P = 0.025) and dominant model (OR = 1.83; 95% CI: 1.03-2.07; P = 0.039). | 6 |
| Deng et al.2015[89] | 97 NSCLC  DDP + GEM/NVB/PTX/TXT | *XPCC1* rs25487 (Arg399Gln) *GSTP1* rs1695 (A313G, Ile105Val) *ATP7A* C2299G | Grade 1-4 lymphopenia (26.8%) Grade 1-4 leukopenia (30.9%) Grade 1-4 neutropenia (25.8%)  Grade 1-4 thrombocytopenia (7.2%) Grade 1-4 anemia (10.3%) | *XRCC1* rs25487(G23885A, Arg399Gln): decreased risk of grade 1-4 lymphopenia (AG + AA vs. GG OR = 0.323; 95% CI: 0.121-0.862; P = 0.024). | 5 |
| Gréen et al.2015[22] | 32(in discovery cohort) and 291(in validation cohort) NSCLC  CBP + GEM | Whole-Exome Sequencing | In the discovery data set grade 3-4 neutropenia (50%) grade 3-4 thrombocytopenia (50%) | We identified rs1453542 in *OR4D6* (P=0.0008; OR = 5.2; 95% CI, 1.8–18) as a marker for gemcitabine/carboplatin-induced neutropenia and rs5925720 in *DDX53* (OR = 0.36; 95% CI: 0.17–0.71; P =0.0015;) as a marker for thrombocytopenia. | 7 |
| Huang et al.2015[20] | 286 Cervical cancer  DDP/CBP + taxanes/CPT-11 | 657178 SNPs | Grade 2-4 neutropenia (20.63%) | 32 variants associated with neutropenia in the patients after chemotherapy were found (P<1 × 10-4). During internal validation and external validation, artificial neural network performed well in predicting neutropenia with considerable accuracy, which is 88.9% and 81.7% respectively. | 6 |
| Kalikaki et al.2015[146] | 107 NSCLC  DDP/CBP + PTX/GEM  DDP + TXT/NVB | *ERCC1* rs11615 (C118T, Asn118Asn) *ERCC1* rs3212986 (C8092A) *ERCC2/XPD* rs13181 (Lys751Gln) *ERCC2/XPD* rs1799793 (Asp312Asn) *XPCC1* rs25487 (Arg399Gln) | Grade 3-4 hematologic toxicities (35.92%) | No significant association | 4 |
| Lambrechts et al.2015[27] | 290 Ovarian cancer  CBP + PTX CBP mono-therapy | *ABCB1/MDR1* rs1128503 (C1236T) *ABCB1/MDR1* rs1045642 (C3435T) *ABCC2/MRP2* rs2073337(c.1668+148A>G) *ABCC2/MRP2* rs12762549 (*+9383C>G) *TP53* rs1042522 (C>G, Pro72Arg) *GSTP1* rs1695 (A313G, Ile105Val) *GSTP1* rs1138272 (c.341 C>T, Ala114Val) *ERCC1* rs11615 (C118T, Asn118Asn) *ERCC1* rs3212961 (17677G>T) *ERCC2/XPD* rs1799793 (Asp312Asn) | Grade 3-4 anemia (19.7%) Grade 3-4 thrombocytopenia (19.7%) Grade 4 neutropenia (69.7%), Grade 3-4 febrile neutropenia (7.9%) | *ABCB1/MDR1* rs1128503 (C1236T): increased risk of grade 3-4 anemia in additive model (OR = 1.71; 95% CI: 1.07-2.71; P = 0.023). *ABCC2/MRP2* rs12762549 (*+9383C>G): decreased risk of grade 3-4 anemia in additive model(OR = 0.51; 95% CI: 0.33-0.81; P = 0.004). *ERCC1* rs11615 (C118T, Asn118Asn): increased risk of grade 3-4 anemia in additive model(OR = 1.61; 95% CI: 1.04-2.50; P = 0.031). | 8 |
| Qian et al.2015[109] | 663 NSCLC  DDP/CBP + NVB/GEM/PTX/TXT Other combinations | *MDM2* rs3730488 *MDM2* rs1690924 *MDM2* rs1470383 *MDM2* rs3730581 *MDM2* rs3730635 | Grade 3-4 hematologic toxicity (25.6%) | *MDM2* rs1470383(G>A): grade 3-4 hematologic toxicity (AG vs. AA OR = 4.10; 95% CI: 1.73-9.71; P = 0.001, GG vs. AG + AA OR = 4.08; 95% CI: 1.73-9.58; P = 0.001). | 7 |
| Ye et al.2015[96] | 663 NSCLC  DDP/CBP + NVB/GEM/PTX/TXT Others DDP/CBP combinations | 9 SNPs in *REV3* and 4 SNPs in *REV7* | Grade 3-4 hematological toxicity (25.7%) Grade 3-4 anemia (2.5%) Grade 3-4 agranulocytosis (13.5%) Grade 3-4 leukocytopenia (15.7%) Grade 3-4 thrombocytopenia (4.1%) | *REV3* rs240966(A>G): grade 3-4 hematologic toxicity (A/G+G/G vs. A/A OR = 0.44; 95% CI: 0.21-0.94; P = 0.03). *REV3* rs4945880(G>A): grade 3-4 hematologic toxicity (A/G+A/A vs. G/G OR = 1.50; 95% CI: 1.05-2.15; P = 0.025). *REV3* rs465646(G>A): grade 3-4 hematologic toxicity (A/G+A/A vs. G/G OR = 2.54; 95% CI: 1.17-5.42; P = 0.016). *REV7* rs2233025(G>A): grade 3-4 hematologic toxicity (A/G+G/G vs. A/A OR = 0.29; 95% CI: 0.10-0.82; P = 0.018). | 7 |
| Yin et al.2015[125] | 325 NSCLC  DDP/CBP + GEM /VP-16 /PTX /PEM  Other | 10 SNPs in *eIF3a* | Grade 1-4 neutropenia (65.54%) Grade 1-4 anemia (64.92%) Grade 1-4 thrombocytopenia (64.92%) | *eIF3a* rs1409314, rs4752219, rs4752220 and rs7091672 associated with increased risk of grade 1-4 neutropenia, anemia, thrombocytopenia. | 5 |
| Chu et al.2016[97] | 1021 NSCLC  DDP/CBP + NVB/GEM/PTX /TXT Other DDP/CBP combinations | 10 tag SNP in *Rad18* | Grade 3-4 hematological toxicity (28.1%) Grade 3-4 anemia (2.0%) Grade 3-4 agranulocytosis (11.4%) Grade 3-4 leukocytopenia (15.6%) Grade 3-4 thrombocytopenia (4.6%) | No association in the whole population *RAD18* rs586014(A>G): decreased risk of grade 3-4 hematological toxicity in non-smoker (AG + GG vs. AA OR = 0.48; 95% CI: 0.26-0.88; P = 0.015). *RAD18* rs654448(G>A): increased risk of grade 3-4 hematological toxicity in non-smoker (AA + AG vs. GG OR = 2.13; 95% CI: 1.05-4.27; P = 0.033). *RAD18* rs9880051(G>A): increased risk of grade 3-4 hematological toxicity in non-smoker (AA + AG vs. GG OR = 1.95; 95% CI: 1.11-3.49; P = 0.021) and decreased risk of grade 3-4 leukocytopenia toxicity in smoker (AA + AG vs. GG OR = 0.39; 95% CI: 0.22-0.70; P = 0.002). *RAD18* rs6763823(G>A): decreased risk of grade 3-4 leukocytopenia toxicity in smoker (AA + AG vs. GG OR = 0.60; 95% CI: 0.38-0.95; P = 0.031). | 6 |
| Fang et al.2016[111] | 408 NSCLC + SCLC  DDP/CBP + NVB/GEM/TXT/VP-16 | miR-605 rs2043556 miR-146a rs2910164 miR-149 rs71428439 miR-196a-2 rs11614913 miR-27a rs895819 miR-499 rs3746444 miR-30c-1 rs928508 miR-5197 rs2042253 | Grade 3-4 hematologic toxicity (23.77%) | No association in the whole population. In SLCL patents, miR-5197 rs2042253: grade 3-4 hematologic toxicity in dominant model (OR = 8.7; 95% CI: 1.69-44.78; P = 0.010). In cisplatin-based treatment miR-5197 rs2042253: grade 3-4 hematologic toxicity in additive model (OR = 1.52; 95% CI: 1.02-2.25; P = 0.037) and dominant model (OR = 1.99; 95% CI: 1.12-3.53; P = 0.019). | 7 |
| Guo et al.2016[105] | 292 Lung adenocarcinoma  DDP/CBP combinations | TP53 rs1042522 (C>G, Pro72Arg) MDM2 rs2279744 (309T>G) | Grade 3-4 hematologic toxicity (34.93%) | MDM2 rs2279744 (309T>G): increased risk of grade 3-4 hematologic toxicity in recessive model (OR = 2.128; 95% CI: 1.198-3.777; P = 0.010) | 5 |
| Hu et al.2016[114] | 467 NSCLC+SCLC  DDP/CBP + GEM/VP-16/PEM/TXT /PTX /CPT-11/NVB | CASC8 rs10505477 | Grade 3-4 hematologic toxicity (24.3%) | No significant association in overall subjects. CASC8 rs10505477: decreased risk of grade 3-4 hematologic toxicity in NSCLC in additive model (OR = 0.62; 95% CI: 0.43-0.90; P = 0.01). | 6 |
| Jia et al.2016[43] | 345 (in discovery group) and 344 (in replication group) NSCLC  DDP/CBP + PEM/TXT/PTX/GEM | 11 selected, independent, potentially functional SNPs in *GADD45A*, *GADD45B*, *GADD45G*, *MAP2K7*, *MAP2K4*, *MAP3K4*, *MAPK8*, *MAPK9* and *MAPK14* | Grade2-4 leukopenia (39.4%) Grade2-4 neutropenia (52.3%) Grade2-4 thrombocytopenia (15.6%) Grade2-4 anemia (56.0%) | *GADD45B* rs2024144(C>T): increased risk of grade 3-4 hematologic toxicity in discovery group (CT + TT vs. CC OR = 1.80; 95% CI: 1.01-3.18; P = 0.046). *GADD45B* rs2024144(C>T): increased risk of grade 3-4 hematologic toxicity in replication group (CT + TT vs. CC OR = 1.87; 95% CI: 1.02-3.42; P = 0.042). *GADD45B* rs2024144(C>T): increased risk of grade 3-4 hematologic toxicity in all patients (CT + TT vs. CC OR = 1.80; 95% CI: 1.19-2.71; P = 0.005). | 6 |
| Kumpiro et al.2016[38] | 32 NSCLC CBP + GEM | *CTR1* rs12686377 | Grade 1-4 anemia (86.67%) Grade 1-4 thrombocytopenia (50%) Grade 1-4 neutropenia (53.33%) | No significant association. | 3 |
| Qian et al.2016[63] | 403 NSCLC  DDP/CBP + GEM/PEM/PTX/TXT/NVB | *OCT2* rs316003 *OCT2* rs316019 (808G/T, p.270Ala > Ser) *ABCB1/MDR1* rs1045642 (C3435T) *ABCC2/MRP2* rs717620 (C-24T) *ABCC2/MRP2* rs2273697 (G1249A) *ABCC2/MRP2* rs3740066 (C3972T) *MATE1* rs2289669 | Grade 3-4 hematologic toxicity (23.6%) | *OCT2* rs316019 (808G/T, p.270Ala > Ser): grade 3-4 hematological toxicity in additive model (OR = 0.58; 95% CI: 0.34-0.97; P = 0.039). *MATE1* rs2289669: grade 3-4 hematological toxicity in recessive model (OR = 1.92; 95% CI: 1.13-3.25; P = 0.016). | 6 |
| Song et al.2016[81] | 1004 NSCLC  DDP/CBP + NVB/GEM/PTX/TXT Others DDP/CBP combinations | 173 SNPs in 27 genes in NER pathway (XPC, RAD23B, ERCC2, GTF2H1, XPA, ERCC5, ERCC1, ERCC4, ERCC8, ERCC, DDB2, LIG1, CDK7, CCNH, MNAT1, RPA1, RPA2, RFC1, RFC2, POLD1, POLD2, POLD3, POLD4, POLE, POLE2, GTF2H3, GTF2H4) | Grade 3-4 anemia (3.1%) Grade 3-4 neutropenia (12.3%) Grade 3-4 trombocytopenia (3.6%) | No SNPs satisfied the significant level of bonferroni correction *GTF2H1* rs4150558: grade 3-4 anemia (OR = 2.74; 95% CI: 1.23-6.09; P = 0.013). *POLD3* rs10857: grade 3-4 neutropenia (OR = 0.55; 95% CI: 0.39-0.76; P = 3.01×10−4). *POLD3* rs6592576: grade 3-4 neutropenia (OR = 0.56; 95% CI: 0.41-0.77; P = 3.58×10−4).  *RPA1* rs12727: grade 3-4 thrombocytopenia (OR = 1.81; 95% CI: 1.02-3.21; P = 0.044). *POLD1* rs3219281: grade 3-4 thrombocytopenia (OR = 1.87; 95% CI: 1.4-3.34; P = 0.035). *POLD1*: rs3219341: grade 3-4 thrombocytopenia (OR = 1.84; 95% CI: 1.03-3.26; P = 0.039). *POLD1*: rs1726801: grade 3-4 thrombocytopenia (OR = 1.86; 95% CI: 1.05-3.30; P = 0.033). | 5 |
| Wang et al.2016[124] | 1004 NSCLC DDP/CBP + NVB/GEM/PTX/TXT Others DDP/CBP combinations | 10 tag SNPs in RICTOR | Grade 3-4 hematologic toxicity (23.9%) Grade 3-4 anemia (3.1%) Grade 3-4 neutropenia (12.3%) Grade 3-4 thrombocytopenia (3.6%) | *RICTOR* rs7703002: grade 3-4 anemia (AC vs. CC OR = 2.55; 95% CI: 1.17–5.54; P = 0.018, AA + AC vs. CC OR = 2.45; 95% CI: 1.16–5.30; P = 0.020). *RICTOR* rs4321771: grade 3-4 thrombocytopenia (AG vs. GG OR = 2.84; 95% CI: 1.25-6.46; P = 0.013, AA + GA vs. GG OR = 2.75; 95% CI: 1.21-6.25; P = 0.016). | 8 |
| Xu et al.2016[122] | 272 female patients NSCLC  DDP/CBP + NVB/GEM/PTX/TXT Others DDP/CBP combinations | *CHEK2* rs4035540 *CHEK2* rs5762746 *CHEK2* rs2236141 *CHEK2* rs2236142 | Grade 3-4 hematological toxicity (27.1%) Grade 3-4 leukotoxicity (16.8%) | No significant association | 6 |
| Yin et al.2016[45] | 190 (in Derivation cohort) and 200 (in Derivation cohort) NSCLC  DDP/CBP + GEM/PEM/PTX/TXT/NVB/VP-16 | 416 SNPs in 185 genes | Grade 3-4 hematological toxicity (22.76%) in derivation cohort Grade 3-4 hematological toxicity (25.00%) in validation cohort | The hematological toxicity prediction model achieved a sensitivity of 0.89 and a specificity of 0.39 with the ROC AUC of 0.76. | 5 |
| Zou et al.2016[126] | 317 NSCLC+SCLC  DDP/CBP + GEM/PTX/NVB/VP-16/CPT-11 | 19 tagSNPs in HSPA4, HSPB1, HSPE1, RAC1, RhoA. | Grade 3-4 hematologic toxicity (23.7%) | *RAC1* rs836554: increased risk of grade 3-4 hematologic toxicity in age >55 subgroup in recessive model (OR = 3.32; 95% CI: 1.23-8.99; P = 0.018). *RAC1* rs836554: increased risk of grade 3-4 hematologic toxicity in NSCLC subgroup in recessive model (OR = 3.50; 95% CI: 1.37-8.99; P = 0.009). *RAC1* rs4720672: increased risk of grade 3-4 hematologic toxicity in age ≤55 subgroup in dominant model (OR = 2.73; 95% CI: 1.15-6.47; P = 0.023). *RAC1* rs12536544: decreased risk of grade 3-4 hematologic toxicity in female subgroup in additive model (OR = 0.24; 95% CI: 0.07-0.78; P = 0.018) and in dominant model (OR = 0.14; 95% CI: 0.04-0.57; P = 0.006) in NSCLC subgroup in additive model (OR = 0.56; 95% CI: 0.33-0.95; P = 0.032) in non-smoker subgroup in dominant model (OR = 0.38; 95% CI: 0.15-0.96; P = 0.041). | 6 |
| Gong et al.2017[113] | 467 NSCLC + SCLC  DDP/CBP + PEM/GEM/PTX /TXT/VP-16 Other DDP/CBP-based chemotherapy (CPT-11 + DDP/CBP, NVB +DDP/CBP) | 14 potentially functional polymorphisms within 8 lncRNAs (*HOTTIP*, *HOTAIT*, *H19*, *ANRIL*, *CCAT2*, *MALAT1*, *MEG3*, and *POLR2E*) | Grade 3-4 hematological toxicity (24.4%) | No association in the whole population. | 6 |
| Liu et al.2017[116] | 555 Lung adenocarcinoma  DDP/CBP + PTX/TXT/NVB /VP-16/BEV | 7 tagSNPs in *CASP8* | Grade 3-4 neutropenia (12.6%) Grade 3-4 thrombocytopenia (4.15%) Grade 3-4 anemia | *CASP8* rs7608692(G>A): decreased risk of grade 3-4 neutropenia (AG vs. GG OR = 0.51; 95%CI: 0.28-0.92; P = 0.024). | 6 |
| Liu et al.2017[129] | 220 NSCLC+SCLC  DDP/CBP + GEM/PEM/PTX/TXT/NVB | 44 tag SNP of *MLH1*, *MSH2*, *MSH3*, *MSH4*, *MSH5*, and *MSH6*. | Grade 3-4 hematologic toxicity (25.0%) | *MSH3* rs6151627: increased risk of grade 3-4 hematologic toxicity in dominant model (OR = 2.38; 95% CI: 1.23-4.60; P = 0.010).  *MSH3* rs6151670: increased risk of grade 3-4 hematologic toxicity in dominant model (OR = 2.05; 95% CI: 1.07-3.93; P = 0.031).  *MSH3* rs7709909: increased risk of grade 3-4 hematologic toxicity in dominant model (OR = 2.38; 95% CI: 1.23-4.64; P = 0.010).  *MSH5* rs805304: increased risk of grade 3-4 hematologic toxicity in dominant model (OR = 1.99; 95% CI: 1.01-3.90; P = 0.047). | 6 |
| Zheng et al.2017[46] | 437 (in the discovery cohort) and 781 (in the validation cohort) NSCLC  DDP/CBP + NVB/GEM/PTX/TXT/PEM | 97 SNPs in 54 candidate genes responsible for repairing DNA interstrand and intrastrand cross-links | In a discovery cohort  Grade 3-4 hematologic toxicity (24.0%) Grade 3-4 leukocytopenia (10.1%) Grade 3-4 neutropenia (16.0%) Grade 3-4 thrombocytopenia (5.9%) Grade 3-4 anemia (7.3%%) in validation cohort Grade 3-4 hematologic toxicity (24.6%) Grade 3-4 leukocytopenia (16.8%) Grade 3-4 neutropenia (12.3%) Grade 3-4 thrombocytopenia (3.2%) Grade 3-4 anemia (3.3%) | In discovery corhort, *ERCC1* rs3212986: decreased risk of grade 3-4 hematologic toxicity in recessive model (OR = 0.326; 95% CI: 0.123-0.861; P = 0.024).  *ERCC1* rs11615: increased risk of grade 3-4 anemia in dominant model (OR = 2.230; 95% CI: 1.041-4.775; P = 0.039). *RRM1* rs12806698: increased risk of grade 3-4 leukocytopenia in recessive model (OR = 5.095; 95% CI: 2.132-12.170; P = 0.0002), grade 3-4 neutropenia in recessive model (OR = 2.561; 95% CI: 1.075-6.099; P = 0.034). *XPC* rs2228001: increased risk of grade 3-4 leukocytopenia in dominant model (OR = 2.217; 95% CI: 1.054-4.665; P = 0.036). *XPC* rs2228000: decreased risk of grade 3-4 hematologic toxicity in dominant model (OR = 0.515; 95% CI: 0.324-0.820; P = 0.005) , grade 3-4 leukocytopenia in dominant model (OR = 0.432; 95% CI: 0.226-0.825; P = 0.011), grade 3-4 neutropenia in additive model (OR = 0.561; 95% CI: 0.365-0.862; P = 0.008). *XPF* rs1799801: increased risk of grade 3-4 hematologic toxicity in additive model (OR = 1.555; 95% CI: 1.041-2.323; P = 0.031), grade 3-4 thrombocytopenia (OR = 3.562; 95% CI: 1.513-8.390; P = 0.004). *XPG* rs1047768: increased risk of grade 3-4 leukocytopenia in additive model (OR = 1.701; 95% CI: 1.021-2.835; P = 0.041). *XPG* rs17655: increased risk of grade 3-4 thrombocytopenia in additive model (OR = 2.165; 95% CI: 1.191-3.938; P = 0.011). *APE1* rs1130409: decreased risk of grade 3-4 leukocytopenia in dominant model (OR = 0.460; 95% CI: 0.241-0.879; P = 0.019), grade 3-4 neutropenia in dominant model (OR = 0.557; 95% CI: 0.321-0.967; P = 0.038). *XRCC1* rs25487: increased risk of grade 3-4 leukocytopenia in recessive model (OR = 2.841; 95% CI: 1.051-7.681; P = 0.040), grade 3-4 thrombocytopenia in additive model (OR = 2.033; 95% CI: 1.113-3.715; P = 0.021). *MDM2* rs2279744: decreased risk of grade 3-4 thrombocytopenia in additive model (OR = 0.472; 95% CI: 0.257-0.866; P = 0.015). *RAD51* rs1801320: decreased risk of grade 3-4 hematologic toxicity in additive model (OR = 0.552; 95% CI: 0.330-0.924; P = 0.024). *RAD51* rs12593359: decreased risk of grade 3-4 leukocytopenia in dominant model (OR = 0.434; 95% CI: 0.199-0.949; P = 0.037). | 8 |
| Björn et al.2018[21] | 215 in the discovery cohort) and validated in an independent genome-wide association study NSCLC  CBP + GEM | 148,148 variants | Grade 3-4 thrombocytopenia (34.88%) | These analyses identified 130 SNVs/INDELs and 25 genes associated with thrombocytopenia (P-value < 0.002). Twenty-three SNVs were validated in an independent genome-wide association study (GWAS). | 7 |
| De Troia et al.2018[65] | 82 NSCLC+SCLC  DDP/CBP + VP-16/NVB DDP + GEM/PEM/TXT DDP monotherapy | *ABCB1/MDR1* rs1045642 (C3435T) *ABCC2/MRP2* rs717620 (C-24T) *GSTP1* rs1695 (A313G, Ile105Val) | Grade 3-4 hematological toxicity (25.6%) | *ABCB1/MDR1* rs1045642 (C3435T): decreased risk of grade 3-4 hematological toxicity (CT vs. CC OR = 0.18; 95% CI 0.05-0.65; P = 0.01, CT + TT vs. CC OR = 0.24; 95% CI: 0.07-0.75; P = 0.01). | 5 |
| Li et al.2018[107] | 427 NSCLC  DDP/CBP + GEM/PEM/TXT/NVB/PTX | *ATP7A* rs2227291 *ATP7A* rs6622665 *ATP7B* rs1061472 *ATP7B* rs9535826 | Grade 3-4 hematological toxicity (23.7%) | No significant association. | 6 |
| Sun et al.2018[57] | 1004 NSCLC  DDP/CBP + NVB/GEM/PTX/TXT Others DDP/CBP combinations | 8 tagging and potentially functional SNPs of *SLC31A1* gene | Grade 3-4 hematological toxicity (23.9%) Grade 3-4 anemia (3.1%) Grade 3-4 neutropenia (12.3%) Grade 3-4 leukocytopenia (15.2%) Grade 3-4 thrombocytopenia (3.6%) | *SLC31A1* rs4979223: grade 3-4 thrombocytopenia (A/C vs. C/C OR= 4.90; 95% CI:1.43-16.82; P = 0.012, A/C vs. A/A + C/C OR = 2.60; 95% CI: 1.20-5.64; P = 0.015).  *SLC31A1* rs4978536: grade 3-4 thrombocytopenia (A/G vs. A/A OR= 2.71; 95% CI: 1.26-5.81; P = 0.010, A/G + G/G vs. A/A OR = 2.59; 95% CI: 1.23-5.47; P = 0.012). *SLC31A1* rs10817464: grade 3-4 leucopenia (A/G vs. A/A OR= 2.21; 95% CI: 1.22−4.01; P =0.009, A/G + G/G vs. A/A OR = 2.16; 95% CI: 1.19-3.92; P = 0.011), grade 3-4 thrombocytopenia (A/G + G/G vs. A/A OR = 3.09; 95% CI: 1.20-7.93; P = 0.019) and grade 3-4 hematologic toxicity (A/G vs. A/A OR= 1.92; 95% CI: 1.15−3.21; P =0.012, A/G + G/G vs. A/A OR = 1.98; 95% CI: 1.19-3.29; P = 0.008). *SLC31A1* rs10759637: grade 3-4 thrombocytopenia (A/C vs. A/A OR= 5.10; 95% CI: 1.49−17.52; P =0.010, A/C vs. A/A + C/C OR = 2.69; 95% CI: 1.24-5.83; P = 0.012). | 8 |
| Yoshihama et al.2018[25] | 320 Ovarian fallopian tube, peritoneal, uterine, or cervical cancer  CBP + PTX | 1,013 variants | severe hematotoxicity (including neutropenia G4, thrombocytopenia ≥ G3, and anemia ≥ G3) (15.63%) | *GSTP1* rs1695: severe hematotoxicity (105Ile wild type allele vs. 105Val allele OR = 5.71; 95% CI: 1.77-18.44; P = 0.00034) | 5 |
| Gong et al.2019[119] | 467 NSCLC + SCLC  DDP/CBP + PEM/GEM/PTX /TXT/VP-16 Other DDP/CBP-based chemotherapy (CPT-11 + DDP/CBP, NVB +DDP/CBP) | *STAT3* rs4796793 | Grade 3-4 hematological toxicity (24.4%) | *STAT3* rs4796793: increased risk of grade 3-4 hematological toxicity in additive model (OR = 1.352; 95% CI: 1.001-1.826; P = 0.049). | 6 |
| Lavanderos et al.2019[80] | 119 Testicular cancer  DDP + BLM + VP-16 | *GSTT1* gene deletion *GSTM1* gene deletion *GSTP1* rs1695 (A313G, Ile105Val) *ERCC1* rs11615 (C118T, Asn118Asn) *ERCC1* rs3212986 (C8092A) *ERCC2/XPD* rs13181 (Lys751Gln) *ERCC2/XPD* rs1799793 (Asp312Asn) *ERCC2/XPD* rs238406 (Arg156Arg) *ABCB1/MDR1* rs1045642 (C3435T) | Grade 3-4 anemia (2.56%) Grade 3-4 neutropenia (39.66%) Grade 3-4 leukopenia (12.71%) Grade 3-4 thrombocytopenia (1.71%) Grade 3-4 lymphocytopenia (2.59%) Grade 3-4 febrile neutropenia (12.61%) | *ERCC2/XPD* rs238406 (C22541A, Arg156Arg): increased risk of grade 3-4 leukopenia (CA+ AA vs. CC OR = 4.09; 95% CI: 1.04-15.99; P = 0.043). *ERCC1* rs11615 (C118T, Asn118Asn): increased risk of grade 3-4 febrile neutropenia (TT vs. CC + CT OR = 4.89; 95% CI: 1.06-22.56; P = 0.042). | 5 |
| Liblab et al.2019[47] | 52 Ovarian cancer (Epithelial ovarian cancer)  CBP + PTX CBP mono-therapy | *ERCC1* rs3212986 (C8092A) *XPCC1* rs25487 (Arg399Gln) *GSTP1* rs1695 (A313G, Ile105Val) | Grade 2-4 anemia (53.8%) | *GSTP1* rs1695 (A313G, Ile105Val): associated with grade 2-4 anemia (the incident rate of grade 2-4 anemia for AA and AG were 46.34%, and 81.82%, P = 0.036). | 2 |
| Senk et al.2019[50] | 194 Malignant mesothelioma DDP + GEM/PEM | *AQP1* rs1049305 *AQP1* rs1476597 *AQP1* rs28362731 | Grade 2-4 anemia (55.4%) Grade 2-4 leukopenia (23.8%) Grade 2-4 neutropenia (37.7%) Grade 1-4 thrombocytopenia (15.2%) | *AQP1* rs28362731(G>A): grade 1-4 thrombocytopenia (GA vs. GG OR = 4.63; 95% CI: 1.13-19.05; P = 0.034). *AQP1* rs1049305(G>C): grade 2-4 anemia (GC vs. GG OR = 0.46; 95% CI: 0.23-0.92; P = 0.029), grade 2-4 anemia (GC + CC vs. GG OR = 0.52; 95% CI: 0.27-0.99; P = 0.046) and grade 2-4 leukopenia (CC vs. GG OR = 3.03; 95% CI: 1.10-8.38; P = 0.033, GC + CC vs. GG OR = 2.09; 95% CI: 1.00-4.35; P = 0.049). | 5 |
| Björn et al.2020[24] | 96 (split up into 80% training and 20% validation) NSCLC  CBP + GEM | a total of 17,934,566 single-nucleotide variants (SNVs) and insertions/deletions (INDELs), | Grade 3-4 neutropenia (47.92%) Grade 3-4 leukopenia (29.17%) Grade 3-4 thrombocytopenia (56.25%) | Association of genetic variants in PLINK found 4594, 5019, and 5066 autosomal SNVs/INDELs with p ≤ 1 × 10−3 for neutropenia, leukopenia, and thrombocytopenia, respectively. this results propose the toxicity module, which is associated with maximal myelosuppressive toxicity, and a model for predicting this toxicity based on 62 genetic variants | 6 |
| Bushra et al.2020[74] | 285 NSCLC  DDP/CBP + GEM/NVB/PTX/TXT | *GSTP1* rs1695 (A313G, Ile105Val) *XRCC1* rs25487 (Arg399Gln) *XPC* rs2228001 (Lys939Gln)  *ERCC1* rs11615 (C118T, Asn118Asn) | Grade 3-4 anemia (33.68%) Grade 3-4 neutropenia (31.23%) Grade 3-4 leukopenia (24.91%) Grade 3-4 thrombocytopenia (16.49%) | *GSTP1* rs1695 (A313G, Ile105Val): decreased risk of grade 3-4 anemia (GG vs. AA OR = 0.29; 95% CI: 0.10-0.87; P = 0.027) and grade 3-4 neutropenia (GG vs. AA OR = 0.31; 95% CI: 0.10-0.96; P = 0.043). *XRCC1* rs25487(G23885A, Arg399Gln): increased risk of grade 3-4 anemia (AA + AG vs. GG OR = 2.0; 95% CI: 1.19-3.35; P = 0.008, AG vs. GG OR = 2.27; 95% CI: 1.32-3.91; P = 0.003), grade 3-4 neutropenia (AG vs. GG OR = 2.37; 95% CI: 1.37-4.07; P = 0.002, AA + AG vs. GG OR= 1.98; 95% CI: 1.18-3.33; P = 0.010), grade 3-4 leukopenia (AG vs. GG OR = 1.79; 95% CI: 1.0-3.18; P = 0.049) and grade 3-4 thrombocytopenia (AG vs. GG OR = 2.14; 95% CI: 1.09-4.20; P = 0.027, AA+AG vs. GG OR = 2.11; 95% CI: 1.10-4.06; P = 0.025). *XPC* rs2228001(A>C, Lys939Gln): decreased risk of grade 3-4 anemia (CC vs. AA OR = 0.18; 95% CI: 0.04-0.82; P = 0.027), increased risk of grade 3-4 neutropenia (AC vs. AA OR = 3.31; 95% CI: 1.74-6.31; P = 0.0003, AC + CC vs. AA OR = 2.63; 95% CI: 1.41-4.90; P = 0.002). | 5 |
| Ferracini et al.2020[41] | 112 Ovarian cancer (Epithelial ovarian cancer)  CBP + PTX CBP mono-therapy | *GSTP1* rs1695 (A313G, Ile105Val) *ABCB1/MDR1* rs1128503 (C1236T) *ABCB1/MDR1* rs1045642 (C3435T) *ABCB1/MDR1* rs2032582 (G2677TA) | Grade 3-4 anemia (11.6%) Grade 3-4 neutropenia (19.7%) Grade 1-4 thrombocytopenia (32.1%) | *GSTP1* rs1695 (A313G, Ile105Val): decreased risk of grade 3-4 anemia (AG vs. AA OR = 0.16; 95% CI: 0.03-0.84; P = 0.03, AG + GG vs. AA OR= 0.17; 95% CI: 0.04-0.69; P = 0.01), and grade 1-4 thrombocytopenia (AG vs. AA OR = 0.32; 95% CI: 0.12-0.82; P = 0.01, GG vs. AA OR = 0.11; 95% CI: 0.02-0.59; P < 0.01, AG + GG vs. AA OR = 0.27; 95% CI: 0.12-0.64; P < 0.01, GG vs. AA + AG OR = 0.18; 95% CI: 0.03-0.85; P = 0.03).  *ABCB1/MDR1* rs1128503 (C1236T): increased risk of grade 1-4 thrombocytopenia (TT vs. CC OR = 3.63; 95% CI: 0.98-13.47; P = 0.05, TT vs. CT + CC OR = 3.50; 95% CI: 1.12-10.97; P = 0.03). | 7 |
| Nomura et al.2020[32] | 158 Esophageal cancer  DDP + TXT + 5-FU | *ABCB1/MDR1* rs1128503 (C1236T) *ABCB1/MDR1* rs1045642 (C3435T) *ABCB1/MDR1* rs2032582 (G2677TA) *ABCC2/MRP2* rs12762549 (*+9383C>G) *ABCG2* rs2231142 (421C>A) *ABCG2* rs2231137 (34G>A) *GSTP1* rs1695 (A313G, Ile105Val) *GSTT1* gene deletion *GSTM1* gene deletion | Grade 3-4 neutropenia (51.27%) | *ABCB1/MDR1* rs1045642 (C3435T): increased risk of grade 3-4 neutropenia (CT + TT vs. CC OR = 2.191; 95% CI 1.087-4.417, P=0.028). *ABCC2/MRP2* rs12762549 (*+9383C>G): increased risk of grade 3-4 neutropenia (GG vs. GC + CC OR = 2.342; 95% CI 1.108-4.948, P = 0.026). | 6 |
| Svedberg et al.2020[23] | 215 (in discovery cohort) and 144 (in validation cohort) NSCLC CBP + GEM | Whole-exome sequencing | Grade 3-4 neutropenia Grade 3-4 leucopenia | Association analysis identified 50 and 111 SNVs, and 12 and 20 genes, for leukopenia and neutropenia, respectively. Of these SNVS 20 and 19 were partially validated for leukopenia and neutropenia, respectively. This study created wGRS models for predicting the risk of chemotherapy-induced hematological toxicity. | 7 |
| Nairuz et al.2021[39] | 180 Lung cancer  DDP/CBP + VP-16/PTX/TXT CBP + GEM/ADM | *ERCC2/XPD* rs13181 (Lys751Gln) *TP53* rs1042522 (C>G, Pro72Arg) | Grade 3-4 neutropenia (46.11%) Grade 3-4 leucopenia (36.11%) Grade 3-4 anemia (50.00%) Grade 3-4 thrombocytopenia (18.33%) | No significant association | 2 |
| Walia et al.2021a[34] | 317 NSCLC + SCLC  DDP/CBP + PEM/CPT-11/TXT/PTX/GEM | *GSTP1* rs1695 (A313G, Ile105Val) | Grade 3-4 anemia (10.4%) Grade 1-4 anemia (81.7%) Grade 2-4 anemia (46.37%) Grades 1-4 leukopenia (27.76%) Grades 2-4 leukopenia (7.57%) | *GSTP1* rs1695: grade 3-4 anemia (IIe105Val Ile/Val vs. Ile/Ile OR = 2.12; 95% CI: 0.97-4.62; P = 0.04) and grade 2-4 leukopenia (Val/Val vs. Ile/Ile OR = 2.41; 95% CI: 1.39-4.18; P = 0.001). | 7 |
| Walia et al.2021b[35] | 123 Lung adenocarcinoma cancer  DDP/CBP + PEM | *MTHFR* rs1801131(A1298C) *MTHFR* rs1801133(C677T) | Grade 1-3 neutropenia (13.27%) Grade 2-3 neutropenia (8.85%) | *MTHFR* rs1801133: grade 1-3 neutropenia (CT vs. CC OR = 5.34; 95% CI: 1.49-19.06; P = 0.009, CT + TT vs. CC OR = 4.45; 95% CI: 1.28-15.43; P = 0.019). | 7 |
| Wang et al.2021[69] | 1004 NSCLC  DDP/CBP + NVB/GEM/PTX/TXT Others DDP/CBP combinations | 13 tagging and functional SNPs of *ABCG2* | Grade 3-4 hematologic toxicity (23.9%) Grade 3-4 anemia (3.1%) Grade 3-4 neutropenia (12.3%) Grade 3-4 thrombocytopenia (3.6%) | *ABCG2* rs12505410: grade 3-4 neutropenia (CC vs. AA OR = 2.29; 95% CI: 1.28-4.10; P = 0.005, CC vs. AA + AC OR = 2.09; 95% CI: 1.23-3.57; P = 0.007). *ABCG2* rs1871744: grade 3-4 anemia (AG vs. AA OR = 0.37; 95% CI: 0.14-0.95; P = 0.039, AG + GG vs. AA OR = 0.42; 95% CI: 0.19-0.95; P = 0.038). *ABCG2* rs2231138: grade 3-4 anemia (AG vs. AA OR = 2.57; 95% CI: 1.12-5.89; P = 0.026, AG vs. AA+ GG OR = 2.60; 95% CI: 1.13-5.94; P = 0.024). | 7 |
| Zheng et al.2021[117] | 437 NSCLC  DDP/CBP + NVB/GEM/PTX/TXT/PEM | *EPO* rs1617640 | Grade 3-4 hematologic toxicity (24.0%) Grade 3-4 leukocytopenia (10.1%) Grade 3-4 neutropenia (16.0%) Grade 3-4 thrombocytopenia (5.9%) Grade 3-4 anemia (7.3%%) | *EPO* rs1617640: grade 3-4 hematologic toxicity (TT vs. GG + GT OR = 1.783; 95% CI: 1.098-2.898; P = 0.019, TT vs. GG OR = 4.702; 95% CI: 1.048-21.095; P = 0.043). | 6 |

Abbreviations: ADM: doxorubicin; BEV: bevacizumab; BLM: bleomycin; CAP: capecitabine; CBP: carboplatin; CI: confidence interval; CPT-11: irinotecan; CTCAE: Common Terminology Criteria for Adverse Events; CTX: cyclophosphamide; DDP: cisplatin; EC-GemCap: epirubicin cisplatin (intra-arterial infusion)-gemcitabine capecitabine; GEM: gemcitabine; L-OHP: oxaliplatin; LV: leucovorin; MMC: mitomycin C; NSCLC: non-small cell lung cancer; NVB: navelbine; OR: odds ratios; PDXG: cisplatin, docetaxel capecitabine, gemcitabine; PEM: pemetrexed; PEXG: cisplatin, epirubicin, capecitabine, gemcitabine; PTX: paclitaxel; SCLC: small cell lung cancer; TXT: docetaxel; VCR: vincristine; VDS: vindesine; VP-16: etoposide; 5-FU: fluorouracil.
