# Supplementary material for: Genetic polymorphisms and platinum-induced hematological toxicity: a systematic review
Source: Front Pharmacol. 2024 Aug 21;15:1445328. doi: 10.3389/fphar.2024.1445328 (PMC11371761; doi:10.3389/fphar.2024.1445328)
Supplement: Supplementary file 8 [file Table6.docx]

Supplementary Material

## Supplementary Table 6 Quality assessment results

| Study | 1.Quality of clinical information | | 2.Quality of genotyping | | 3.Quality in reporting of study population origin | | 4.Quality in terms of sample size and statistical correction for multiple testing | | 5.Quality of study analysis | | Total |
| --- | --- | --- | --- | --- | --- | --- | --- | --- | --- | --- | --- |
|  | 1a | 1b | 2a | 2b | 3a | 3b | 4a | 4b | 5a | 5b |  |
| Isla et al.2004[54] | 1 | 1 | 0 | 0 | 0 | 0 | 0 | 0 | 0 | 0 | 2 |
| Han et al.2006[55] | 0 | 1 | 1 | 0 | 0 | 0 | 0 | 0 | 0 | 0 | 2 |
| KimCurran et al.2011[145] | 1 | 1 | 1 | 0 | 1 | 0 | 0 | 0 | 0 | 0 | 4 |
| Marsh et al.2007[29] | 1 | 1 | 1 | 0 | 0 | 0 | 0 | 1 | 0 | 0 | 4 |
| Tibaldi et al.2008[30] | 1 | 1 | 1 | 0 | 0 | 0 | 0 | 0 | 0 | 0 | 3 |
| Wang et al.2008[90] | 0 | 1 | 0 | 0 | 1 | 0 | 0 | 0 | 1 | 1 | 4 |
| Kim et al.2009[36] | 1 | 1 | 1 | 0 | 0 | 0 | 0 | 0 | 1 | 1 | 5 |
| Seo et al.2009[52] | 1 | 1 | 1 | 0 | 0 | 0 | 0 | 0 | 0 | 0 | 3 |
| Wu et al.2009[83] | 1 | 1 | 1 | 0 | 1 | 0 | 0 | 1 | 1 | 1 | 7 |
| Chen et al.2010[53] | 1 | 0 | 1 | 0 | 1 | 0 | 0 | 0 | 0 | 0 | 3 |
| Giovannetti et al.2011[26] | 1 | 1 | 1 | 0 | 1 | 0 | 0 | 1 | 0 | 0 | 5 |
| Han et al.2011[62] | 1 | 1 | 1 | 1 | 1 | 0 | 0 | 0 | 1 | 1 | 7 |
| Ludovini et al.2011[28] | 1 | 1 | 1 | 1 | 0 | 0 | 0 | 0 | 1 | 1 | 6 |
| Zhao et al.2011[123] | 1 | 1 | 1 | 1 | 1 | 0 | 0 | 1 | 1 | 1 | 8 |
| Erčulj et al.2012[48] | 1 | 1 | 1 | 0 | 1 | 0 | 0 | 0 | 1 | 1 | 6 |
| Gu et al.2012[115] | 1 | 1 | 1 | 1 | 1 | 0 | 0 | 1 | 1 | 1 | 8 |
| Iwata et al.2012[31] | 0 | 1 | 0 | 0 | 1 | 0 | 0 | 0 | 0 | 0 | 2 |
| Khrunin et al.2012[40] | 1 | 1 | 0 | 0 | 0 | 0 | 0 | 1 | 0 | 1 | 4 |
| Qian et al.2012[44] | 1 | 1 | 1 | 1 | 1 | 0 | 0 | 1 | 1 | 1 | 8 |
| Xu et al.2012[58] | 1 | 1 | 1 | 0 | 1 | 0 | 0 | 1 | 0 | 0 | 5 |
| Zhan et al.2012[112] | 1 | 1 | 1 | 0 | 1 | 0 | 0 | 0 | 1 | 1 | 6 |
| Cortejoso et al.2013[51] | 0 | 1 | 1 | 1 | 1 | 0 | 0 | 0 | 1 | 1 | 6 |
| Goričar et al.2013[49] | 0 | 1 | 1 | 0 | 1 | 0 | 0 | 0 | 1 | 1 | 5 |
| Lee et al.2013[37] | 1 | 1 | 1 | 0 | 1 | 0 | 0 | 0 | 1 | 1 | 6 |
| Li et al.2013[100] | 1 | 1 | 1 | 1 | 1 | 0 | 0 | 1 | 1 | 1 | 8 |
| Low et al.2013[19] | 0 | 0 | 1 | 1 | 1 | 1 | 0 | 0 | 0 | 1 | 5 |
| Peng et al.2013[118] | 0 | 1 | 1 | 1 | 1 | 0 | 0 | 1 | 1 | 1 | 7 |
| Cai et al.2014[127] | 1 | 1 | 1 | 1 | 1 | 0 | 0 | 1 | 0 | 0 | 6 |
| Chen et al.2014[120] | 0 | 1 | 1 | 1 | 1 | 0 | 0 | 0 | 1 | 1 | 6 |
| Corrigan et al.2014[42] | 0 | 1 | 1 | 1 | 1 | 1 | 0 | 1 | 1 | 1 | 8 |
| Kanazawa et al.2014[99] | 1 | 1 | 1 | 1 | 0 | 0 | 0 | 0 | 0 | 0 | 4 |
| Peng et al.2014[88] | 1 | 1 | 0 | 1 | 1 | 0 | 0 | 1 | 1 | 1 | 7 |
| Ruzzo et al.2014[33] | 1 | 1 | 1 | 1 | 1 | 0 | 1 | 0 | 0 | 1 | 7 |
| Shao et al.2014[130] | 1 | 1 | 1 | 0 | 1 | 0 | 0 | 0 | 1 | 1 | 6 |
| Tan et al.2014[128] | 1 | 1 | 0 | 1 | 1 | 0 | 0 | 1 | 1 | 1 | 7 |
| Wang et al.2014[104] | 1 | 1 | 0 | 1 | 1 | 0 | 0 | 0 | 1 | 1 | 6 |
| Zhao et al.2014[121] | 1 | 1 | 1 | 1 | 1 | 0 | 0 | 1 | 1 | 1 | 8 |
| Zheng et al.2014[110] | 1 | 1 | 1 | 0 | 1 | 0 | 0 | 1 | 1 | 1 | 7 |
| Cao et al.2015[18] | 1 | 1 | 1 | 1 | 1 | 0 | 0 | 0 | 1 | 1 | 7 |
| Chen et al.2015[108] | 0 | 1 | 1 | 1 | 1 | 0 | 0 | 0 | 1 | 1 | 6 |
| Deng et al.2015[89] | 1 | 1 | 1 | 0 | 0 | 0 | 0 | 0 | 1 | 1 | 5 |
| Gréen et al.2015[22] | 1 | 1 | 1 | 1 | 1 | 0 | 0 | 1 | 0 | 1 | 7 |
| Huang et al.2015[20] | 0 | 1 | 1 | 1 | 0 | 1 | 0 | 0 | 1 | 1 | 6 |
| Kalikaki et al.2015[146] | 1 | 1 | 1 | 1 | 0 | 0 | 0 | 0 | 0 | 0 | 4 |
| Lambrechts et al.2015[27] | 1 | 1 | 1 | 1 | 1 | 0 | 0 | 1 | 1 | 1 | 8 |
| Qian et al.2015[109] | 0 | 1 | 1 | 1 | 1 | 0 | 0 | 1 | 1 | 1 | 7 |
| Ye et al.2015[96] | 1 | 1 | 1 | 0 | 1 | 0 | 0 | 1 | 1 | 1 | 7 |
| Yin et al.2015[125] | 0 | 1 | 1 | 1 | 1 | 0 | 0 | 0 | 0 | 1 | 5 |
| Chu et al.2016[97] | 1 | 1 | 1 | 0 | 1 | 0 | 0 | 0 | 1 | 1 | 6 |
| Fang et al.2016[111] | 0 | 1 | 1 | 1 | 1 | 0 | 0 | 1 | 1 | 1 | 7 |
| Guo et al.2016[105] | 0 | 1 | 0 | 0 | 1 | 0 | 0 | 1 | 1 | 1 | 5 |
| Hu et al.2016[114] | 0 | 1 | 1 | 1 | 1 | 0 | 0 | 0 | 1 | 1 | 6 |
| Jia et al.2016[43] | 1 | 1 | 0 | 1 | 1 | 0 | 0 | 0 | 1 | 1 | 6 |
| Kumpiro et al.2016[38] | 0 | 1 | 1 | 0 | 1 | 0 | 0 | 0 | 0 | 0 | 3 |
| Qian et al.2016[63] | 0 | 1 | 1 | 1 | 1 | 0 | 0 | 0 | 1 | 1 | 6 |
| Song et al.2016[81] | 0 | 1 | 0 | 0 | 1 | 0 | 0 | 1 | 1 | 1 | 5 |
| Wang et al.2016[124] | 1 | 1 | 1 | 1 | 1 | 0 | 0 | 1 | 1 | 1 | 8 |
| Xu et al.2016[122] | 0 | 1 | 1 | 1 | 1 | 0 | 0 | 0 | 1 | 1 | 6 |
| Yin et al.2016[45] | 0 | 1 | 1 | 1 | 1 | 0 | 0 | 0 | 0 | 1 | 5 |
| Zou et al.2016[126] | 0 | 1 | 1 | 1 | 1 | 0 | 0 | 0 | 1 | 1 | 6 |
| Gong et al.2017[113] | 0 | 1 | 1 | 1 | 1 | 0 | 0 | 0 | 1 | 1 | 6 |
| Liu et al.2017[116] | 1 | 1 | 0 | 0 | 1 | 0 | 0 | 1 | 1 | 1 | 6 |
| Liu et al.2017[129] | 0 | 1 | 1 | 1 | 1 | 0 | 0 | 0 | 1 | 1 | 6 |
| Zheng et al.2017[46] | 1 | 1 | 1 | 1 | 1 | 0 | 0 | 1 | 1 | 1 | 8 |
| Björn et al.2018[21] | 1 | 1 | 1 | 1 | 1 | 0 | 0 | 1 | 0 | 1 | 7 |
| De Troia et al.2018[65] | 0 | 1 | 1 | 0 | 1 | 0 | 0 | 0 | 1 | 1 | 5 |
| Li et al.2018[107] | 0 | 1 | 1 | 1 | 1 | 0 | 0 | 0 | 1 | 1 | 6 |
| Sun et al.2018[57] | 1 | 1 | 1 | 1 | 1 | 0 | 0 | 1 | 1 | 1 | 8 |
| Yoshihama et al.2018[25] | 1 | 0 | 1 | 1 | 1 | 0 | 0 | 0 | 0 | 1 | 5 |
| Gong et al.2019[119] | 0 | 1 | 1 | 1 | 1 | 0 | 0 | 0 | 1 | 1 | 6 |
| Lavanderos et al.2019[80] | 1 | 1 | 0 | 0 | 1 | 0 | 0 | 0 | 1 | 1 | 5 |
| Liblab et al.2019[47] | 1 | 0 | 0 | 0 | 1 | 0 | 0 | 0 | 0 | 0 | 2 |
| Senk et al.2019[50] | 0 | 1 | 1 | 0 | 1 | 0 | 0 | 0 | 1 | 1 | 5 |
| Björn et al.2020[24] | 1 | 1 | 1 | 1 | 1 | 0 | 0 | 1 | 0 | 0 | 6 |
| Bushra et al.2020[74] | 0 | 1 | 0 | 1 | 1 | 0 | 0 | 0 | 1 | 1 | 5 |
| Ferracini et al.2020[41] | 1 | 1 | 1 | 1 | 1 | 0 | 0 | 0 | 1 | 1 | 7 |
| Nomura et al.2020[32] | 1 | 1 | 1 | 0 | 1 | 0 | 0 | 0 | 1 | 1 | 6 |
| Svedberg et al.2020[23] | 1 | 1 | 1 | 1 | 1 | 0 | 0 | 1 | 1 | 0 | 7 |
| Nairuz et al.2021[39] | 0 | 0 | 0 | 0 | 1 | 0 | 0 | 0 | 0 | 1 | 2 |
| Wang et al.2021[69] | 1 | 1 | 1 | 1 | 1 | 0 | 0 | 0 | 1 | 1 | 7 |
| Zheng et al.2021[117] | 0 | 1 | 1 | 1 | 1 | 0 | 0 | 0 | 1 | 1 | 6 |
| Walia et al.2021a[34] | 1 | 1 | 1 | 1 | 1 | 0 | 0 | 0 | 1 | 1 | 7 |
| Walia et al.2021b[35] | 1 | 1 | 1 | 1 | 1 | 0 | 0 | 0 | 1 | 1 | 7 |
